# Supplementary figures and images for: Diverse homeostatic and immunomodulatory roles of immune cells in the developing mouse lung at single cell resolution
Source: eLife. 2020 Jun 2;9:e56890. doi: 10.7554/eLife.56890 (PMC7358008; doi:10.7554/eLife.56890)

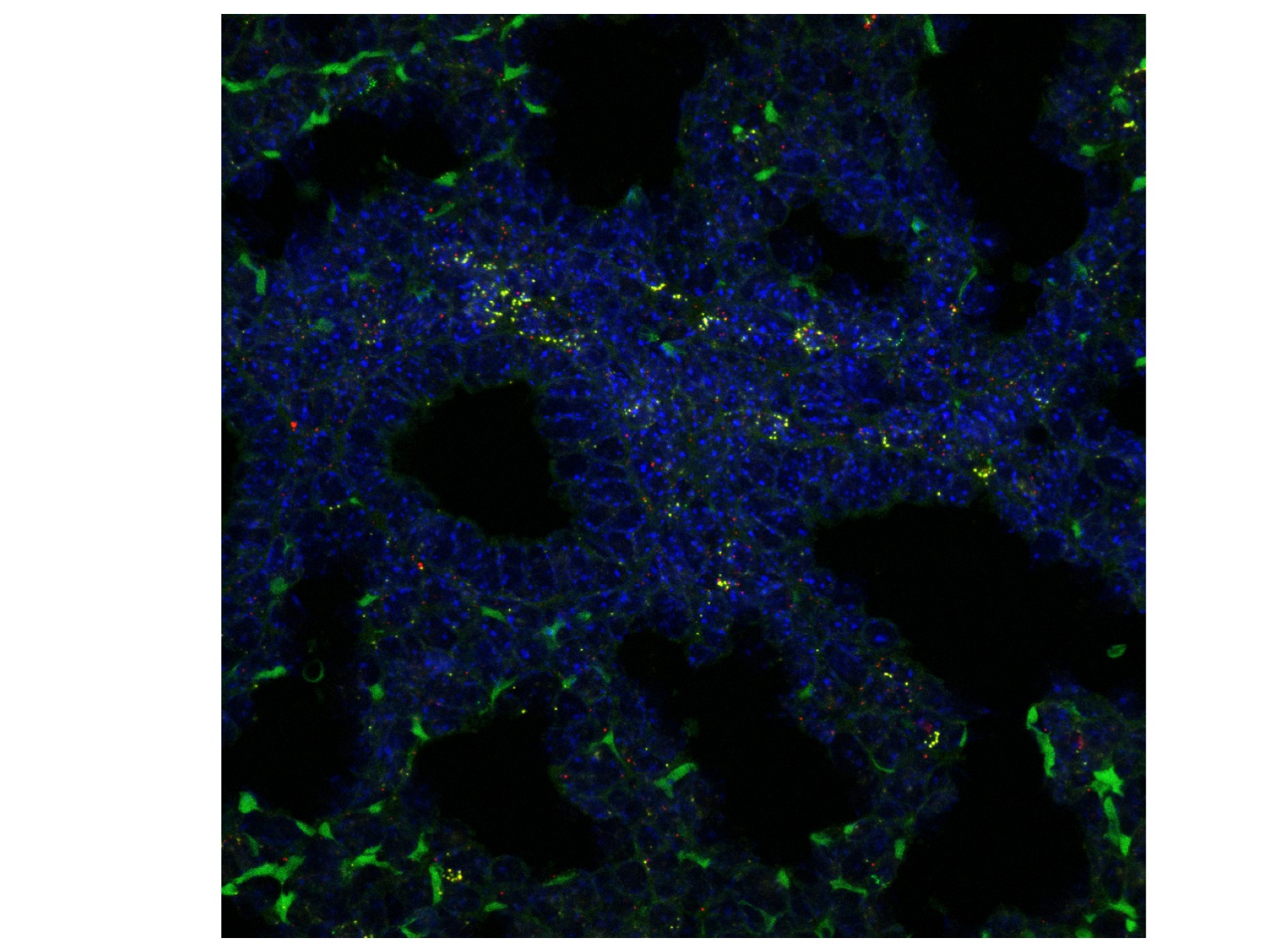

Supplement: Figure 2—source data 1. — This zip archive contains all the fluorescent micrographs used for the quantitative analysis shown in Fig. blank. The individual files are named with the timepoint (for figures containing more than one timepoint), the gene detected by FISH, followed by the color of the label for the gene with ‘G’ for green, ‘R’ for red, ‘W’ for white, and ‘Y’ for yellow. [file elife-56890-fig2-data1.zip › Source FIles for Dab2 and Plac8 JPEG/Slide10_E18.5_Cd68Y_Dab2R_Plac8G.jpeg]

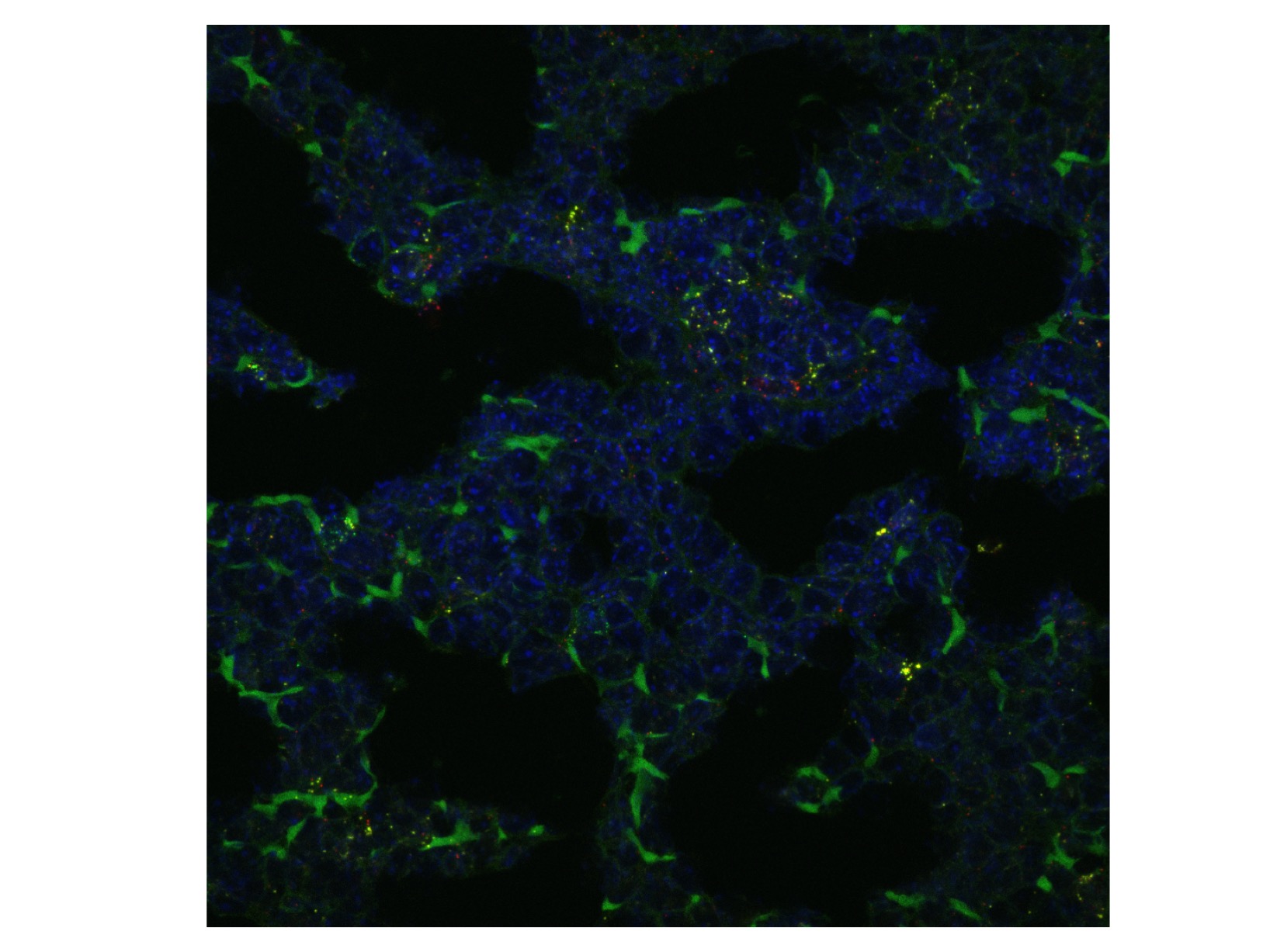

Supplement: Figure 2—source data 1. — This zip archive contains all the fluorescent micrographs used for the quantitative analysis shown in Fig. blank. The individual files are named with the timepoint (for figures containing more than one timepoint), the gene detected by FISH, followed by the color of the label for the gene with ‘G’ for green, ‘R’ for red, ‘W’ for white, and ‘Y’ for yellow. [file elife-56890-fig2-data1.zip › Source FIles for Dab2 and Plac8 JPEG/Slide11_E18.5_Cd68Y_Dab2R_Plac8G.jpeg]

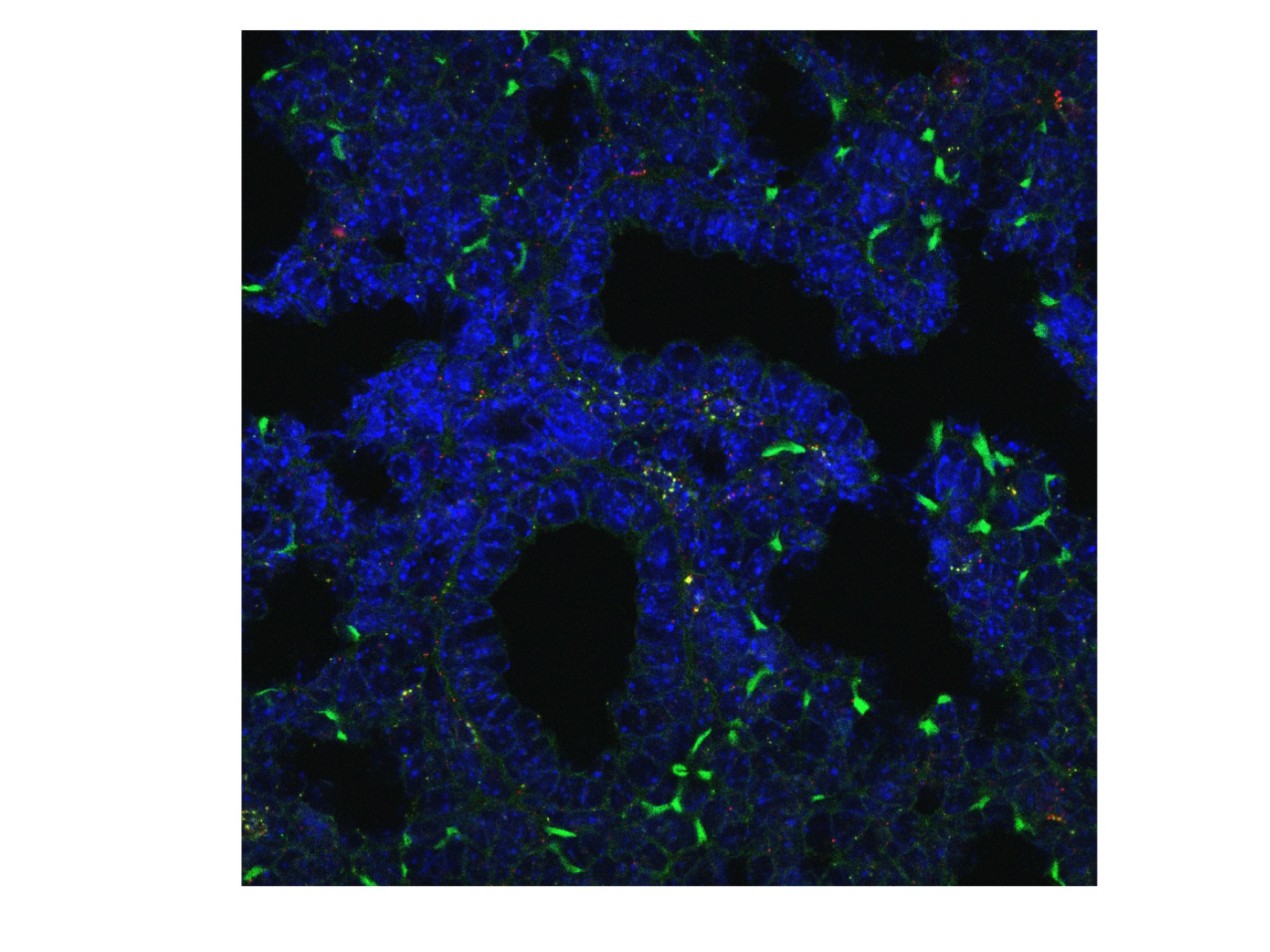

Supplement: Figure 2—source data 1. — This zip archive contains all the fluorescent micrographs used for the quantitative analysis shown in Fig. blank. The individual files are named with the timepoint (for figures containing more than one timepoint), the gene detected by FISH, followed by the color of the label for the gene with ‘G’ for green, ‘R’ for red, ‘W’ for white, and ‘Y’ for yellow. [file elife-56890-fig2-data1.zip › Source FIles for Dab2 and Plac8 JPEG/Slide12_E18.5_Cd68Y_Dab2R_Plac8G.jpeg]

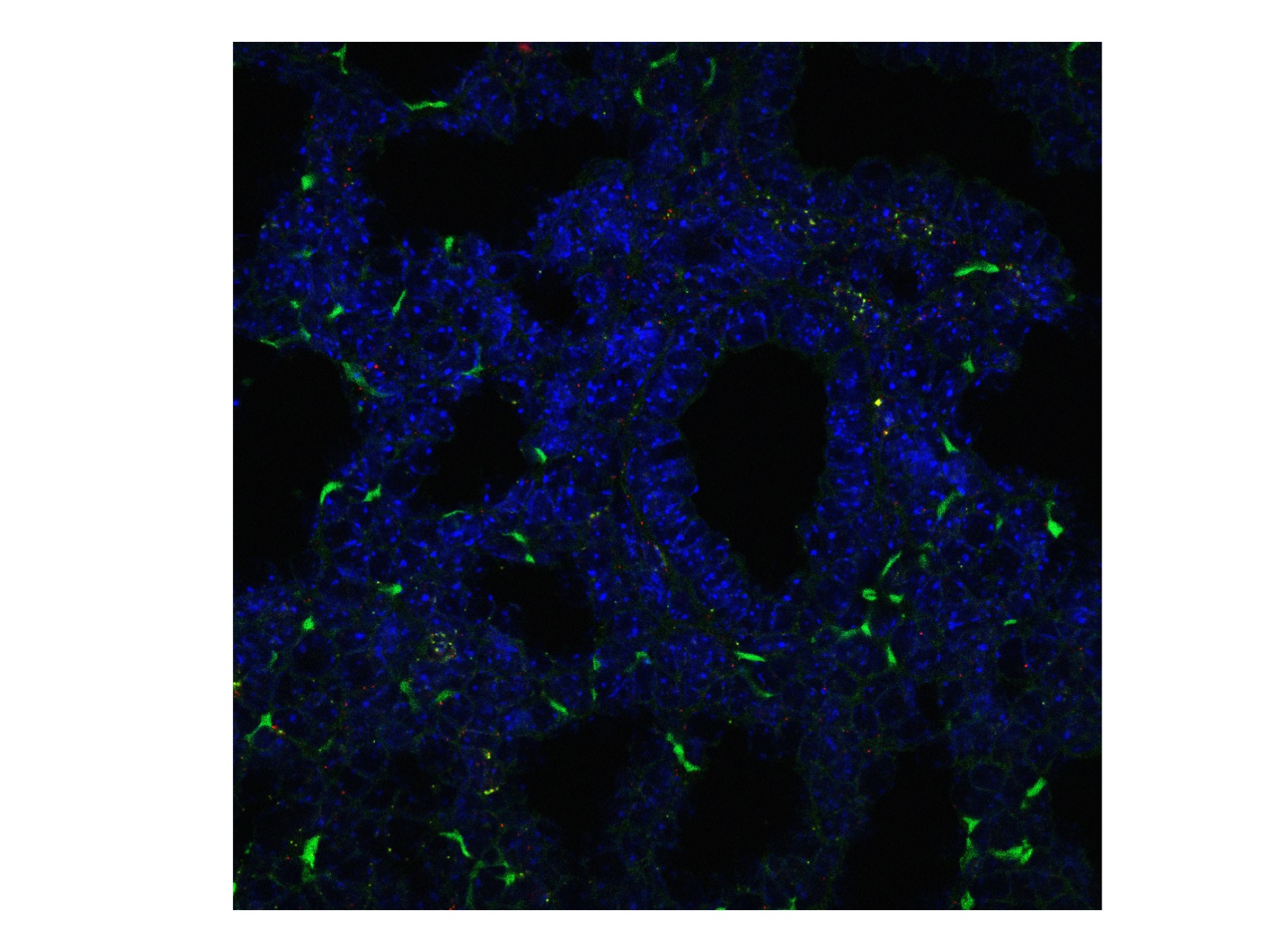

Supplement: Figure 2—source data 1. — This zip archive contains all the fluorescent micrographs used for the quantitative analysis shown in Fig. blank. The individual files are named with the timepoint (for figures containing more than one timepoint), the gene detected by FISH, followed by the color of the label for the gene with ‘G’ for green, ‘R’ for red, ‘W’ for white, and ‘Y’ for yellow. [file elife-56890-fig2-data1.zip › Source FIles for Dab2 and Plac8 JPEG/Slide13_E18.5_Cd68Y_Dab2R_Plac8G.jpeg]

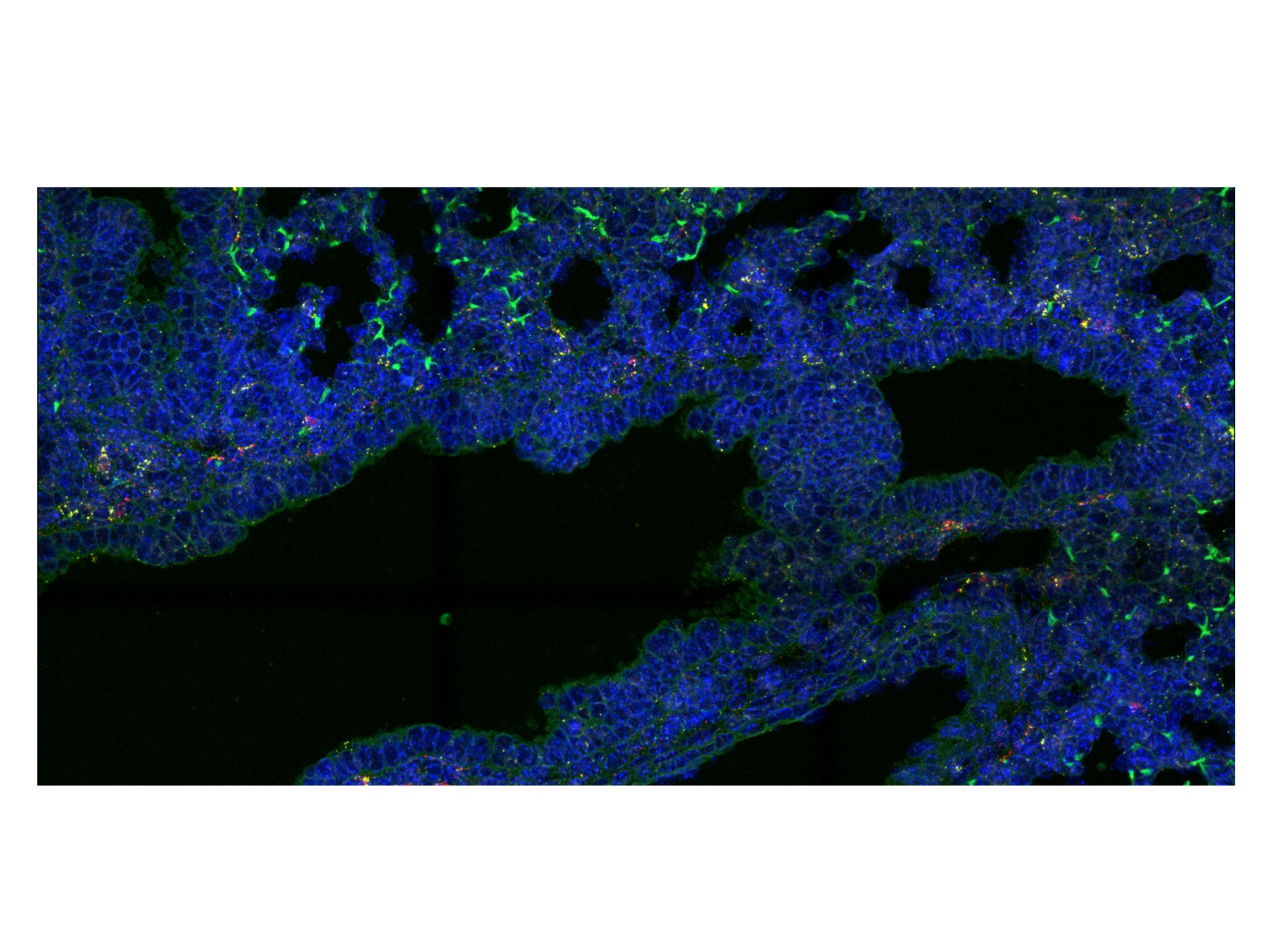

Supplement: Figure 2—source data 1. — This zip archive contains all the fluorescent micrographs used for the quantitative analysis shown in Fig. blank. The individual files are named with the timepoint (for figures containing more than one timepoint), the gene detected by FISH, followed by the color of the label for the gene with ‘G’ for green, ‘R’ for red, ‘W’ for white, and ‘Y’ for yellow. [file elife-56890-fig2-data1.zip › Source FIles for Dab2 and Plac8 JPEG/Slide14_E18.5_Cd68Y_Dab2R_Plac8G.jpeg]

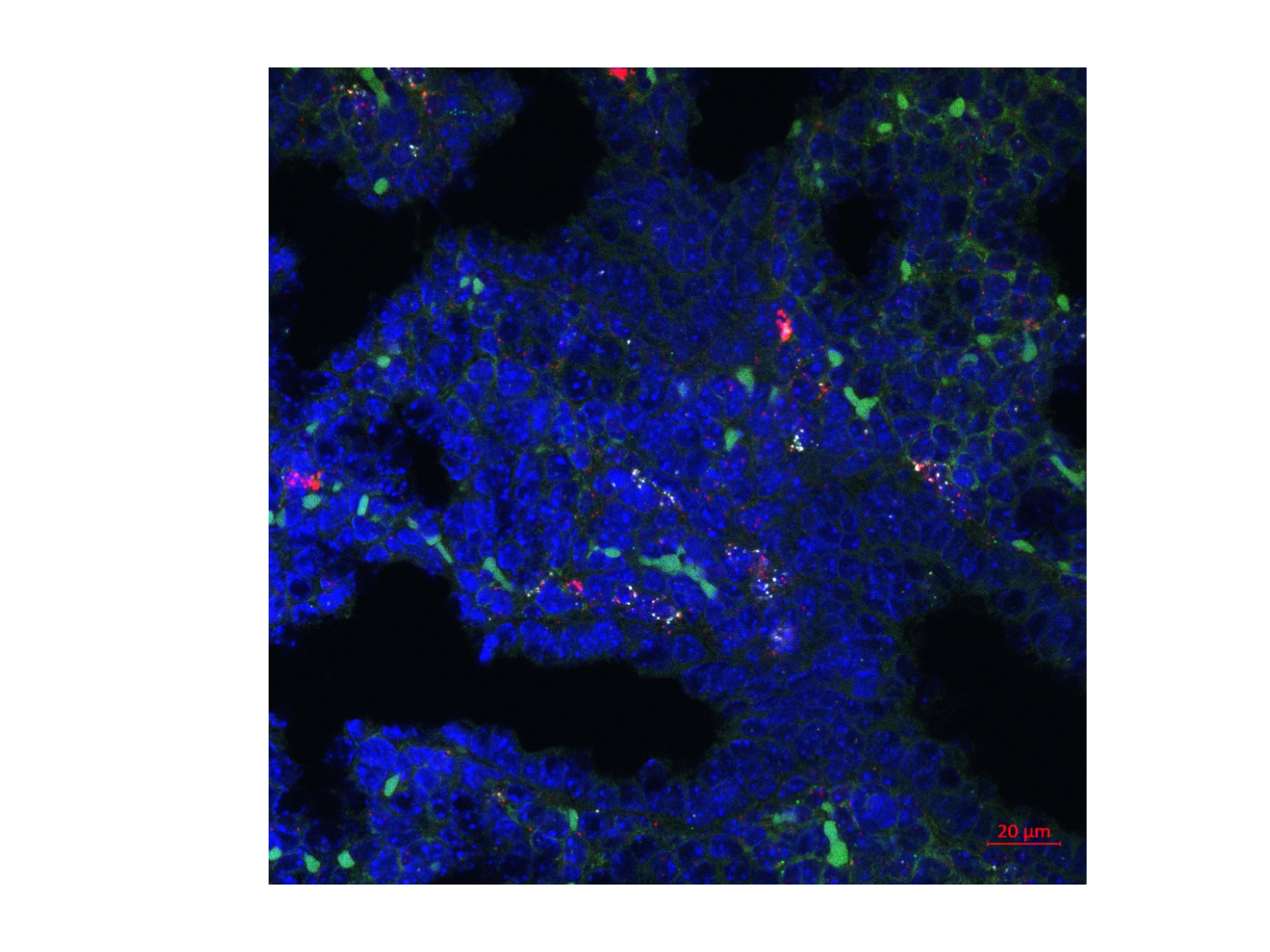

Supplement: Figure 2—source data 1. — This zip archive contains all the fluorescent micrographs used for the quantitative analysis shown in Fig. blank. The individual files are named with the timepoint (for figures containing more than one timepoint), the gene detected by FISH, followed by the color of the label for the gene with ‘G’ for green, ‘R’ for red, ‘W’ for white, and ‘Y’ for yellow. [file elife-56890-fig2-data1.zip › Source FIles for Dab2 and Plac8 JPEG/Slide1_E18.5_Cd68W_Dab2R_Plac8G.jpeg]

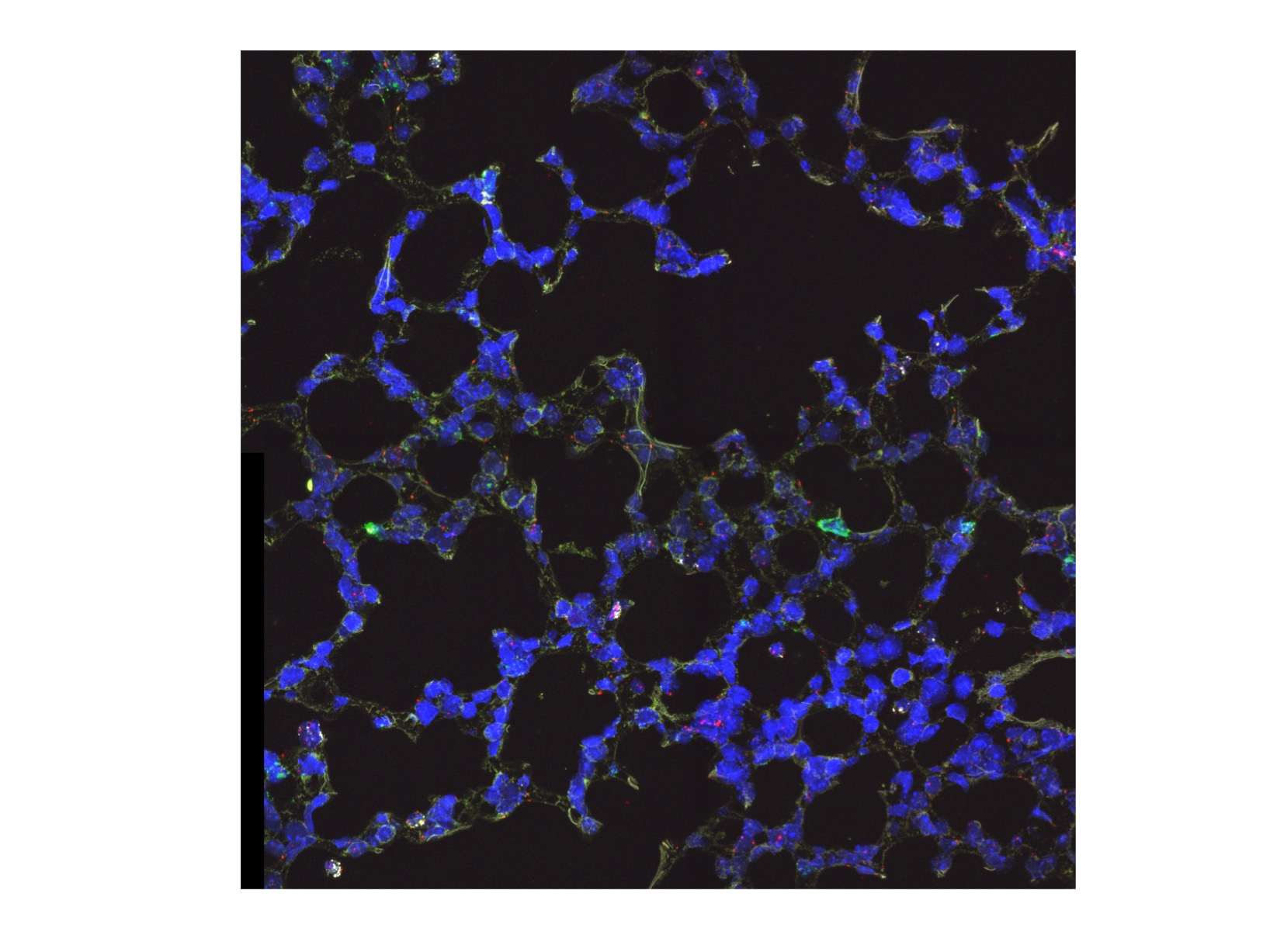

Supplement: Figure 2—source data 1. — This zip archive contains all the fluorescent micrographs used for the quantitative analysis shown in Fig. blank. The individual files are named with the timepoint (for figures containing more than one timepoint), the gene detected by FISH, followed by the color of the label for the gene with ‘G’ for green, ‘R’ for red, ‘W’ for white, and ‘Y’ for yellow. [file elife-56890-fig2-data1.zip › Source FIles for Dab2 and Plac8 JPEG/Slide1_P7_Cd68W_Dab2R-Plac8G.jpeg]

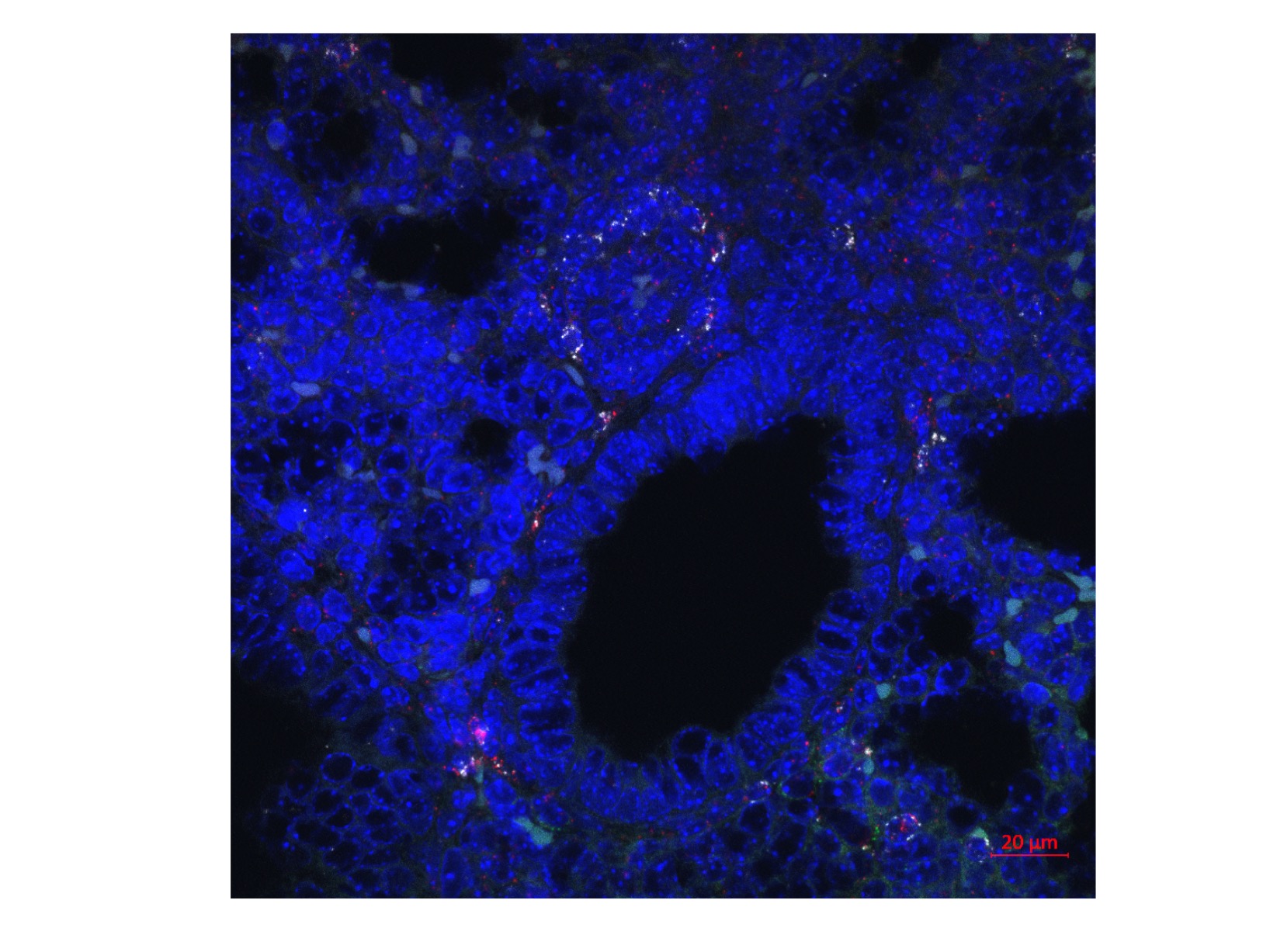

Supplement: Figure 2—source data 1. — This zip archive contains all the fluorescent micrographs used for the quantitative analysis shown in Fig. blank. The individual files are named with the timepoint (for figures containing more than one timepoint), the gene detected by FISH, followed by the color of the label for the gene with ‘G’ for green, ‘R’ for red, ‘W’ for white, and ‘Y’ for yellow. [file elife-56890-fig2-data1.zip › Source FIles for Dab2 and Plac8 JPEG/Slide2_E18.5_Cd68W_Dab2R_Plac8G.jpeg]

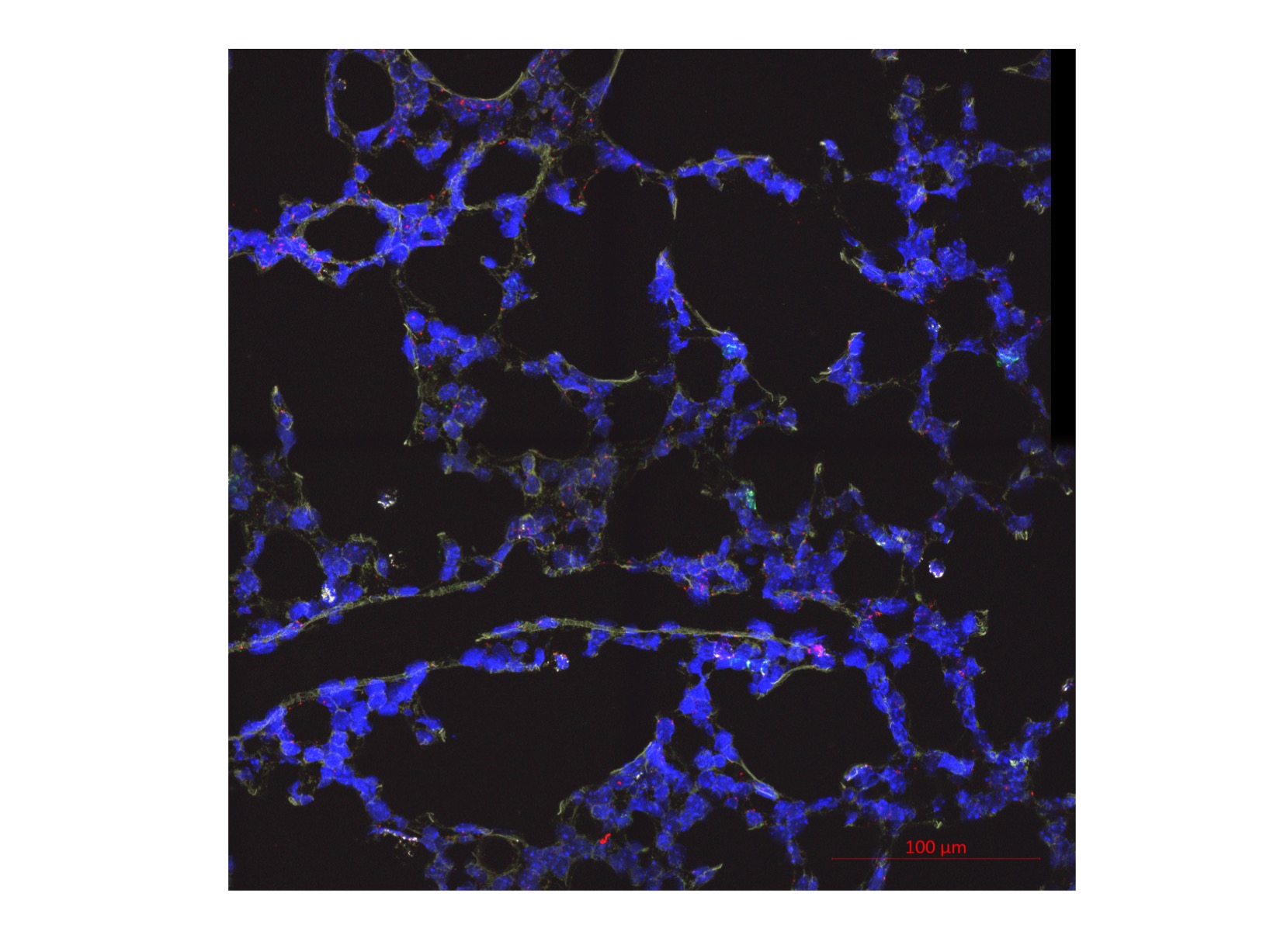

Supplement: Figure 2—source data 1. — This zip archive contains all the fluorescent micrographs used for the quantitative analysis shown in Fig. blank. The individual files are named with the timepoint (for figures containing more than one timepoint), the gene detected by FISH, followed by the color of the label for the gene with ‘G’ for green, ‘R’ for red, ‘W’ for white, and ‘Y’ for yellow. [file elife-56890-fig2-data1.zip › Source FIles for Dab2 and Plac8 JPEG/Slide2_P7_Cd68W_Dab2R-Plac8G.jpeg]

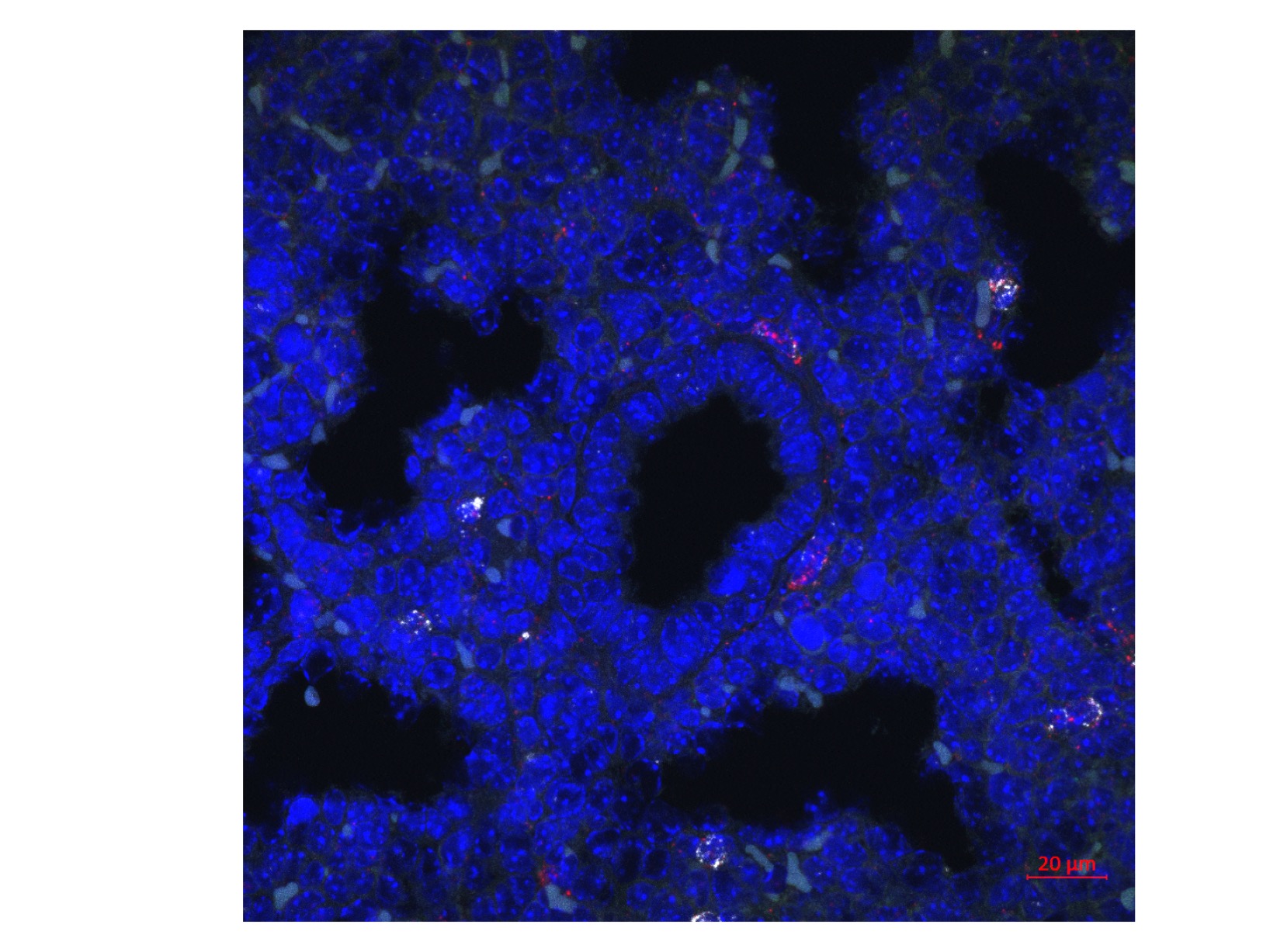

Supplement: Figure 2—source data 1. — This zip archive contains all the fluorescent micrographs used for the quantitative analysis shown in Fig. blank. The individual files are named with the timepoint (for figures containing more than one timepoint), the gene detected by FISH, followed by the color of the label for the gene with ‘G’ for green, ‘R’ for red, ‘W’ for white, and ‘Y’ for yellow. [file elife-56890-fig2-data1.zip › Source FIles for Dab2 and Plac8 JPEG/Slide3_E18.5_Cd68W_Dab2R_Plac8G.jpeg]

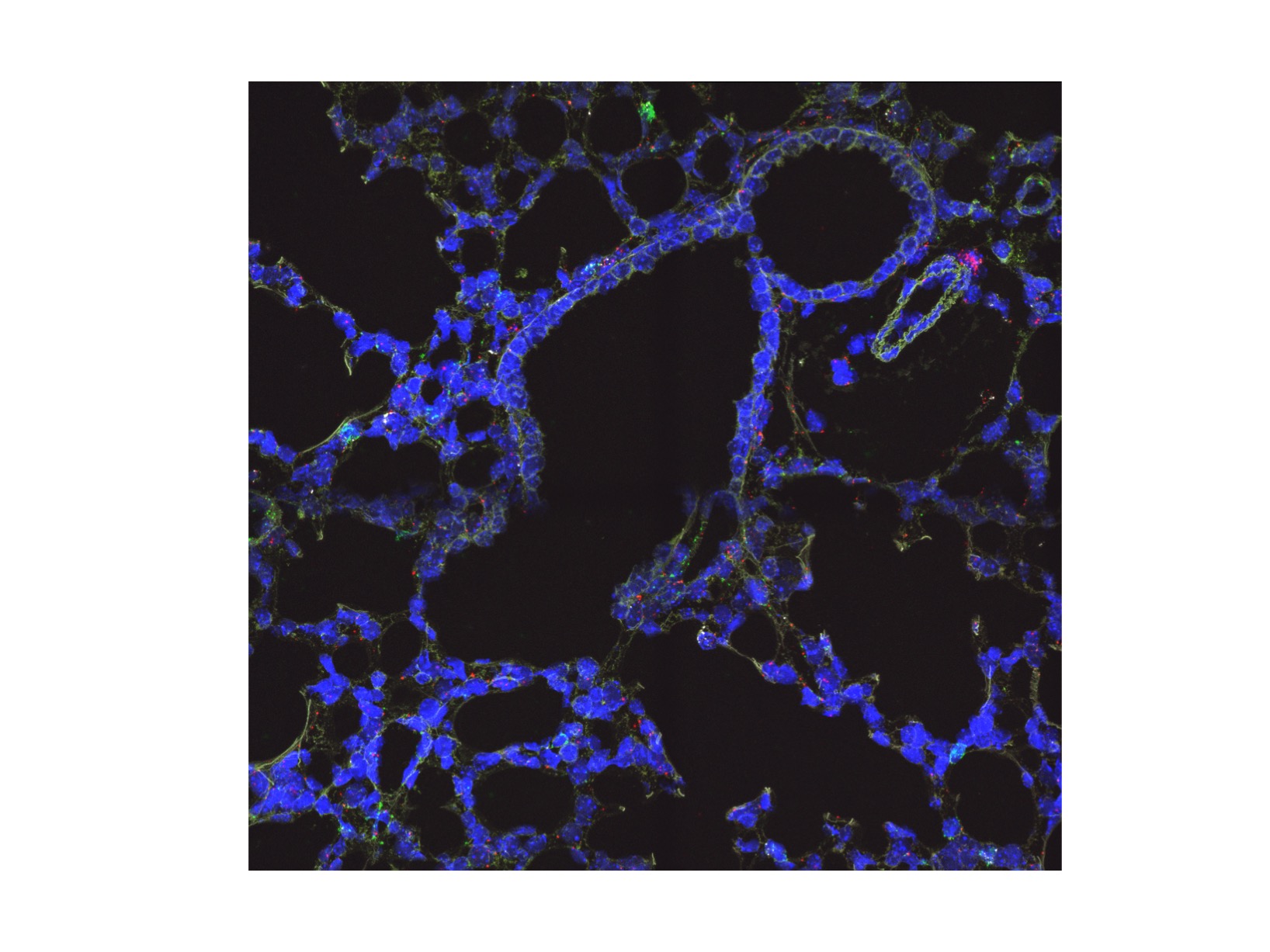

Supplement: Figure 2—source data 1. — This zip archive contains all the fluorescent micrographs used for the quantitative analysis shown in Fig. blank. The individual files are named with the timepoint (for figures containing more than one timepoint), the gene detected by FISH, followed by the color of the label for the gene with ‘G’ for green, ‘R’ for red, ‘W’ for white, and ‘Y’ for yellow. [file elife-56890-fig2-data1.zip › Source FIles for Dab2 and Plac8 JPEG/Slide3_P7_Cd68W_Dab2R-Plac8G.jpeg]

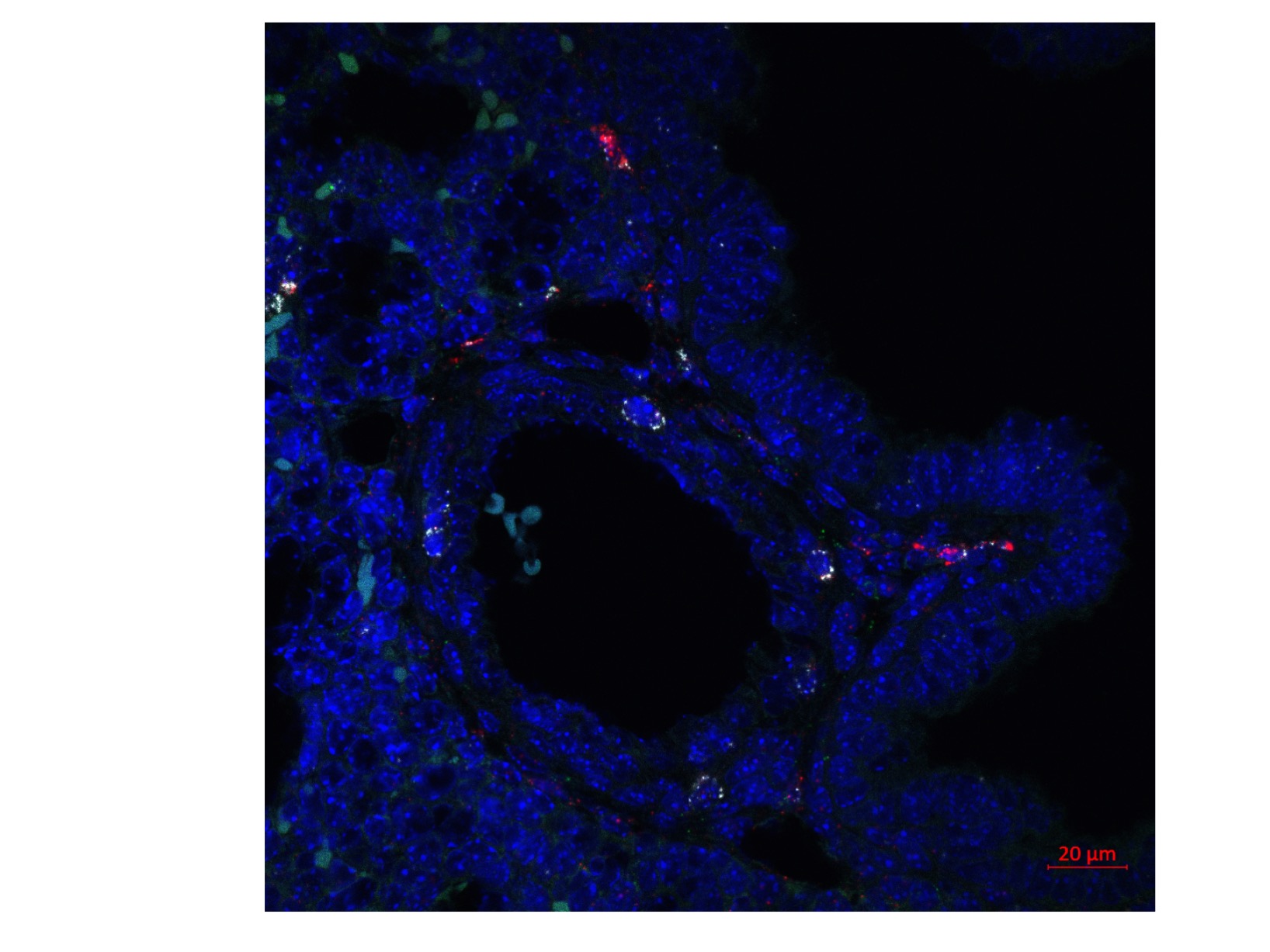

Supplement: Figure 2—source data 1. — This zip archive contains all the fluorescent micrographs used for the quantitative analysis shown in Fig. blank. The individual files are named with the timepoint (for figures containing more than one timepoint), the gene detected by FISH, followed by the color of the label for the gene with ‘G’ for green, ‘R’ for red, ‘W’ for white, and ‘Y’ for yellow. [file elife-56890-fig2-data1.zip › Source FIles for Dab2 and Plac8 JPEG/Slide4_E18.5_Cd68W_Dab2R_Plac8G.jpeg]

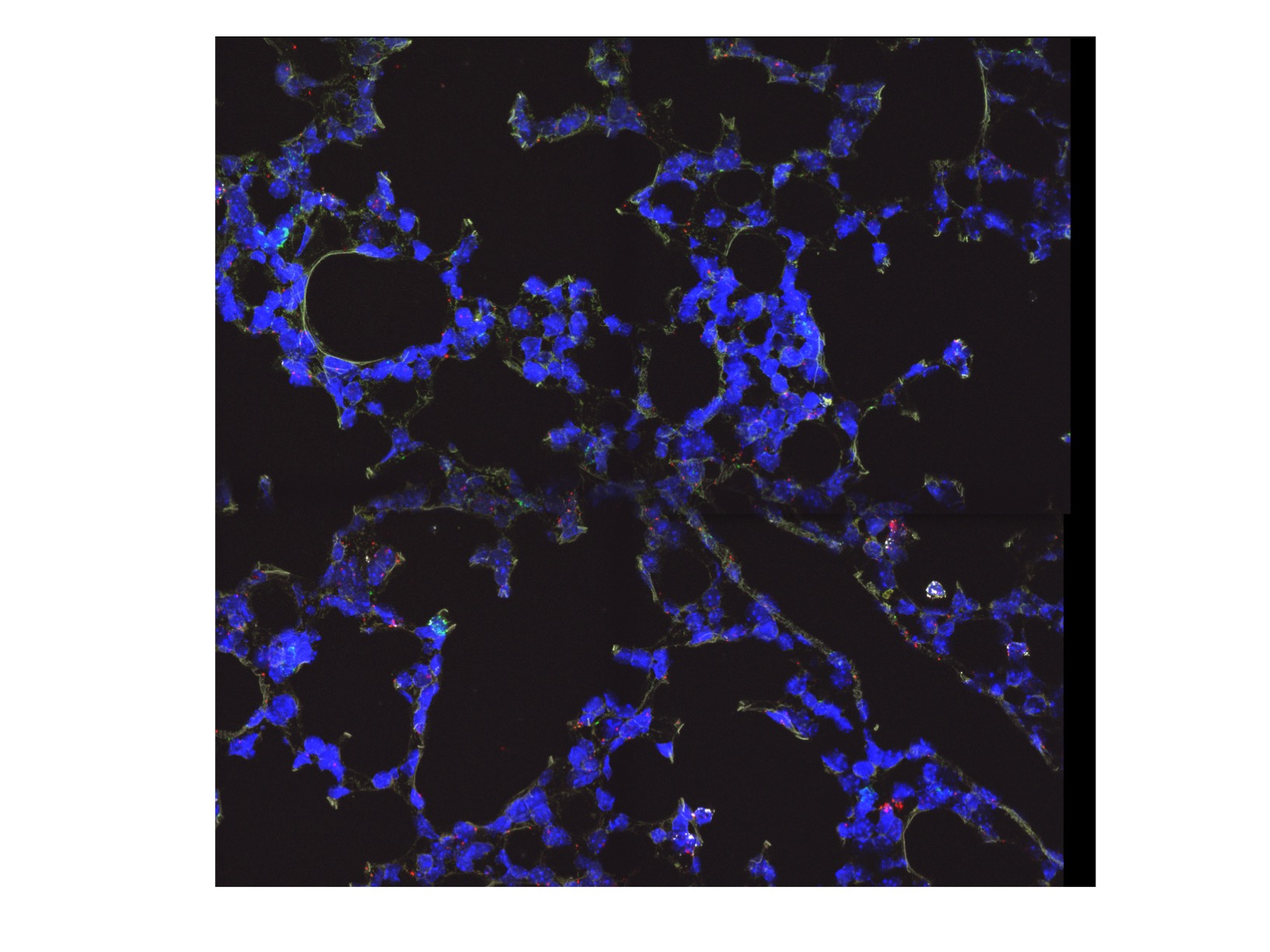

Supplement: Figure 2—source data 1. — This zip archive contains all the fluorescent micrographs used for the quantitative analysis shown in Fig. blank. The individual files are named with the timepoint (for figures containing more than one timepoint), the gene detected by FISH, followed by the color of the label for the gene with ‘G’ for green, ‘R’ for red, ‘W’ for white, and ‘Y’ for yellow. [file elife-56890-fig2-data1.zip › Source FIles for Dab2 and Plac8 JPEG/Slide4_P7_Cd68W_Dab2R-Plac8G.jpeg]

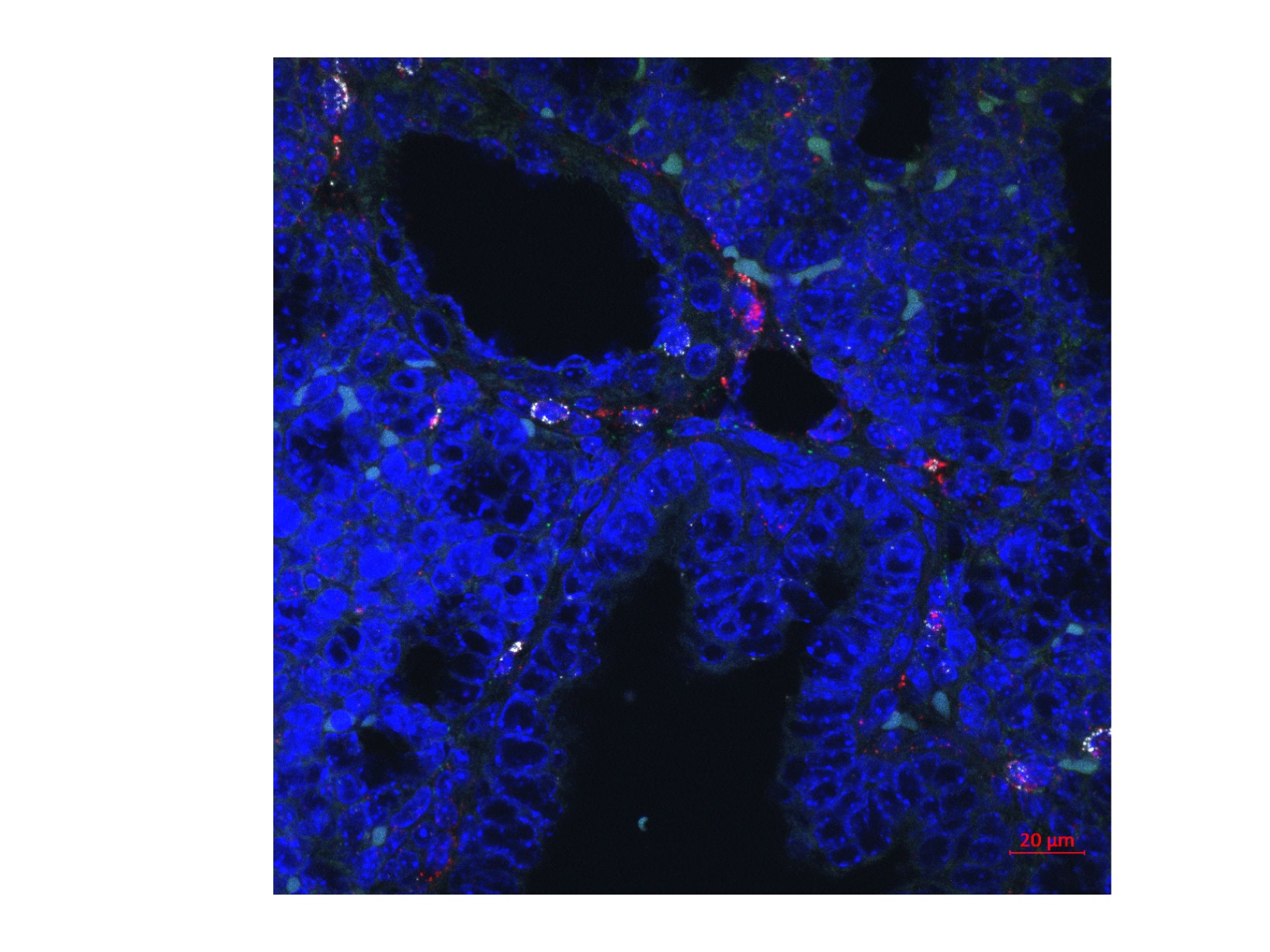

Supplement: Figure 2—source data 1. — This zip archive contains all the fluorescent micrographs used for the quantitative analysis shown in Fig. blank. The individual files are named with the timepoint (for figures containing more than one timepoint), the gene detected by FISH, followed by the color of the label for the gene with ‘G’ for green, ‘R’ for red, ‘W’ for white, and ‘Y’ for yellow. [file elife-56890-fig2-data1.zip › Source FIles for Dab2 and Plac8 JPEG/Slide5_E18.5_Cd68W_Dab2R_Plac8G.jpeg]

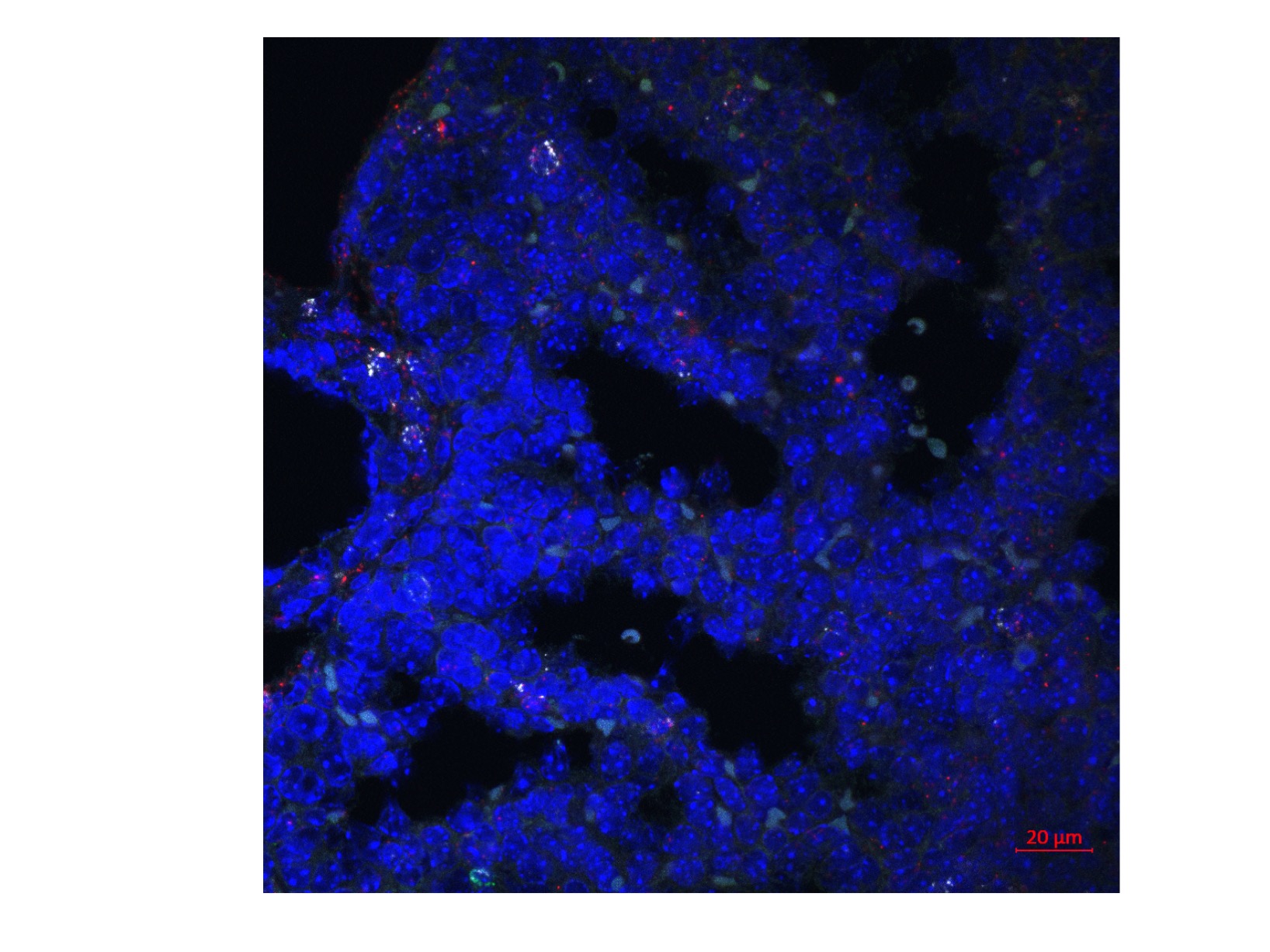

Supplement: Figure 2—source data 1. — This zip archive contains all the fluorescent micrographs used for the quantitative analysis shown in Fig. blank. The individual files are named with the timepoint (for figures containing more than one timepoint), the gene detected by FISH, followed by the color of the label for the gene with ‘G’ for green, ‘R’ for red, ‘W’ for white, and ‘Y’ for yellow. [file elife-56890-fig2-data1.zip › Source FIles for Dab2 and Plac8 JPEG/Slide6_E18.5_Cd68W_Dab2R_Plac8G.jpeg]

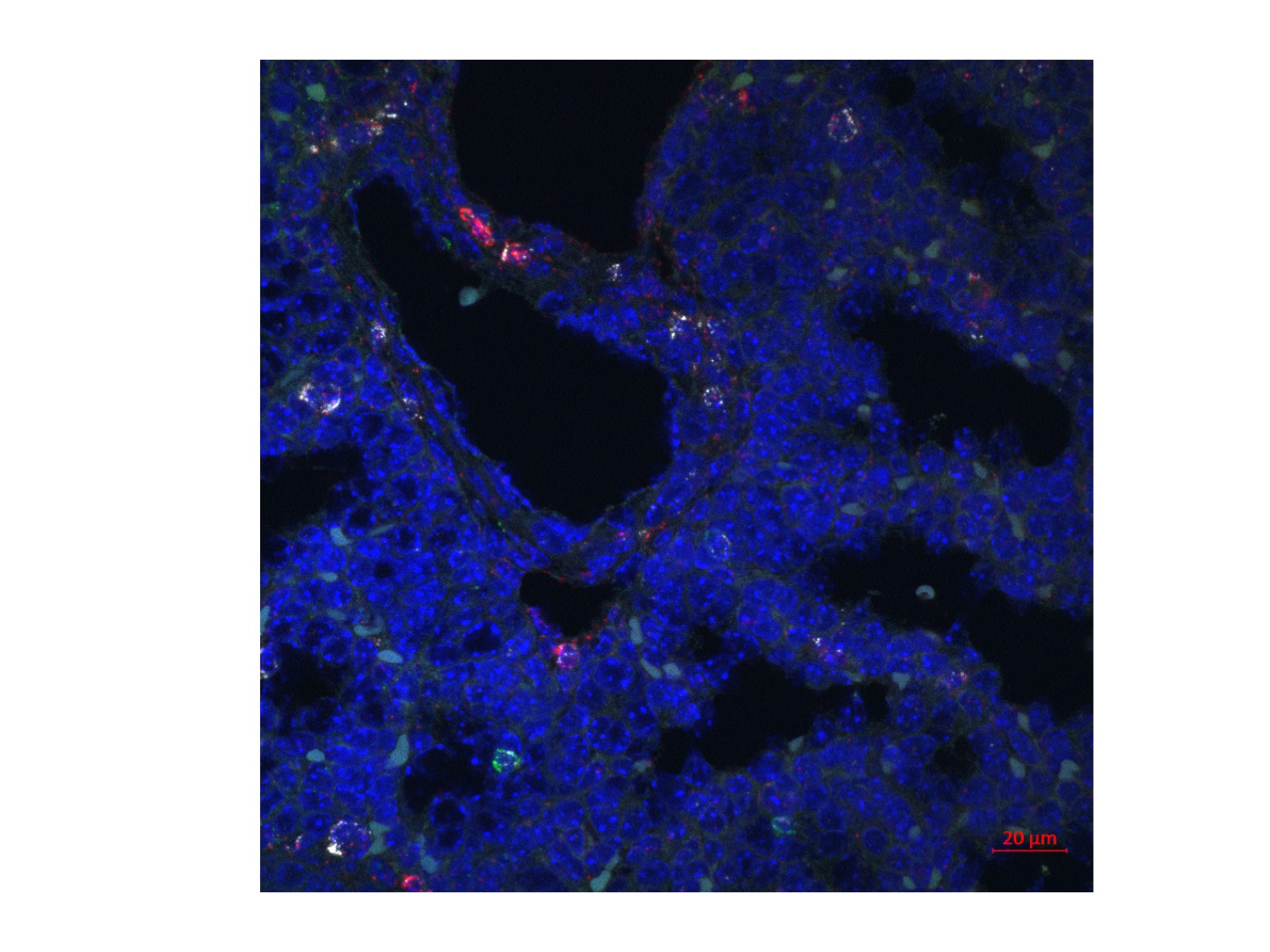

Supplement: Figure 2—source data 1. — This zip archive contains all the fluorescent micrographs used for the quantitative analysis shown in Fig. blank. The individual files are named with the timepoint (for figures containing more than one timepoint), the gene detected by FISH, followed by the color of the label for the gene with ‘G’ for green, ‘R’ for red, ‘W’ for white, and ‘Y’ for yellow. [file elife-56890-fig2-data1.zip › Source FIles for Dab2 and Plac8 JPEG/Slide7_E18.5_Cd68W_Dab2R_Plac8G.jpeg]

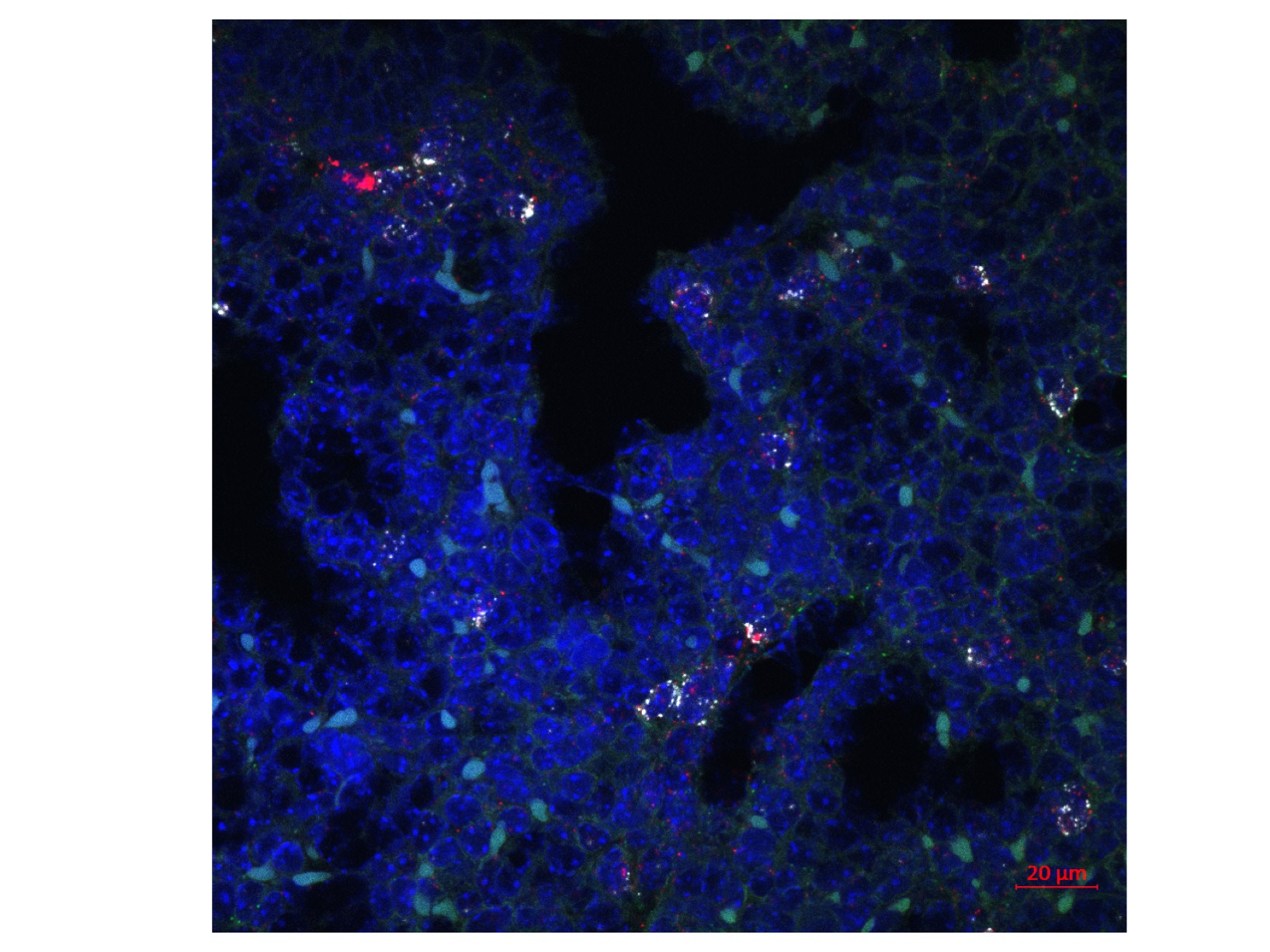

Supplement: Figure 2—source data 1. — This zip archive contains all the fluorescent micrographs used for the quantitative analysis shown in Fig. blank. The individual files are named with the timepoint (for figures containing more than one timepoint), the gene detected by FISH, followed by the color of the label for the gene with ‘G’ for green, ‘R’ for red, ‘W’ for white, and ‘Y’ for yellow. [file elife-56890-fig2-data1.zip › Source FIles for Dab2 and Plac8 JPEG/Slide8_E18.5_Cd68W_Dab2R_Plac8G.jpeg]

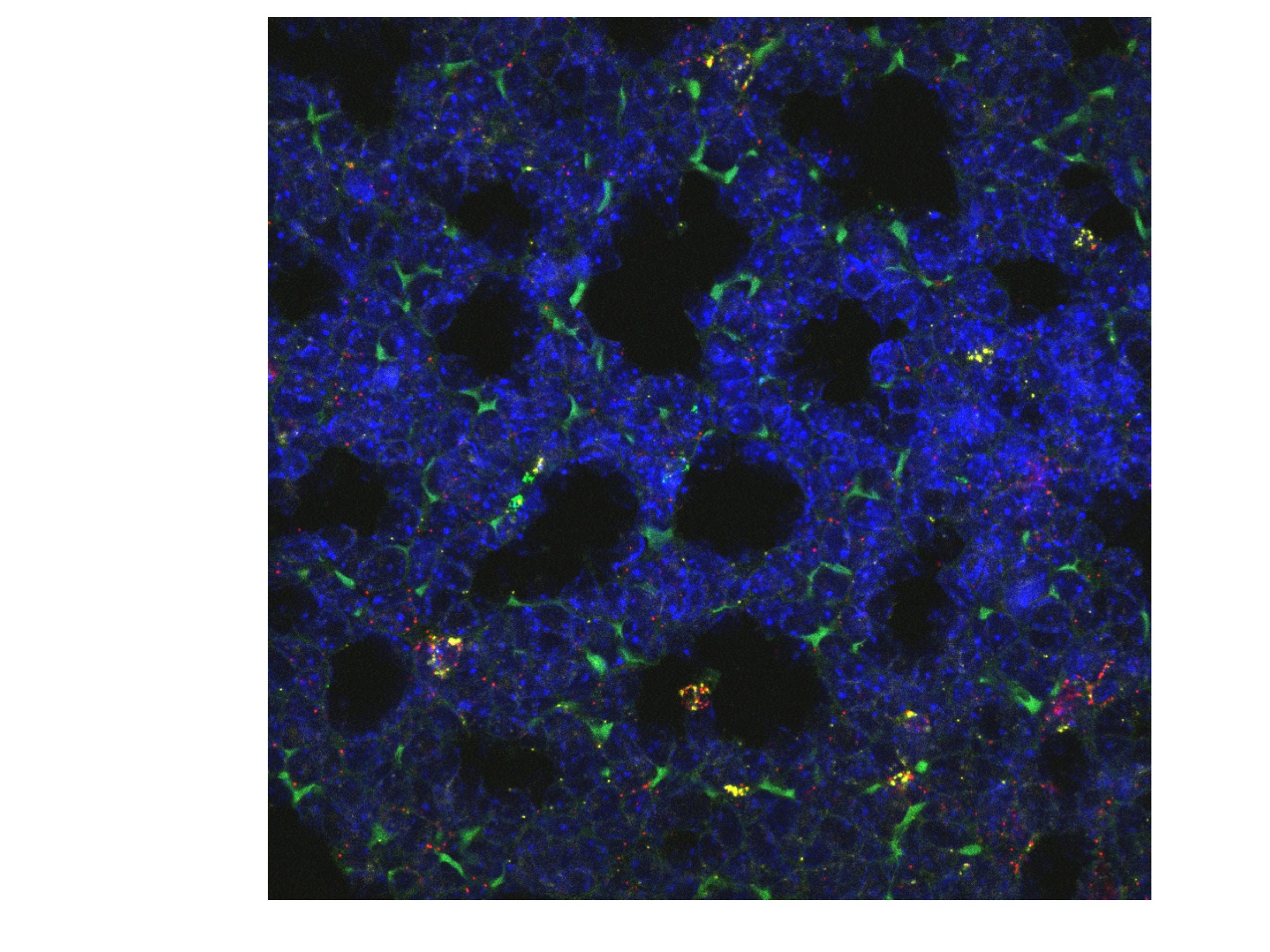

Supplement: Figure 2—source data 1. — This zip archive contains all the fluorescent micrographs used for the quantitative analysis shown in Fig. blank. The individual files are named with the timepoint (for figures containing more than one timepoint), the gene detected by FISH, followed by the color of the label for the gene with ‘G’ for green, ‘R’ for red, ‘W’ for white, and ‘Y’ for yellow. [file elife-56890-fig2-data1.zip › Source FIles for Dab2 and Plac8 JPEG/Slide9_E18.5_Cd68Y_Dab2R_Plac8G.jpeg]

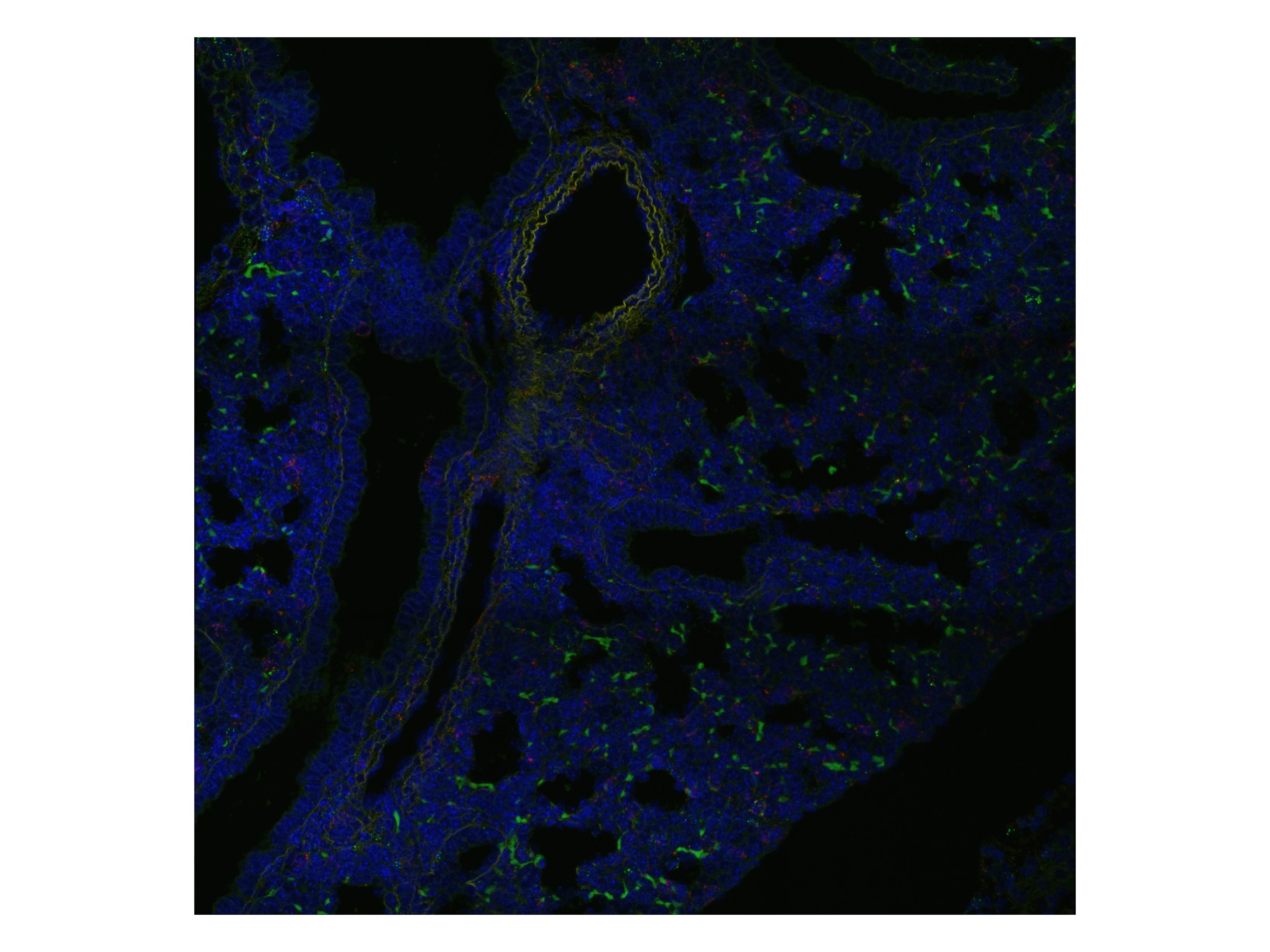

Supplement: Figure 3—source data 1. — This zip archive contains all the fluorescent micrographs used for the quantitative analysis shown in Fig. blank. The individual files are named with the timepoint (for figures containing more than one timepoint), the gene detected by FISH, followed by the color of the label for the gene with ‘G’ for green, ‘R’ for red, ‘W’ for white, and ‘Y’ for yellow. [file elife-56890-fig3-data1.zip › Source files Stitch and Tile to Upload/Slide10_Cd68G_Mki67R.jpeg]

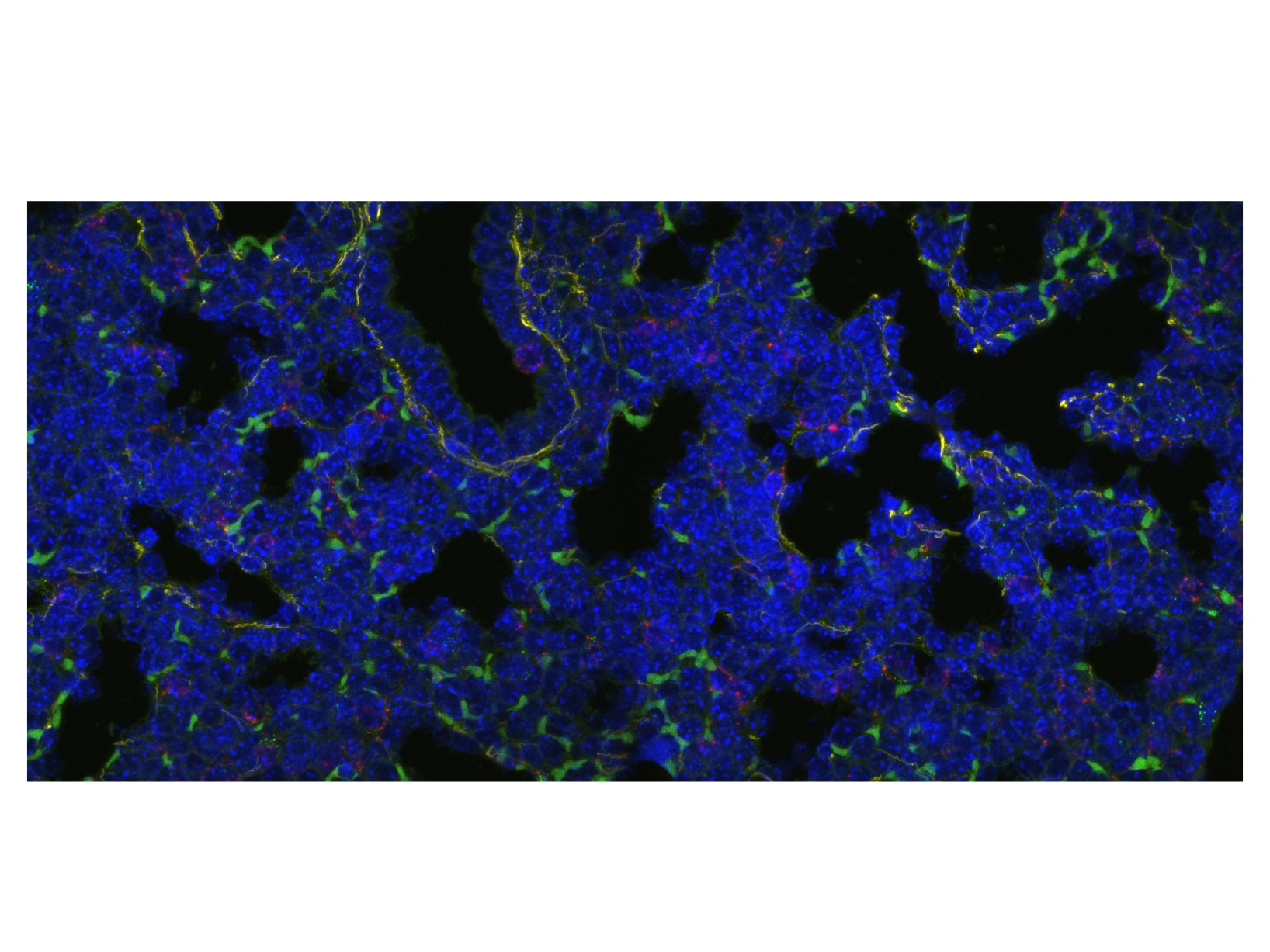

Supplement: Figure 3—source data 1. — This zip archive contains all the fluorescent micrographs used for the quantitative analysis shown in Fig. blank. The individual files are named with the timepoint (for figures containing more than one timepoint), the gene detected by FISH, followed by the color of the label for the gene with ‘G’ for green, ‘R’ for red, ‘W’ for white, and ‘Y’ for yellow. [file elife-56890-fig3-data1.zip › Source files Stitch and Tile to Upload/Slide11_Cd68G_Mki67R.jpeg]

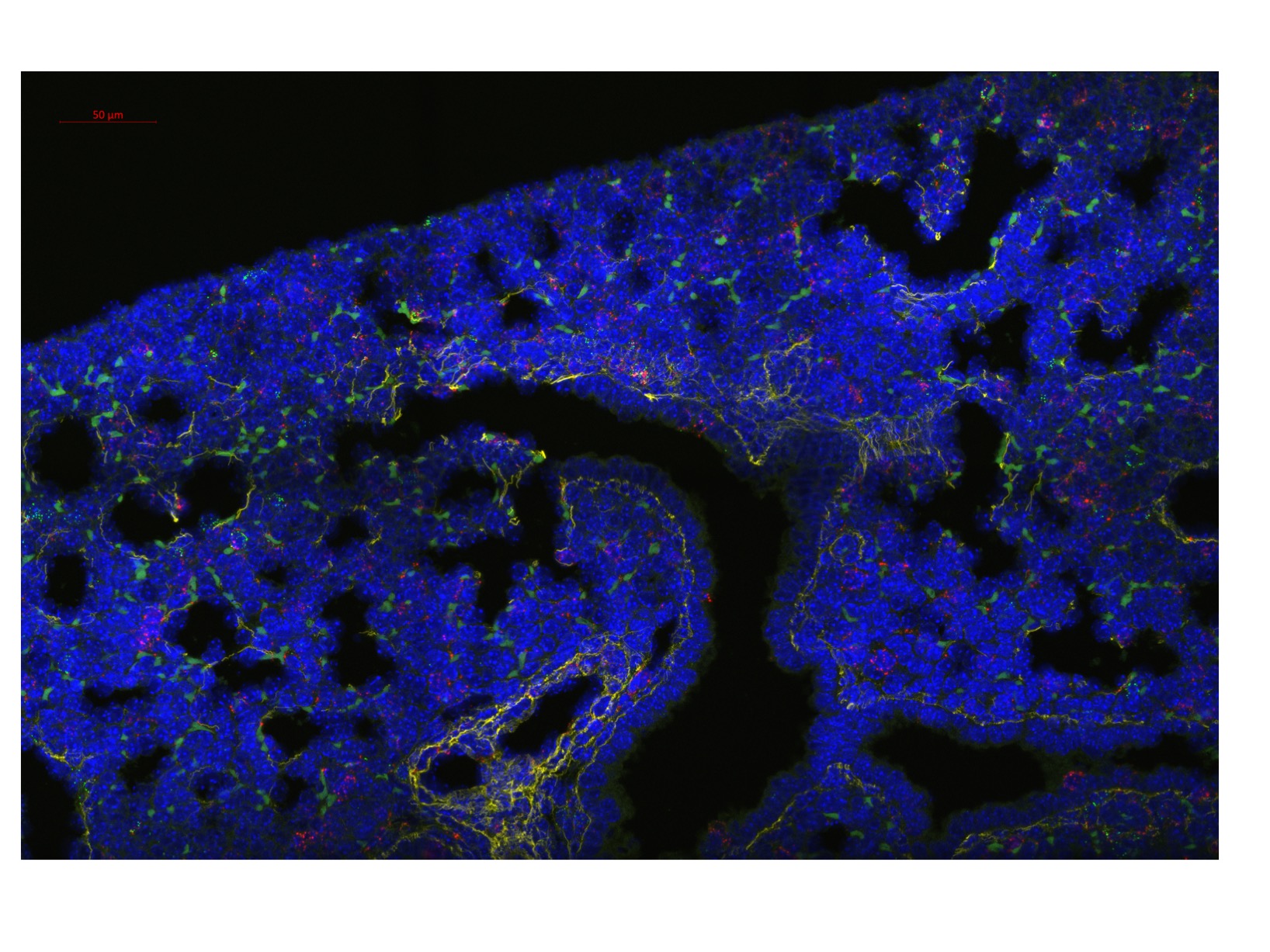

Supplement: Figure 3—source data 1. — This zip archive contains all the fluorescent micrographs used for the quantitative analysis shown in Fig. blank. The individual files are named with the timepoint (for figures containing more than one timepoint), the gene detected by FISH, followed by the color of the label for the gene with ‘G’ for green, ‘R’ for red, ‘W’ for white, and ‘Y’ for yellow. [file elife-56890-fig3-data1.zip › Source files Stitch and Tile to Upload/Slide12_Cd68G_Mki67R.jpeg]

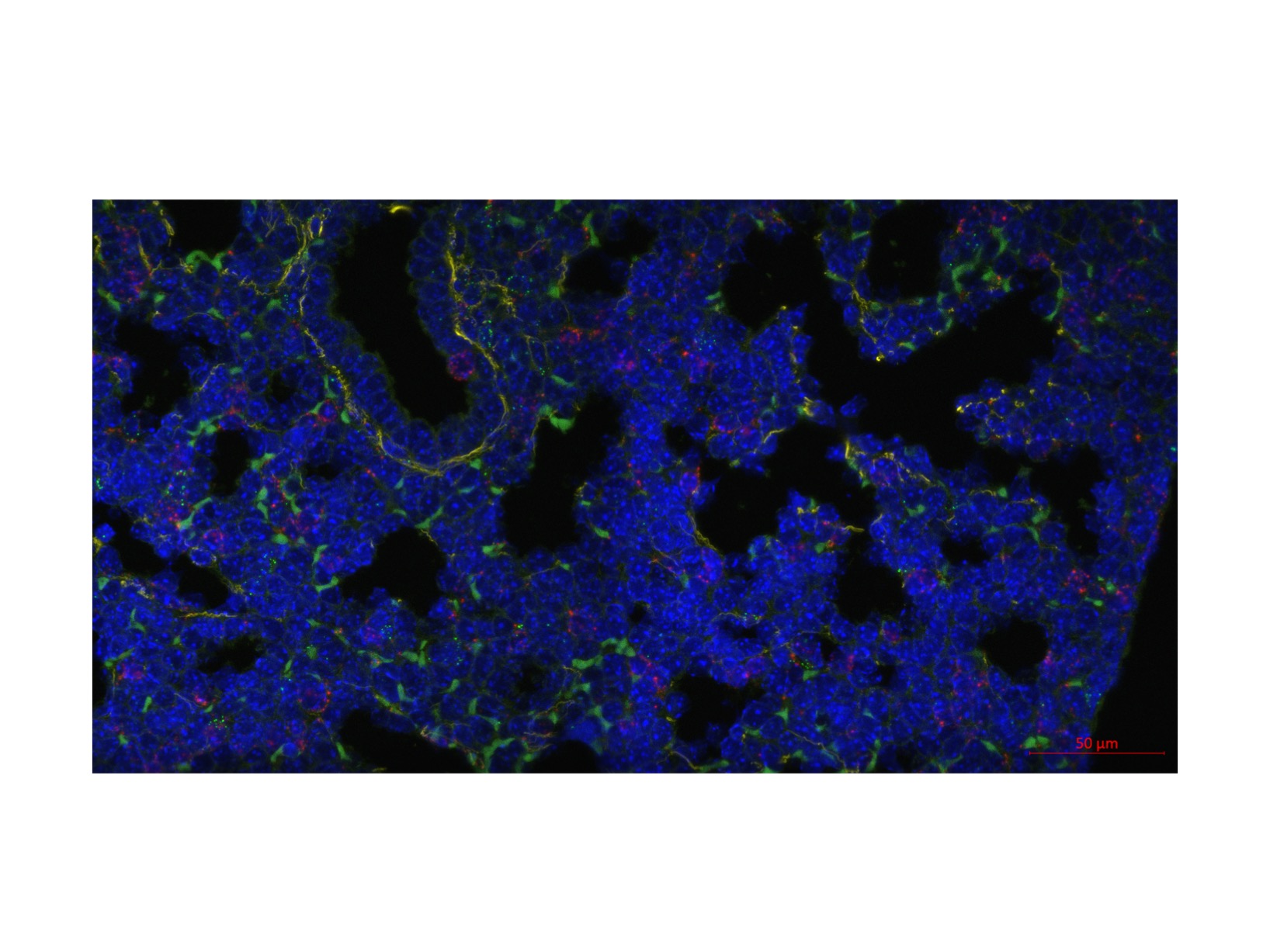

Supplement: Figure 3—source data 1. — This zip archive contains all the fluorescent micrographs used for the quantitative analysis shown in Fig. blank. The individual files are named with the timepoint (for figures containing more than one timepoint), the gene detected by FISH, followed by the color of the label for the gene with ‘G’ for green, ‘R’ for red, ‘W’ for white, and ‘Y’ for yellow. [file elife-56890-fig3-data1.zip › Source files Stitch and Tile to Upload/Slide13_Cd68G_Mki67R.jpeg]

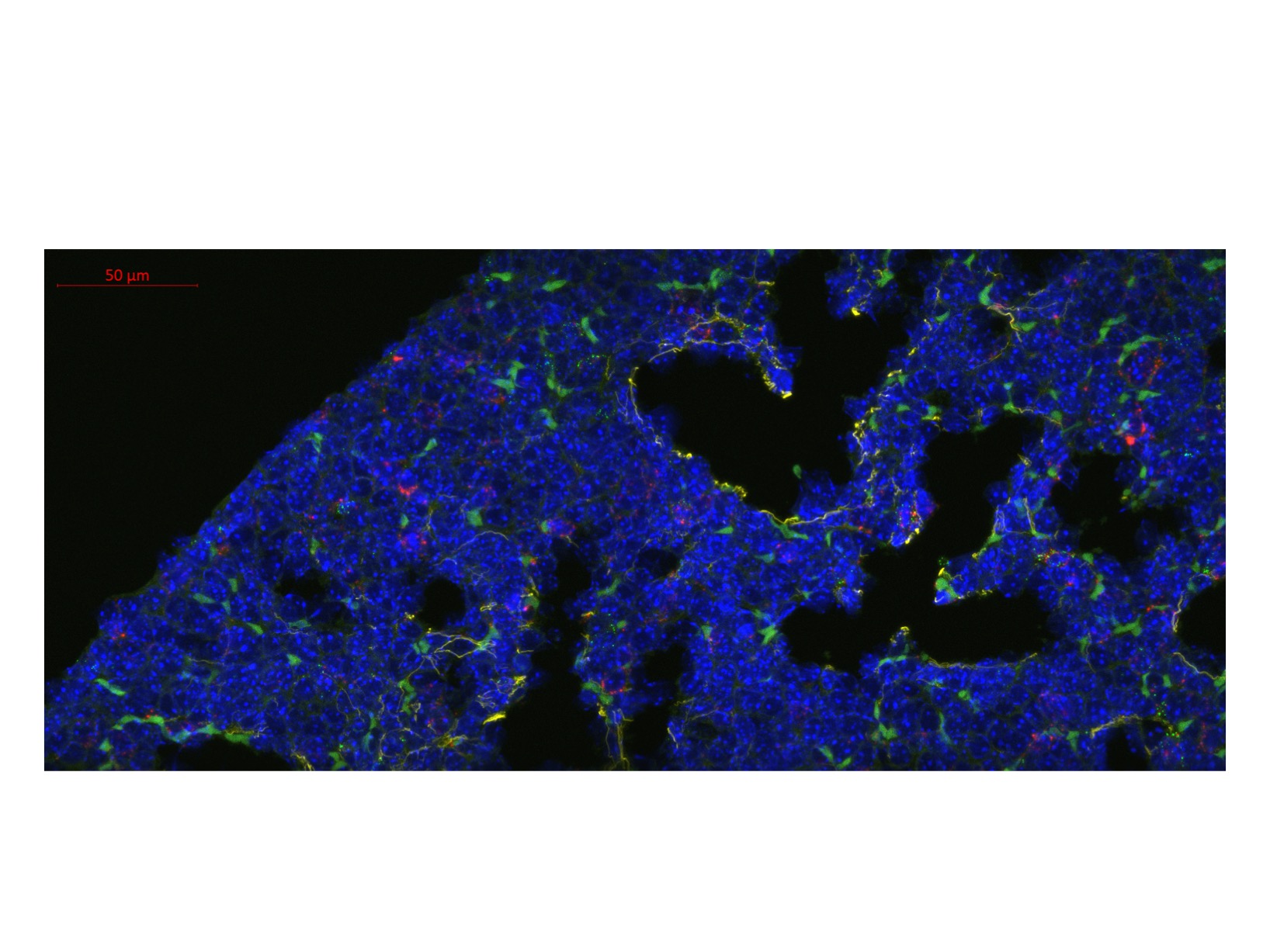

Supplement: Figure 3—source data 1. — This zip archive contains all the fluorescent micrographs used for the quantitative analysis shown in Fig. blank. The individual files are named with the timepoint (for figures containing more than one timepoint), the gene detected by FISH, followed by the color of the label for the gene with ‘G’ for green, ‘R’ for red, ‘W’ for white, and ‘Y’ for yellow. [file elife-56890-fig3-data1.zip › Source files Stitch and Tile to Upload/Slide14_Cd68G_Mki67R.jpeg]

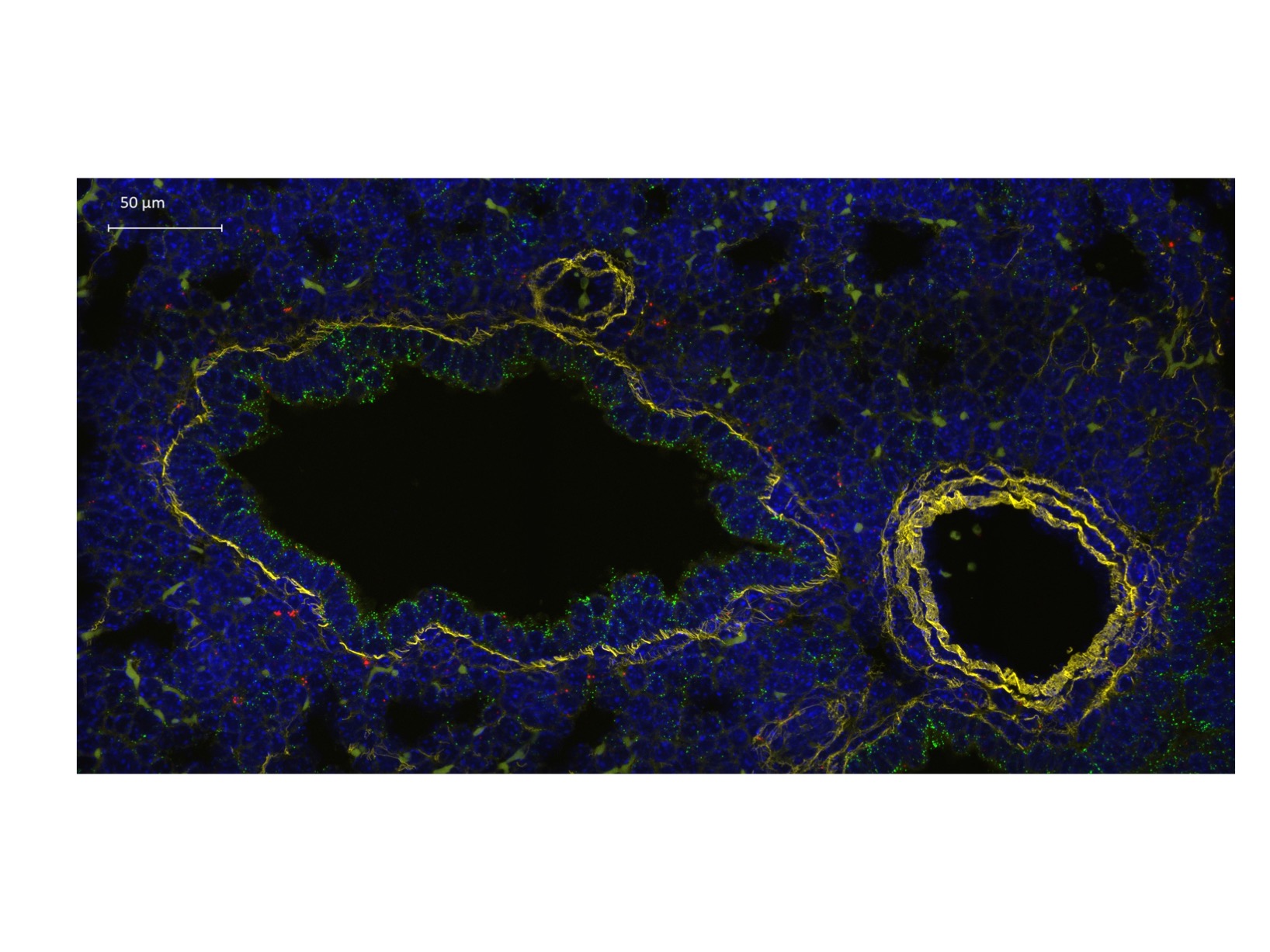

Supplement: Figure 3—source data 1. — This zip archive contains all the fluorescent micrographs used for the quantitative analysis shown in Fig. blank. The individual files are named with the timepoint (for figures containing more than one timepoint), the gene detected by FISH, followed by the color of the label for the gene with ‘G’ for green, ‘R’ for red, ‘W’ for white, and ‘Y’ for yellow. [file elife-56890-fig3-data1.zip › Source files Stitch and Tile to Upload/Slide1_Cd68R_EpcamG.jpeg]

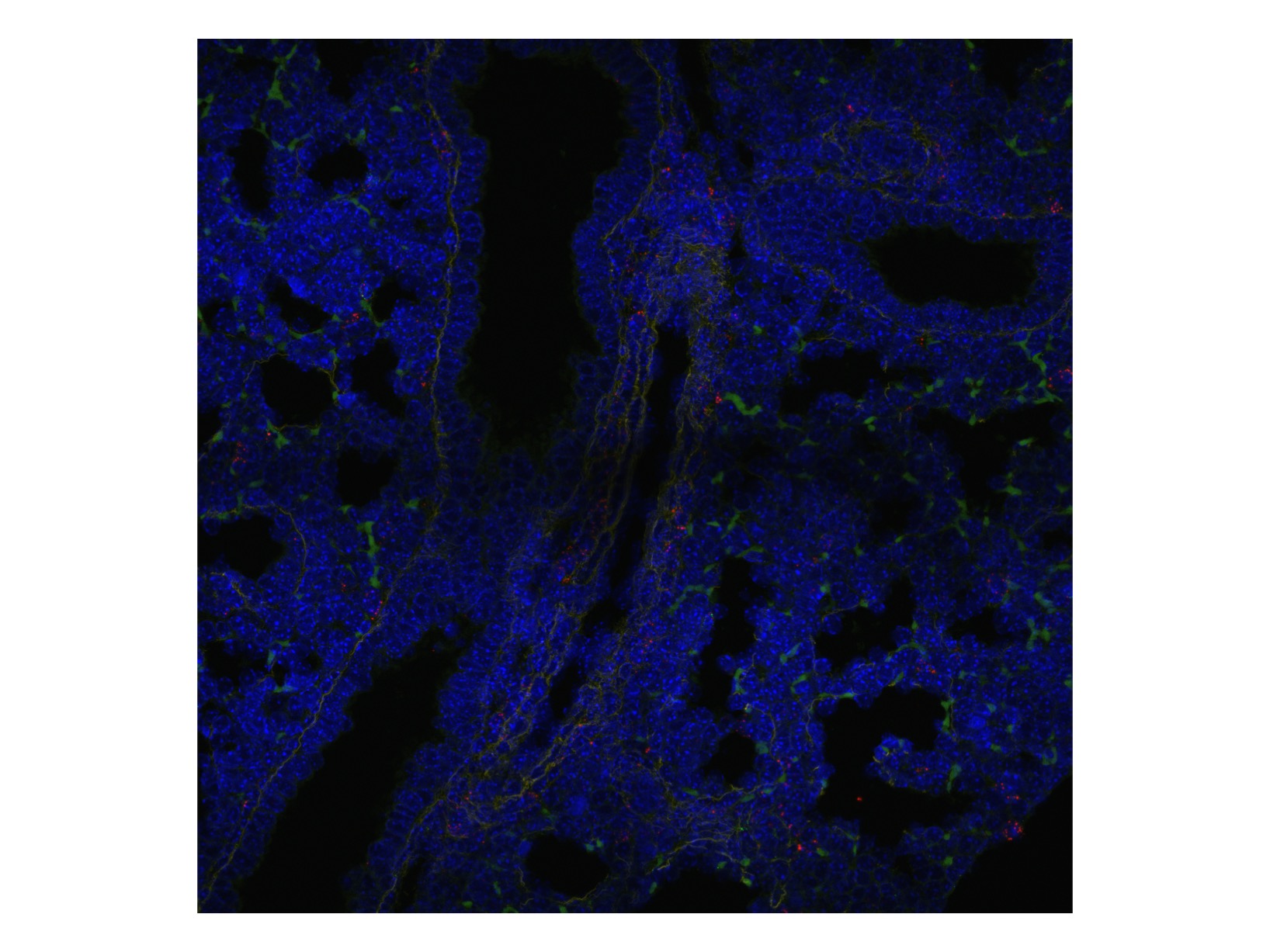

Supplement: Figure 3—source data 1. — This zip archive contains all the fluorescent micrographs used for the quantitative analysis shown in Fig. blank. The individual files are named with the timepoint (for figures containing more than one timepoint), the gene detected by FISH, followed by the color of the label for the gene with ‘G’ for green, ‘R’ for red, ‘W’ for white, and ‘Y’ for yellow. [file elife-56890-fig3-data1.zip › Source files Stitch and Tile to Upload/Slide2_Cd68R_Plac8G.jpeg]

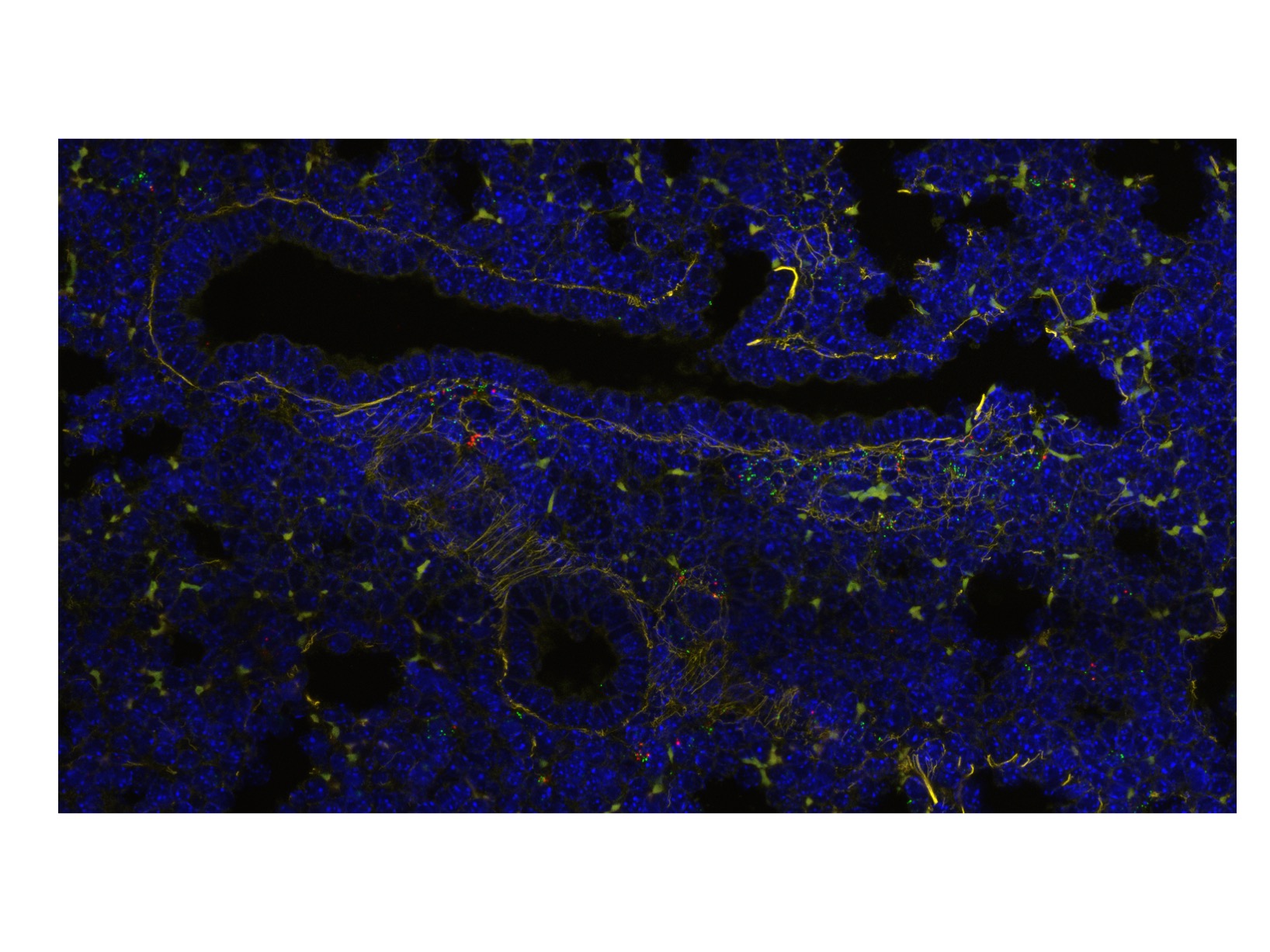

Supplement: Figure 3—source data 1. — This zip archive contains all the fluorescent micrographs used for the quantitative analysis shown in Fig. blank. The individual files are named with the timepoint (for figures containing more than one timepoint), the gene detected by FISH, followed by the color of the label for the gene with ‘G’ for green, ‘R’ for red, ‘W’ for white, and ‘Y’ for yellow. [file elife-56890-fig3-data1.zip › Source files Stitch and Tile to Upload/Slide3_Cd68G_GalR.jpeg]

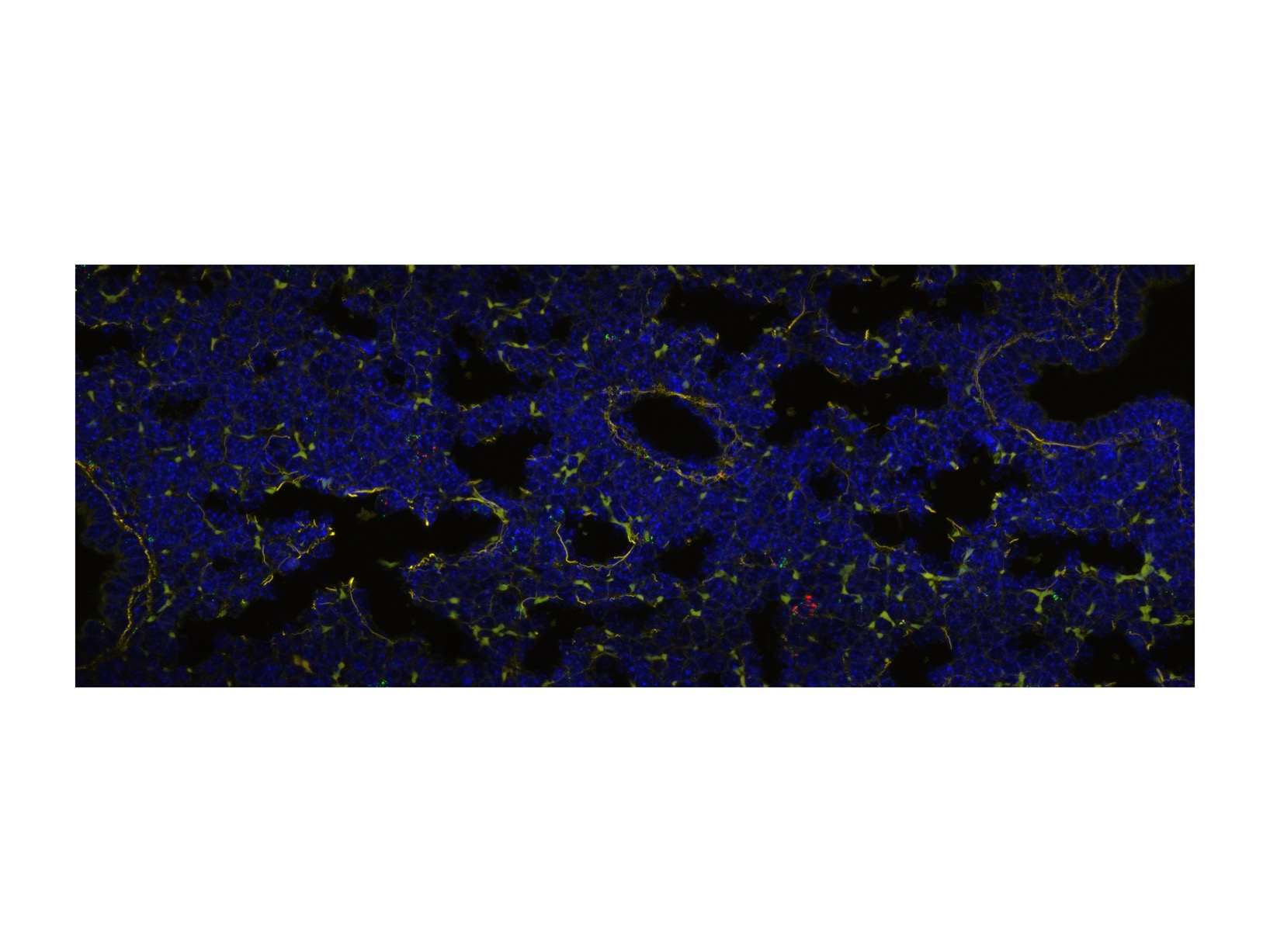

Supplement: Figure 3—source data 1. — This zip archive contains all the fluorescent micrographs used for the quantitative analysis shown in Fig. blank. The individual files are named with the timepoint (for figures containing more than one timepoint), the gene detected by FISH, followed by the color of the label for the gene with ‘G’ for green, ‘R’ for red, ‘W’ for white, and ‘Y’ for yellow. [file elife-56890-fig3-data1.zip › Source files Stitch and Tile to Upload/Slide4_Cd68G_GalR.jpeg]

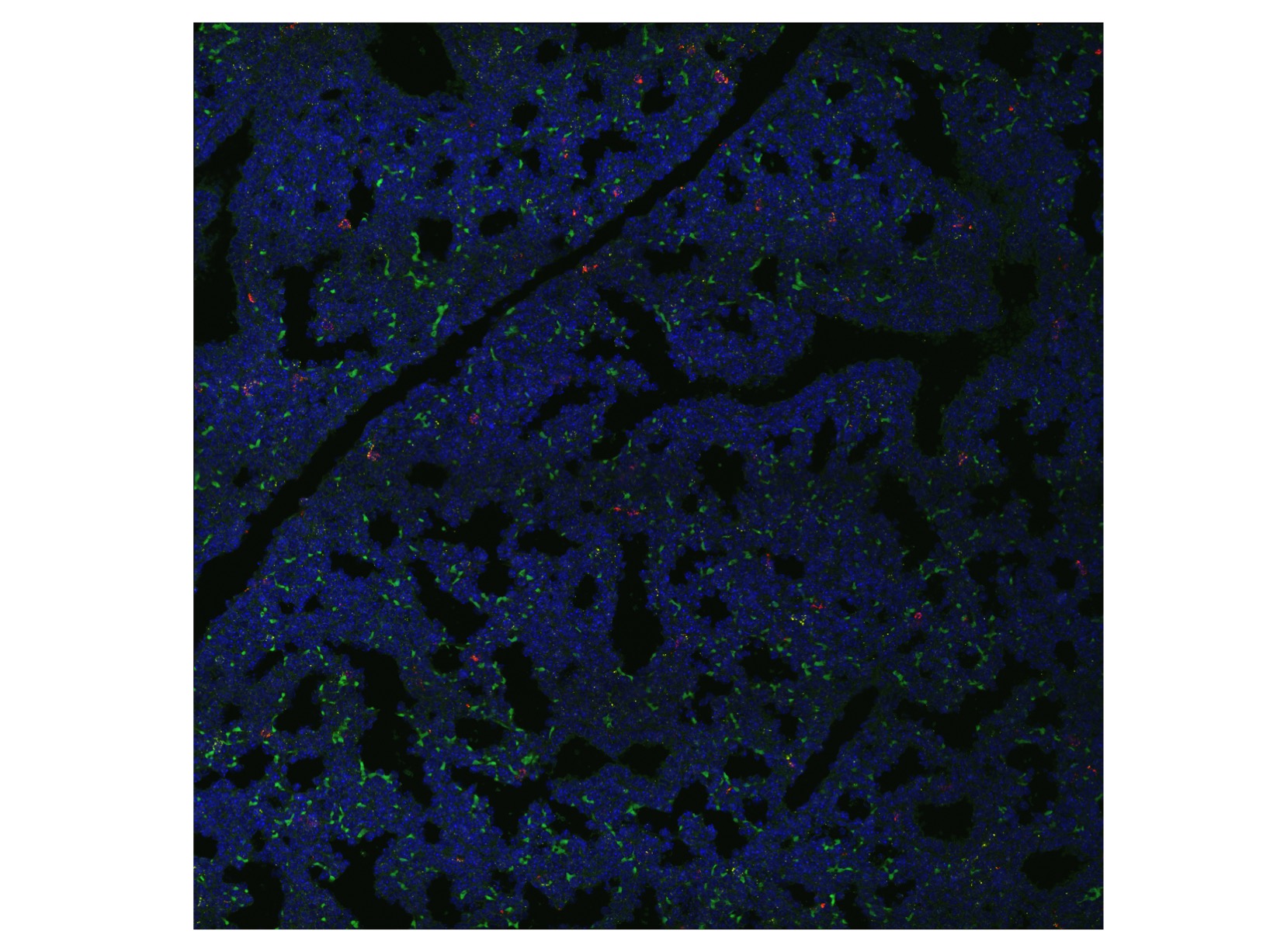

Supplement: Figure 3—source data 1. — This zip archive contains all the fluorescent micrographs used for the quantitative analysis shown in Fig. blank. The individual files are named with the timepoint (for figures containing more than one timepoint), the gene detected by FISH, followed by the color of the label for the gene with ‘G’ for green, ‘R’ for red, ‘W’ for white, and ‘Y’ for yellow. [file elife-56890-fig3-data1.zip › Source files Stitch and Tile to Upload/Slide5_Cd68W_C1qaG_GalR.jpeg]

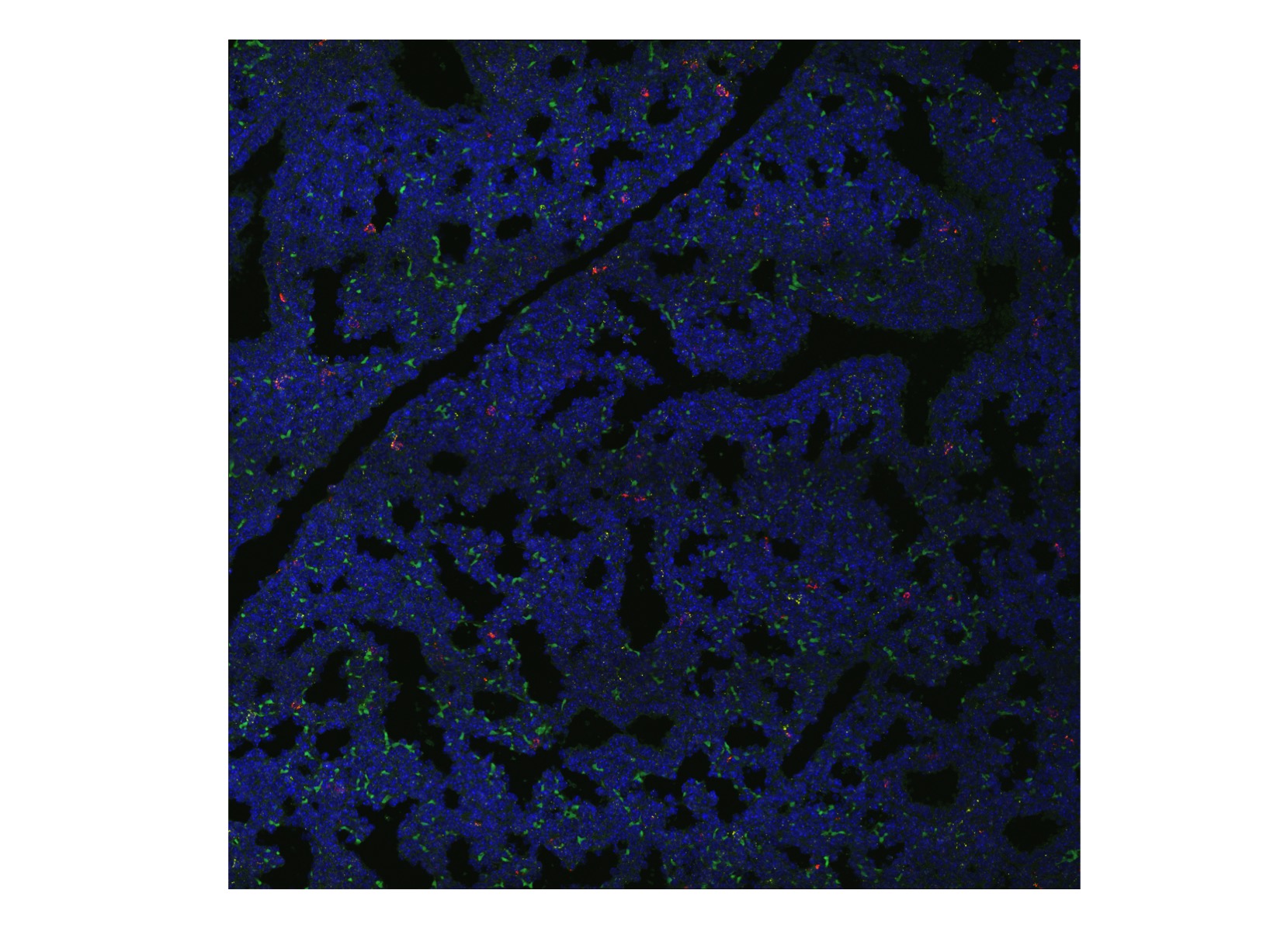

Supplement: Figure 3—source data 1. — This zip archive contains all the fluorescent micrographs used for the quantitative analysis shown in Fig. blank. The individual files are named with the timepoint (for figures containing more than one timepoint), the gene detected by FISH, followed by the color of the label for the gene with ‘G’ for green, ‘R’ for red, ‘W’ for white, and ‘Y’ for yellow. [file elife-56890-fig3-data1.zip › Source files Stitch and Tile to Upload/Slide6_Cd68W_C1qaG_GalR.jpeg]

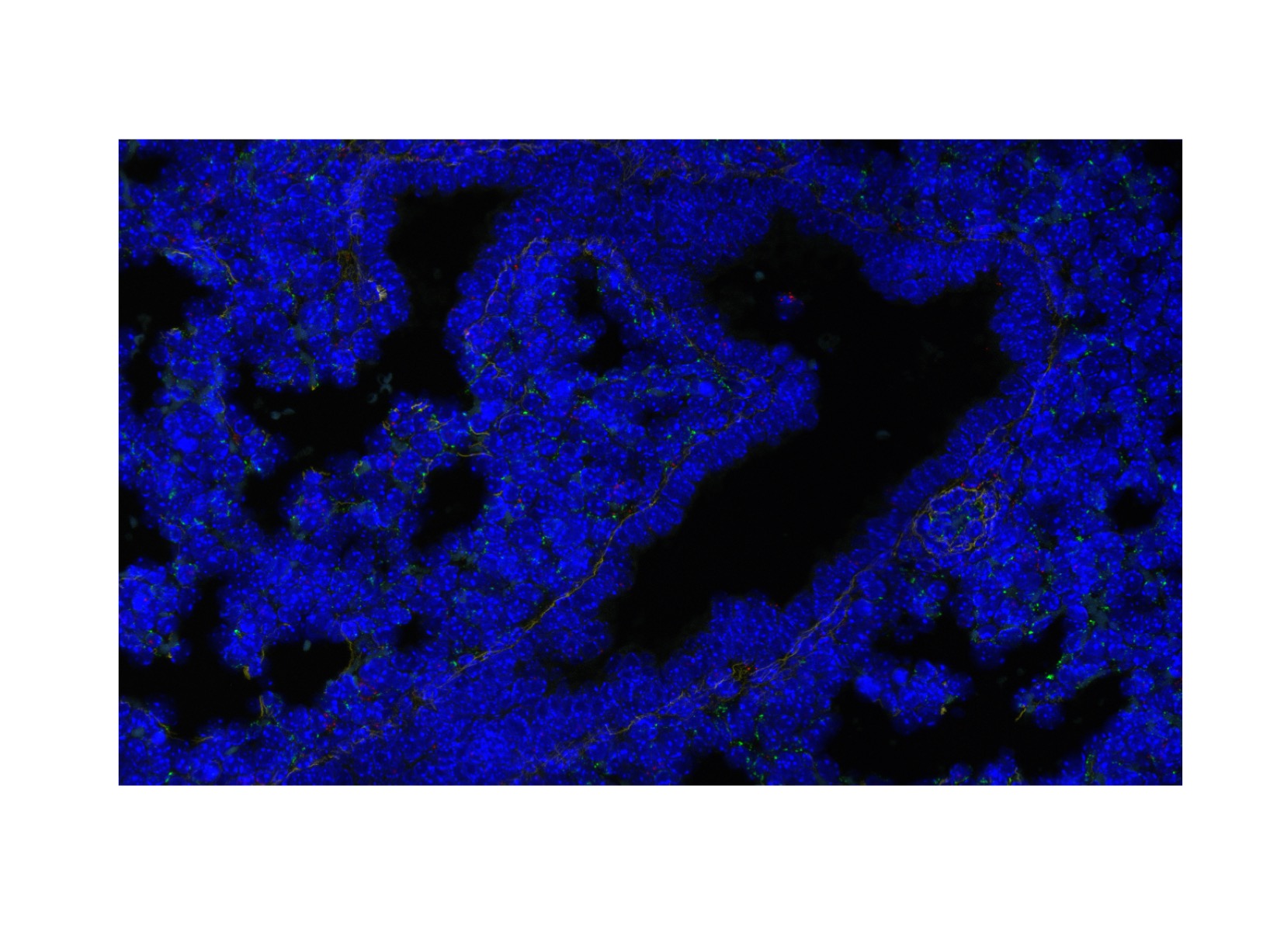

Supplement: Figure 3—source data 1. — This zip archive contains all the fluorescent micrographs used for the quantitative analysis shown in Fig. blank. The individual files are named with the timepoint (for figures containing more than one timepoint), the gene detected by FISH, followed by the color of the label for the gene with ‘G’ for green, ‘R’ for red, ‘W’ for white, and ‘Y’ for yellow. [file elife-56890-fig3-data1.zip › Source files Stitch and Tile to Upload/Slide7_Cd68R_Cdh5G.jpeg]

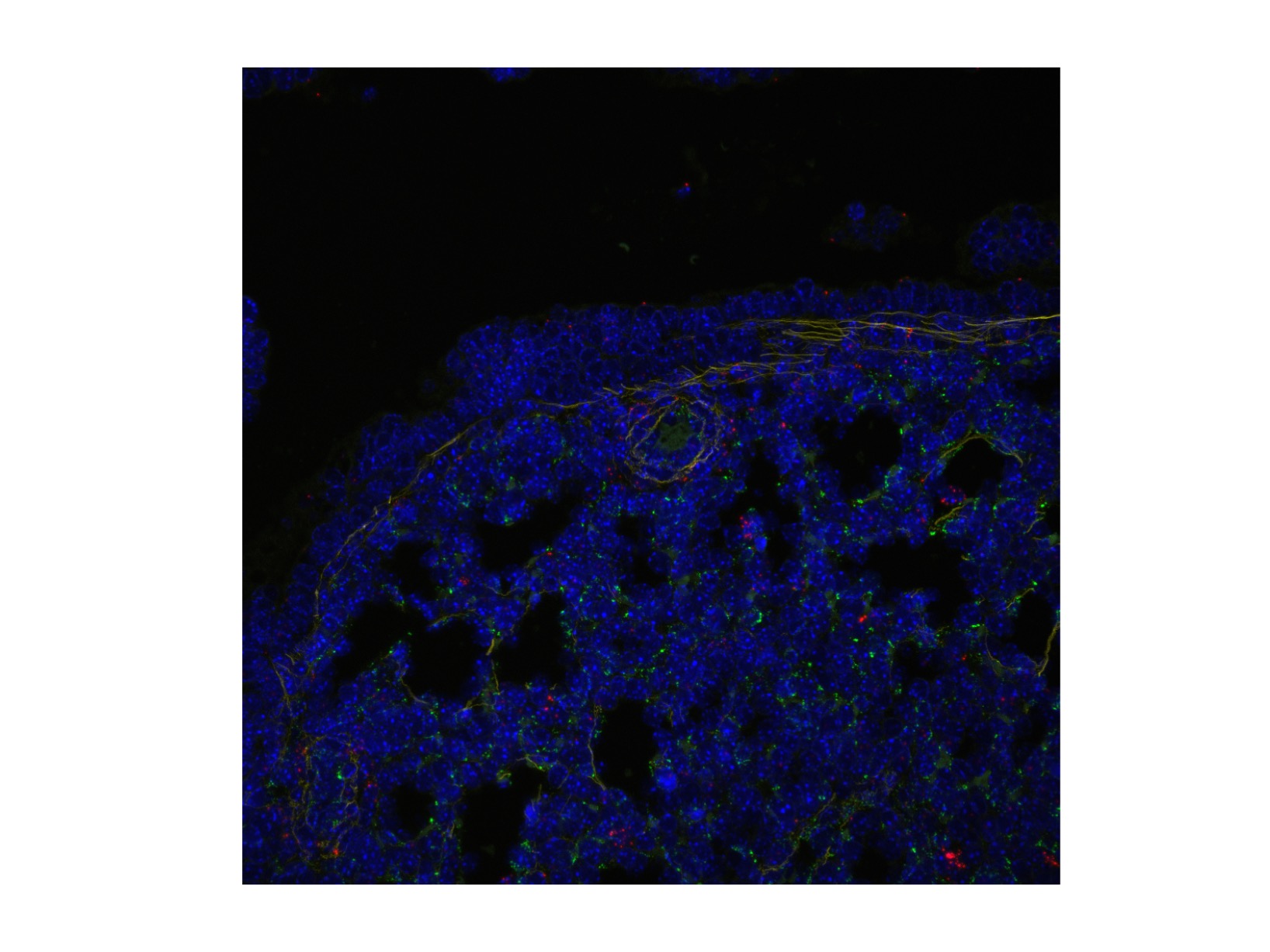

Supplement: Figure 3—source data 1. — This zip archive contains all the fluorescent micrographs used for the quantitative analysis shown in Fig. blank. The individual files are named with the timepoint (for figures containing more than one timepoint), the gene detected by FISH, followed by the color of the label for the gene with ‘G’ for green, ‘R’ for red, ‘W’ for white, and ‘Y’ for yellow. [file elife-56890-fig3-data1.zip › Source files Stitch and Tile to Upload/Slide8_Cd68R_Cdh5G.jpeg]

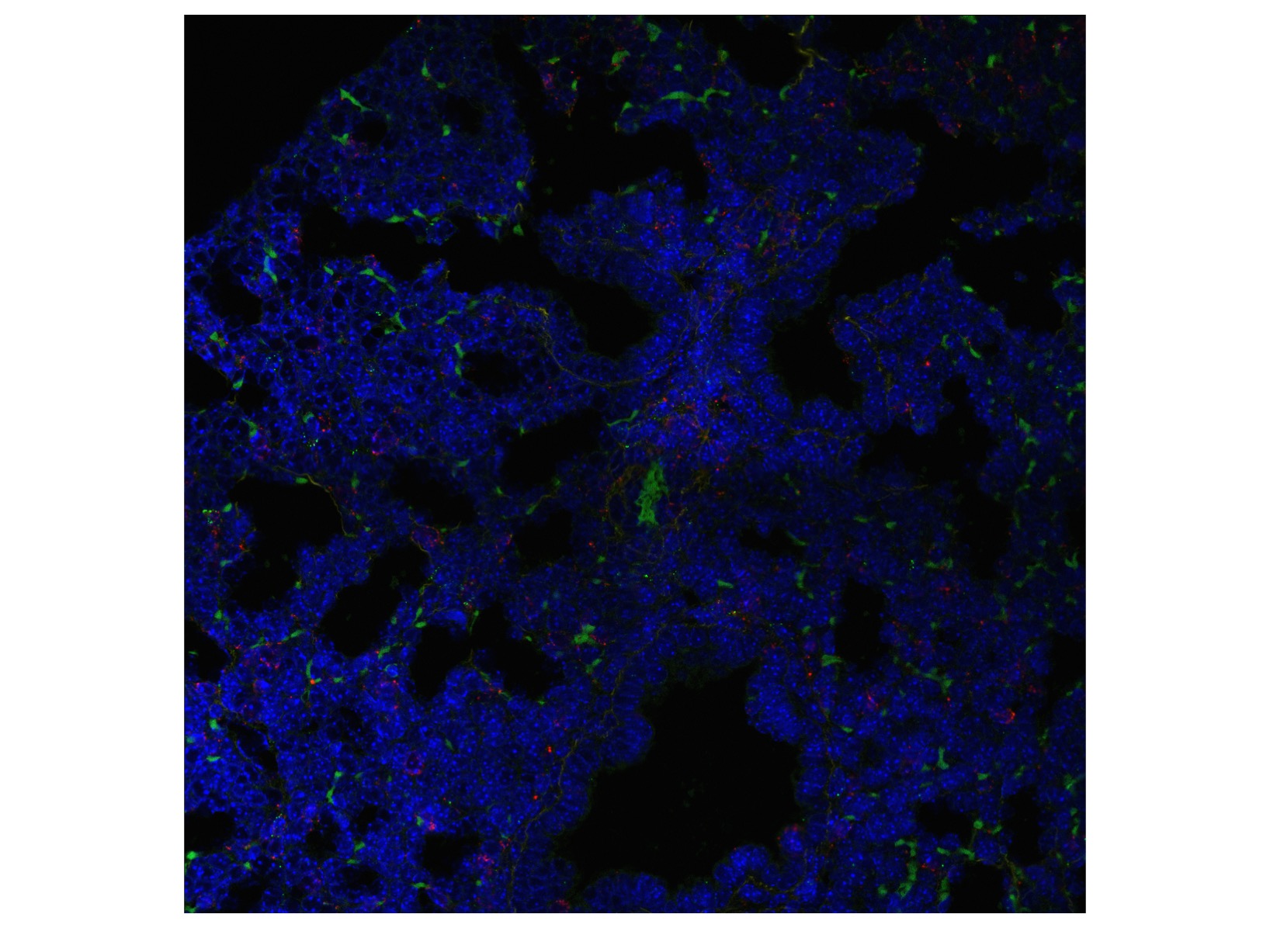

Supplement: Figure 3—source data 1. — This zip archive contains all the fluorescent micrographs used for the quantitative analysis shown in Fig. blank. The individual files are named with the timepoint (for figures containing more than one timepoint), the gene detected by FISH, followed by the color of the label for the gene with ‘G’ for green, ‘R’ for red, ‘W’ for white, and ‘Y’ for yellow. [file elife-56890-fig3-data1.zip › Source files Stitch and Tile to Upload/Slide9_Cd68G_Mki67R.jpeg]

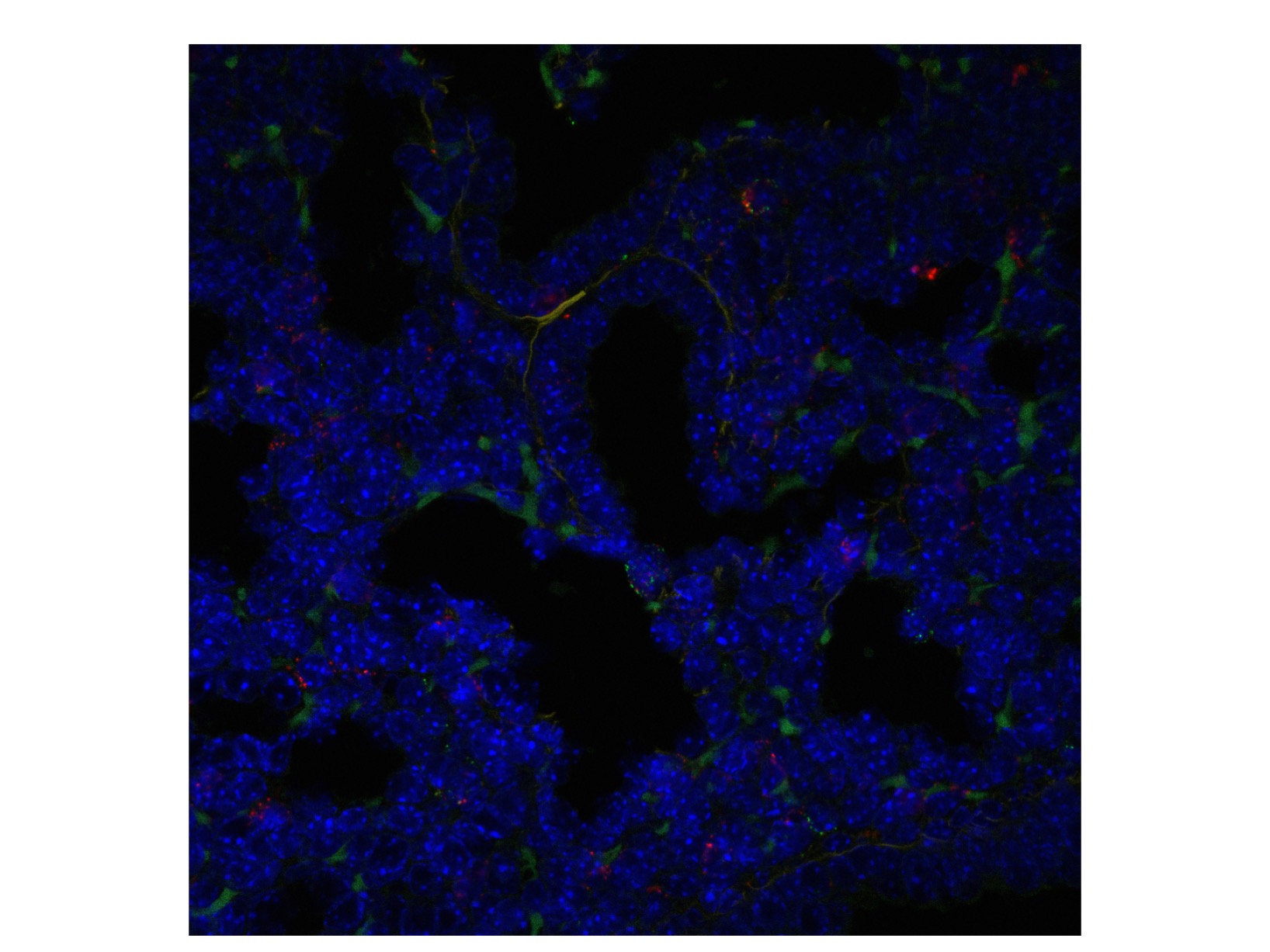

Supplement: Figure 3—source data 2. — This zip archive contains all the fluorescent micrographs used for the quantitative analysis shown in Fig. blank. The individual files are named with the timepoint (for figures containing more than one timepoint), the gene detected by FISH, followed by the color of the label for the gene with ‘G’ for green, ‘R’ for red, ‘W’ for white, and ‘Y’ for yellow. [file elife-56890-fig3-data2.zip › Proliferating macrophages source Files JPEG/Slide10_E18.5_Cd68G_Mki67R.jpeg]

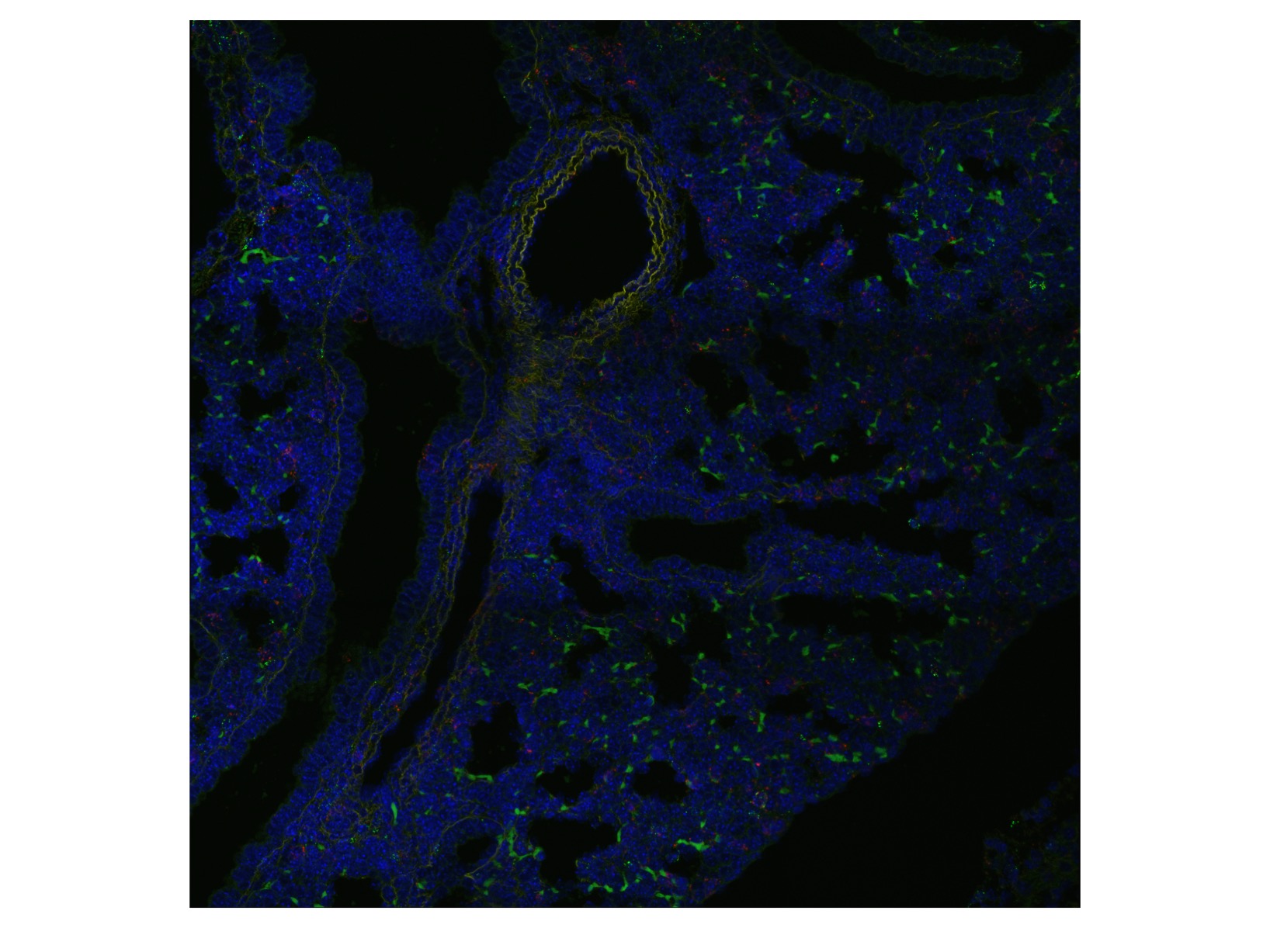

Supplement: Figure 3—source data 2. — This zip archive contains all the fluorescent micrographs used for the quantitative analysis shown in Fig. blank. The individual files are named with the timepoint (for figures containing more than one timepoint), the gene detected by FISH, followed by the color of the label for the gene with ‘G’ for green, ‘R’ for red, ‘W’ for white, and ‘Y’ for yellow. [file elife-56890-fig3-data2.zip › Proliferating macrophages source Files JPEG/Slide11_E18.5_Cd68G_Mki67R.jpeg]

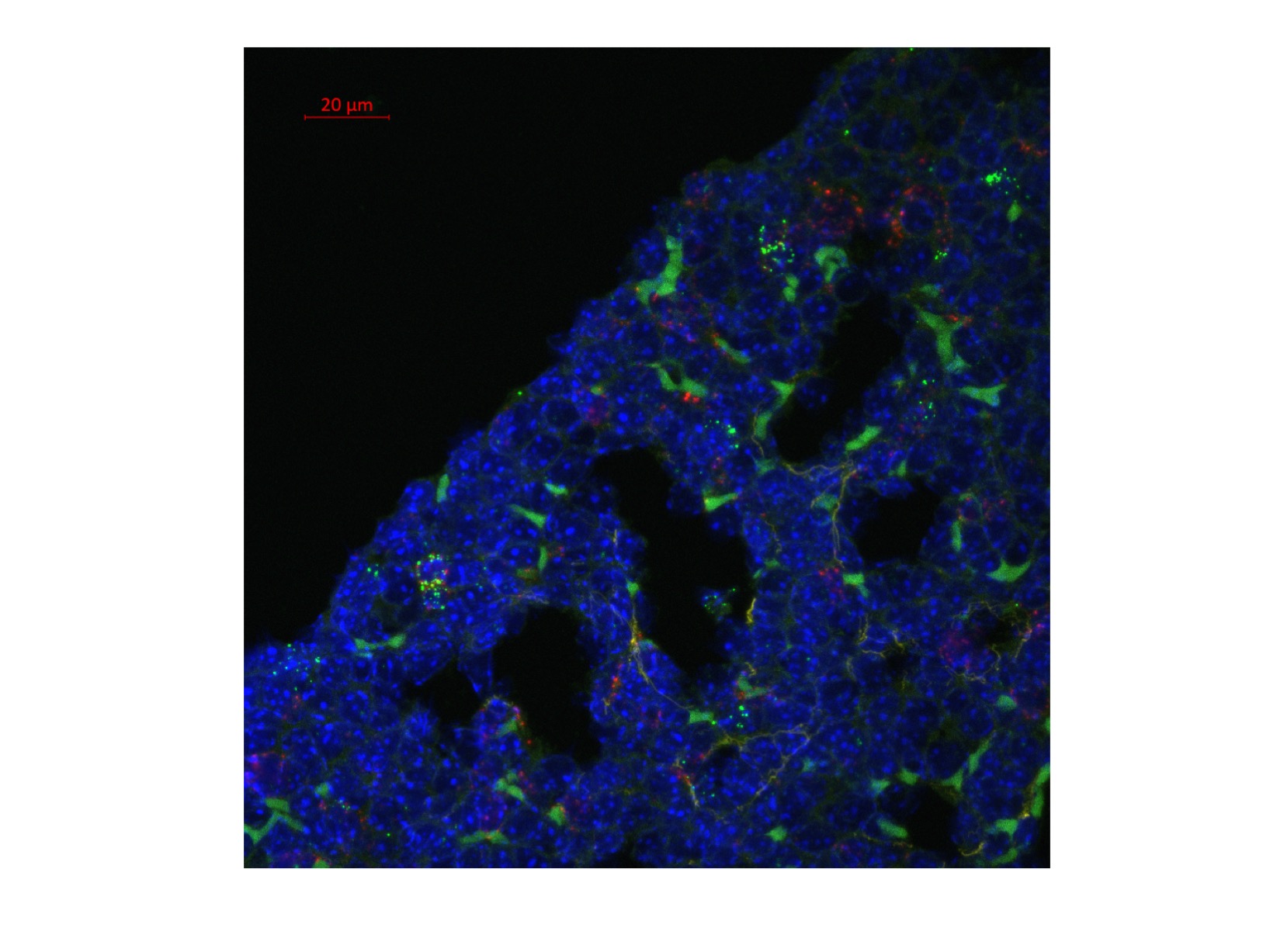

Supplement: Figure 3—source data 2. — This zip archive contains all the fluorescent micrographs used for the quantitative analysis shown in Fig. blank. The individual files are named with the timepoint (for figures containing more than one timepoint), the gene detected by FISH, followed by the color of the label for the gene with ‘G’ for green, ‘R’ for red, ‘W’ for white, and ‘Y’ for yellow. [file elife-56890-fig3-data2.zip › Proliferating macrophages source Files JPEG/Slide12_E18.5_Cd68G_Mki67R.jpeg]

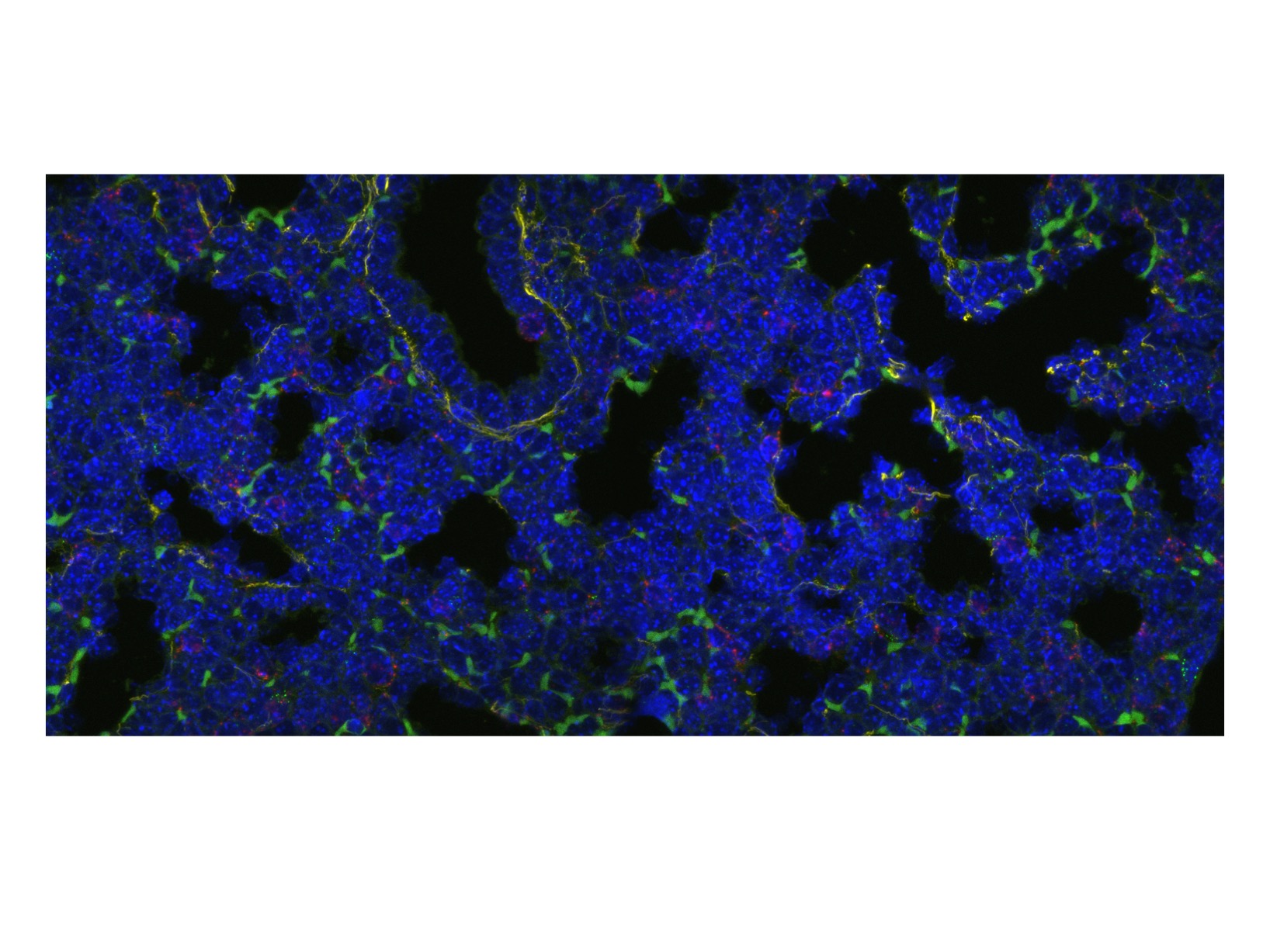

Supplement: Figure 3—source data 2. — This zip archive contains all the fluorescent micrographs used for the quantitative analysis shown in Fig. blank. The individual files are named with the timepoint (for figures containing more than one timepoint), the gene detected by FISH, followed by the color of the label for the gene with ‘G’ for green, ‘R’ for red, ‘W’ for white, and ‘Y’ for yellow. [file elife-56890-fig3-data2.zip › Proliferating macrophages source Files JPEG/Slide13_E18.5_Cd68G_Mki67R.jpeg]

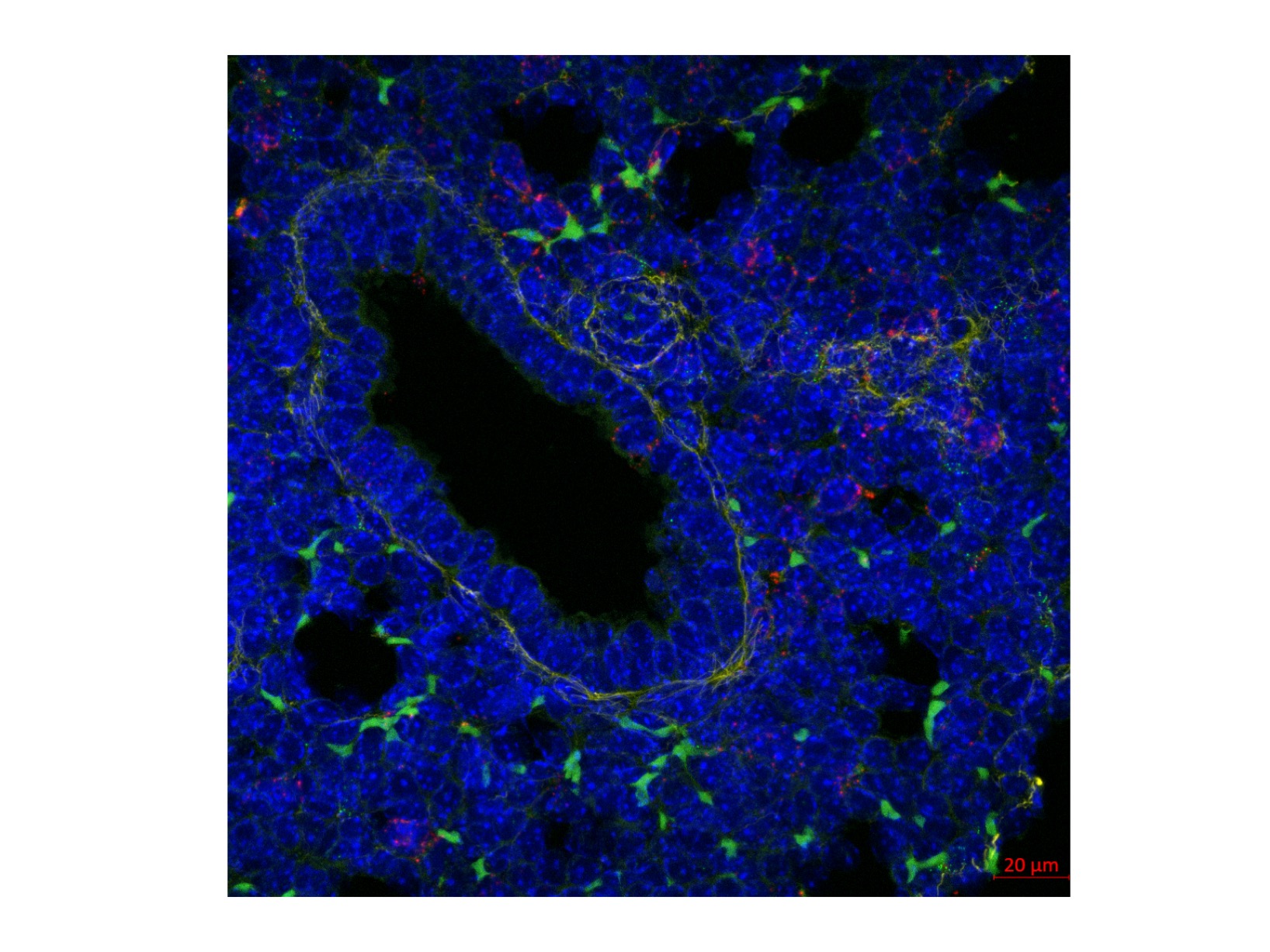

Supplement: Figure 3—source data 2. — This zip archive contains all the fluorescent micrographs used for the quantitative analysis shown in Fig. blank. The individual files are named with the timepoint (for figures containing more than one timepoint), the gene detected by FISH, followed by the color of the label for the gene with ‘G’ for green, ‘R’ for red, ‘W’ for white, and ‘Y’ for yellow. [file elife-56890-fig3-data2.zip › Proliferating macrophages source Files JPEG/Slide14_E18.5_Cd68G_Mki67R.jpeg]

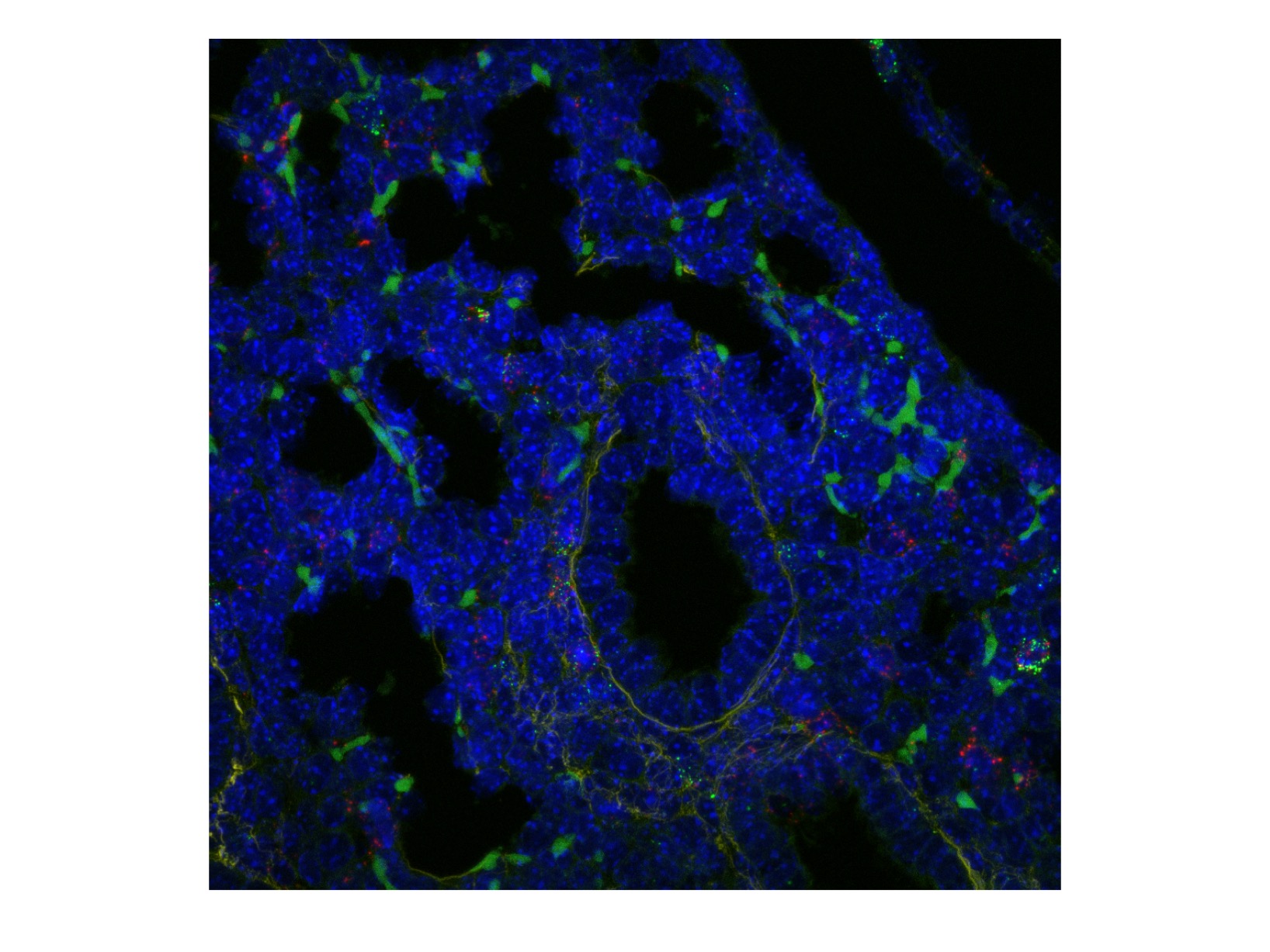

Supplement: Figure 3—source data 2. — This zip archive contains all the fluorescent micrographs used for the quantitative analysis shown in Fig. blank. The individual files are named with the timepoint (for figures containing more than one timepoint), the gene detected by FISH, followed by the color of the label for the gene with ‘G’ for green, ‘R’ for red, ‘W’ for white, and ‘Y’ for yellow. [file elife-56890-fig3-data2.zip › Proliferating macrophages source Files JPEG/Slide15_E18.5_Cd68G_Mki67R.jpeg]

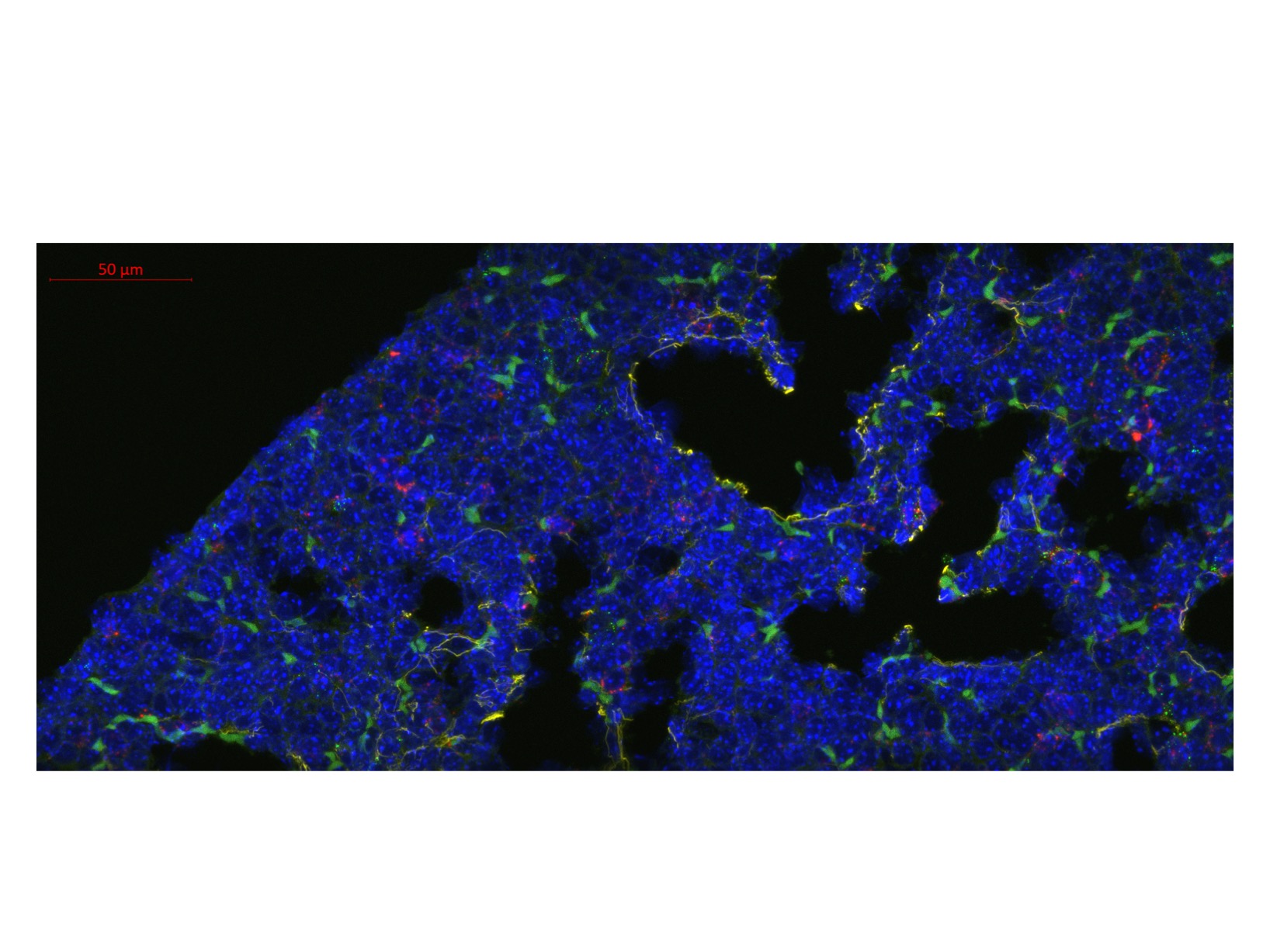

Supplement: Figure 3—source data 2. — This zip archive contains all the fluorescent micrographs used for the quantitative analysis shown in Fig. blank. The individual files are named with the timepoint (for figures containing more than one timepoint), the gene detected by FISH, followed by the color of the label for the gene with ‘G’ for green, ‘R’ for red, ‘W’ for white, and ‘Y’ for yellow. [file elife-56890-fig3-data2.zip › Proliferating macrophages source Files JPEG/Slide16_E18.5_Cd68G_Mki67R.jpeg]

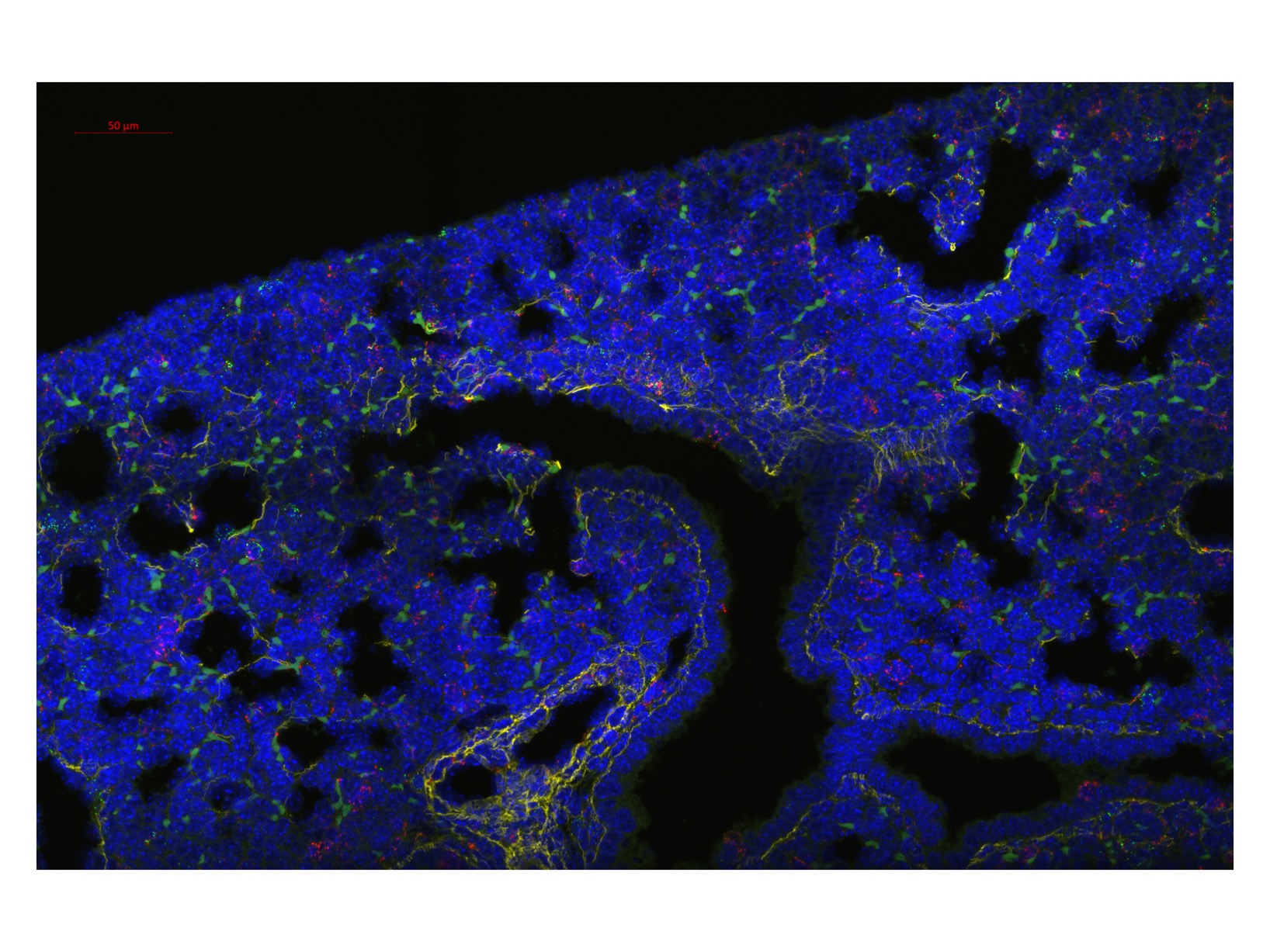

Supplement: Figure 3—source data 2. — This zip archive contains all the fluorescent micrographs used for the quantitative analysis shown in Fig. blank. The individual files are named with the timepoint (for figures containing more than one timepoint), the gene detected by FISH, followed by the color of the label for the gene with ‘G’ for green, ‘R’ for red, ‘W’ for white, and ‘Y’ for yellow. [file elife-56890-fig3-data2.zip › Proliferating macrophages source Files JPEG/Slide17_E18.5_Cd68G_Mki67R.jpeg]

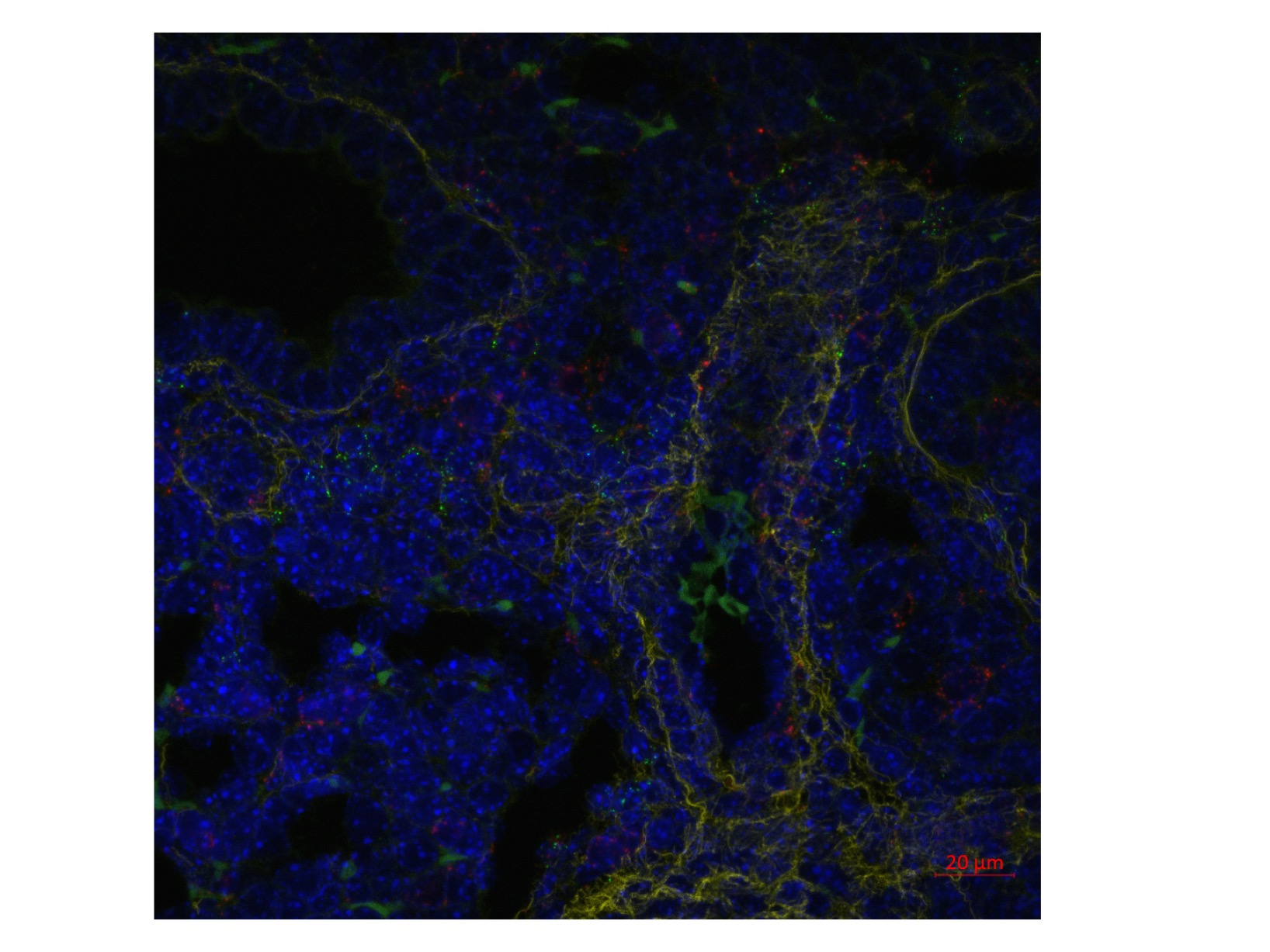

Supplement: Figure 3—source data 2. — This zip archive contains all the fluorescent micrographs used for the quantitative analysis shown in Fig. blank. The individual files are named with the timepoint (for figures containing more than one timepoint), the gene detected by FISH, followed by the color of the label for the gene with ‘G’ for green, ‘R’ for red, ‘W’ for white, and ‘Y’ for yellow. [file elife-56890-fig3-data2.zip › Proliferating macrophages source Files JPEG/Slide1_E18.5_Cd68G_Mki67R.jpeg]

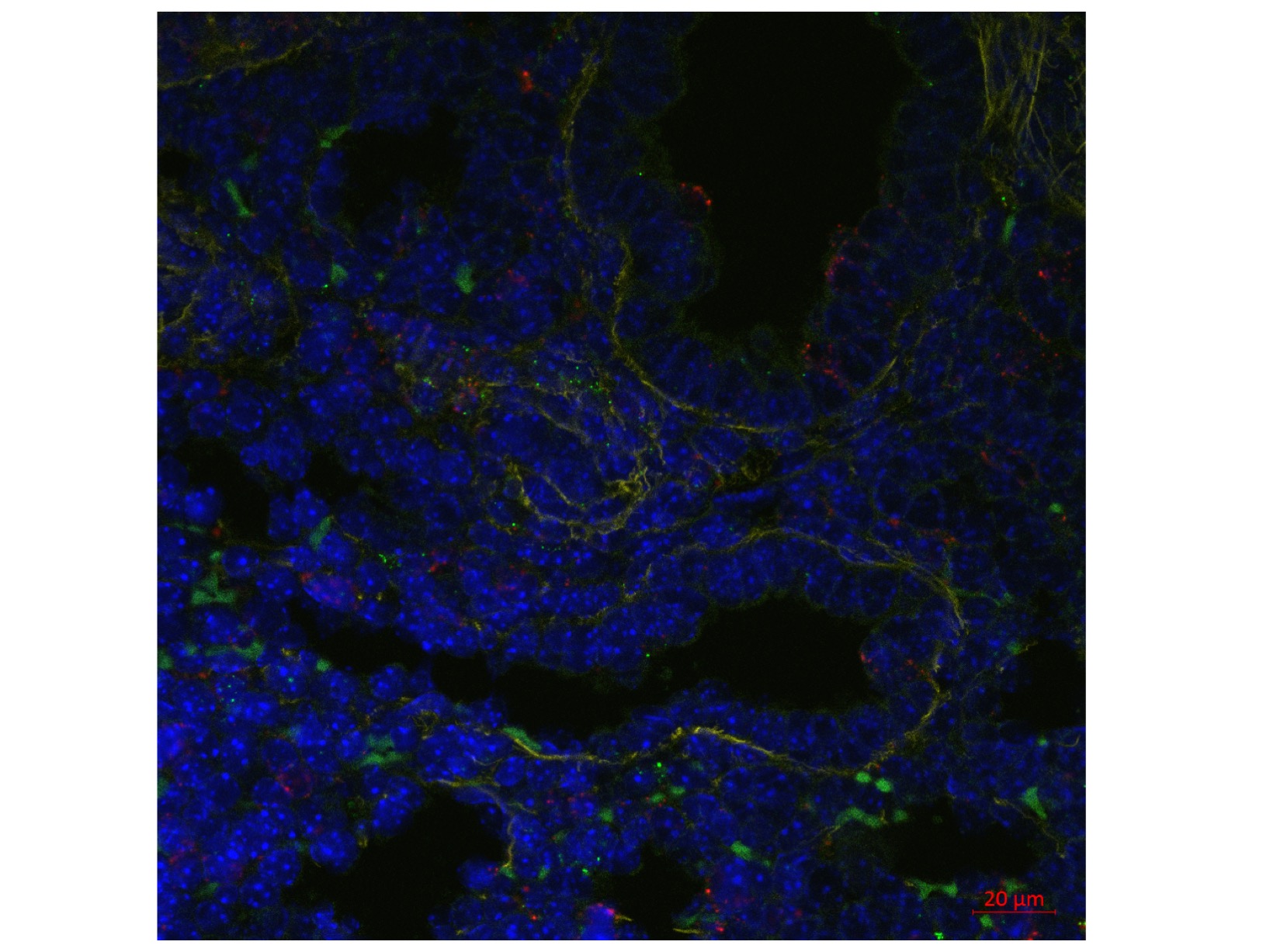

Supplement: Figure 3—source data 2. — This zip archive contains all the fluorescent micrographs used for the quantitative analysis shown in Fig. blank. The individual files are named with the timepoint (for figures containing more than one timepoint), the gene detected by FISH, followed by the color of the label for the gene with ‘G’ for green, ‘R’ for red, ‘W’ for white, and ‘Y’ for yellow. [file elife-56890-fig3-data2.zip › Proliferating macrophages source Files JPEG/Slide2_E18.5_Cd68G_Mki67R.jpeg]

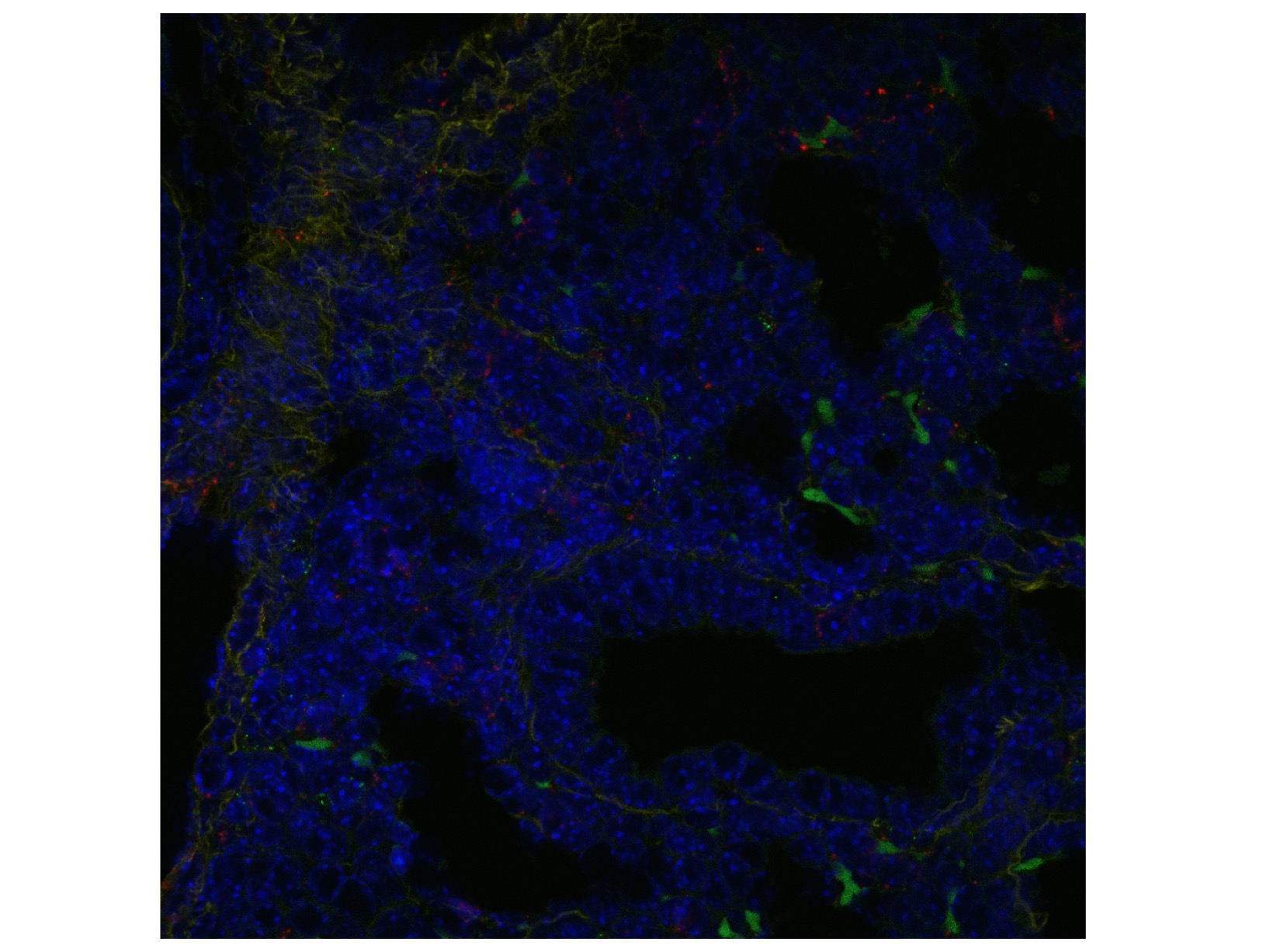

Supplement: Figure 3—source data 2. — This zip archive contains all the fluorescent micrographs used for the quantitative analysis shown in Fig. blank. The individual files are named with the timepoint (for figures containing more than one timepoint), the gene detected by FISH, followed by the color of the label for the gene with ‘G’ for green, ‘R’ for red, ‘W’ for white, and ‘Y’ for yellow. [file elife-56890-fig3-data2.zip › Proliferating macrophages source Files JPEG/Slide3_E18.5_Cd68G_Mki67R.jpeg]

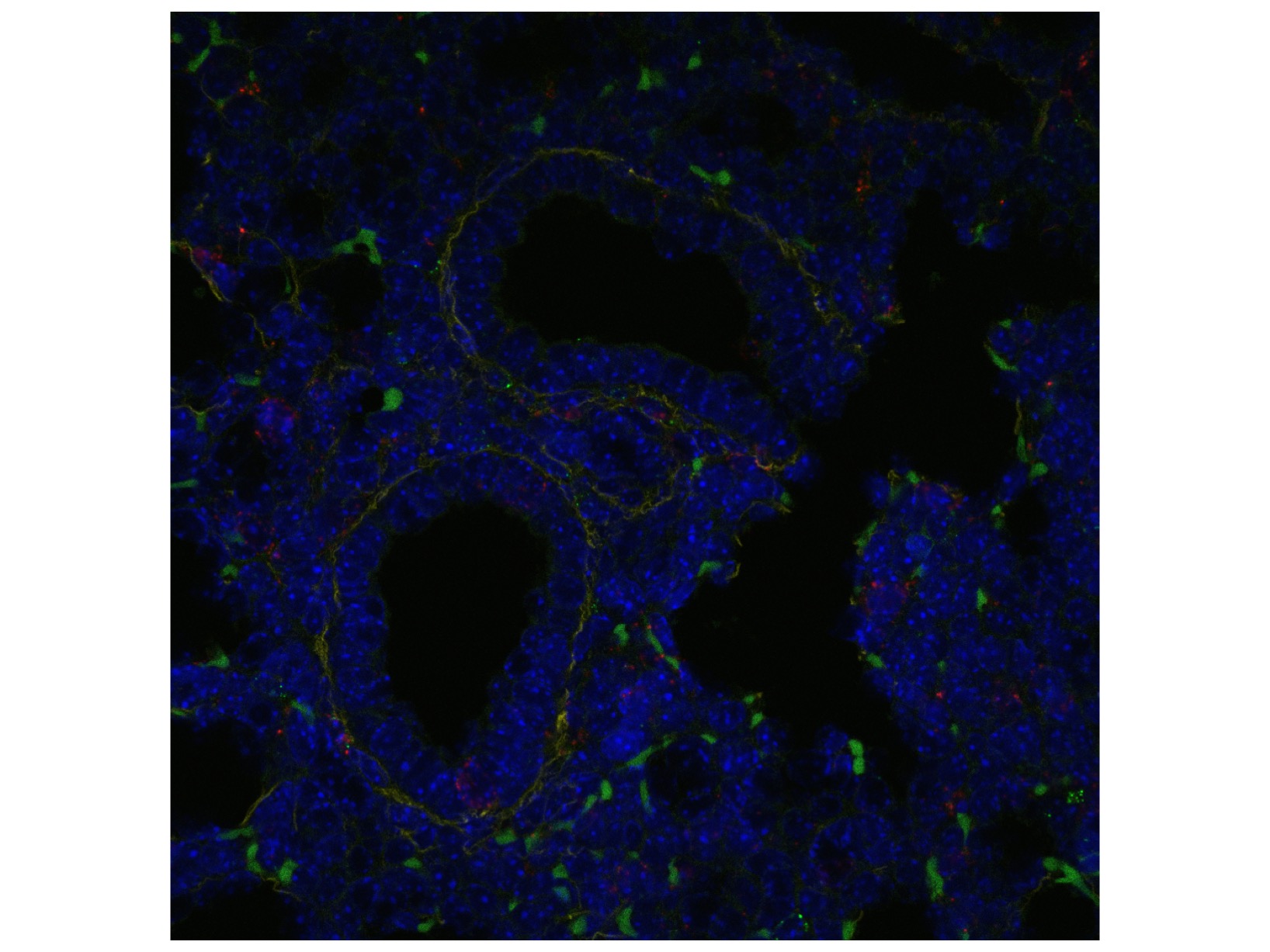

Supplement: Figure 3—source data 2. — This zip archive contains all the fluorescent micrographs used for the quantitative analysis shown in Fig. blank. The individual files are named with the timepoint (for figures containing more than one timepoint), the gene detected by FISH, followed by the color of the label for the gene with ‘G’ for green, ‘R’ for red, ‘W’ for white, and ‘Y’ for yellow. [file elife-56890-fig3-data2.zip › Proliferating macrophages source Files JPEG/Slide4_E18.5_Cd68G_Mki67R.jpeg]

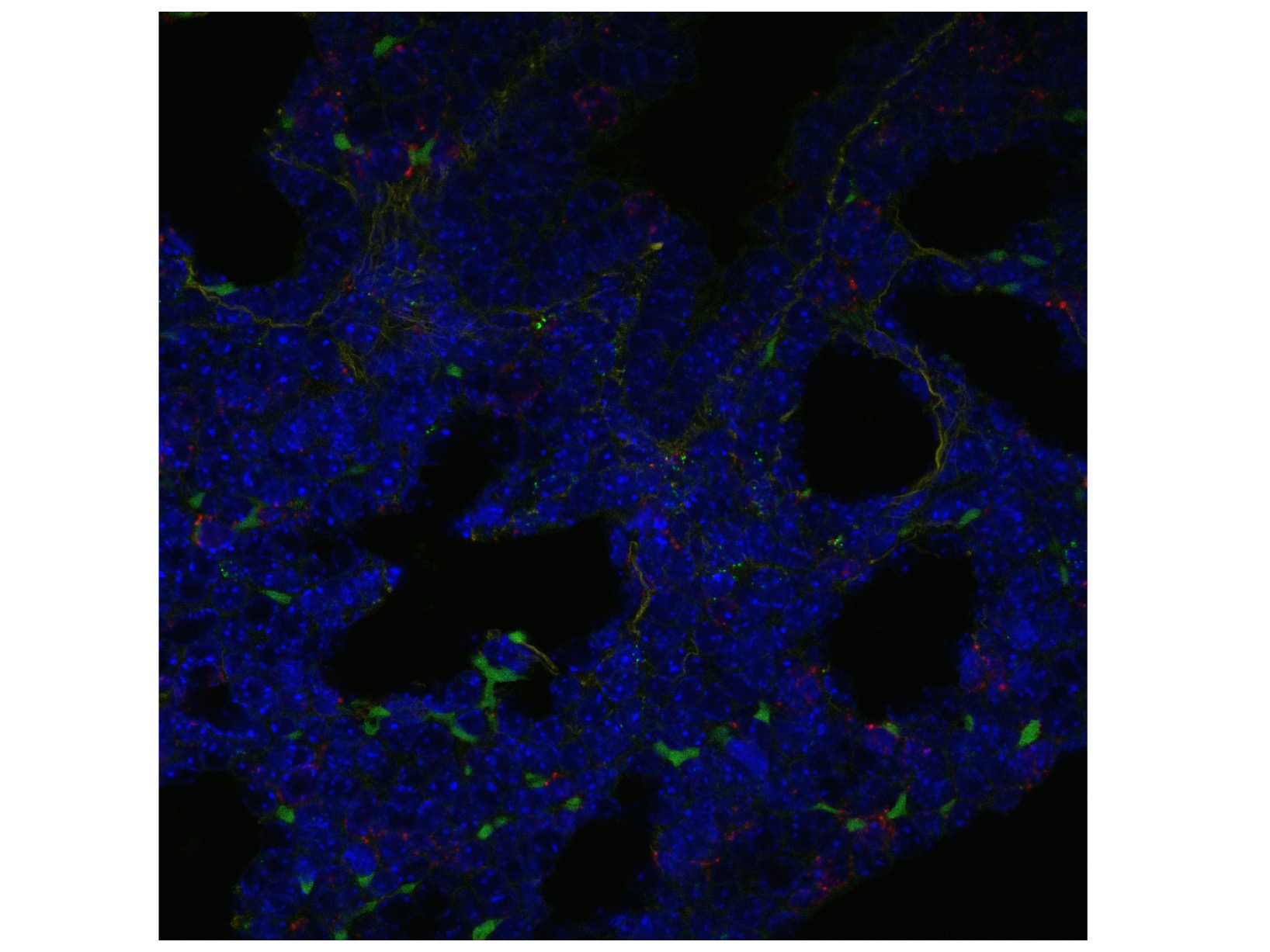

Supplement: Figure 3—source data 2. — This zip archive contains all the fluorescent micrographs used for the quantitative analysis shown in Fig. blank. The individual files are named with the timepoint (for figures containing more than one timepoint), the gene detected by FISH, followed by the color of the label for the gene with ‘G’ for green, ‘R’ for red, ‘W’ for white, and ‘Y’ for yellow. [file elife-56890-fig3-data2.zip › Proliferating macrophages source Files JPEG/Slide5_E18.5_Cd68G_Mki67R.jpeg]

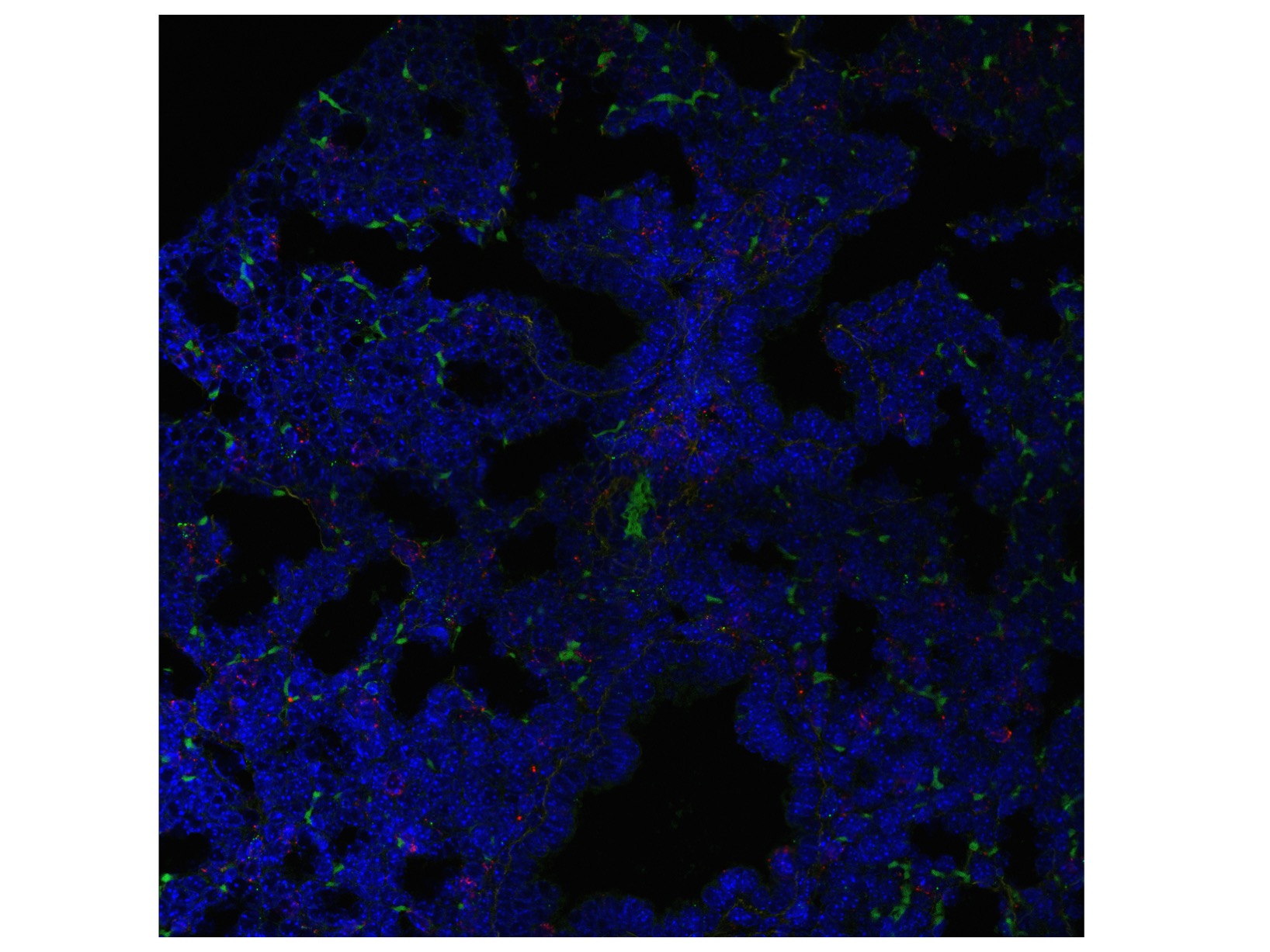

Supplement: Figure 3—source data 2. — This zip archive contains all the fluorescent micrographs used for the quantitative analysis shown in Fig. blank. The individual files are named with the timepoint (for figures containing more than one timepoint), the gene detected by FISH, followed by the color of the label for the gene with ‘G’ for green, ‘R’ for red, ‘W’ for white, and ‘Y’ for yellow. [file elife-56890-fig3-data2.zip › Proliferating macrophages source Files JPEG/Slide6_E18.5_Cd68G_Mki67R.jpeg]

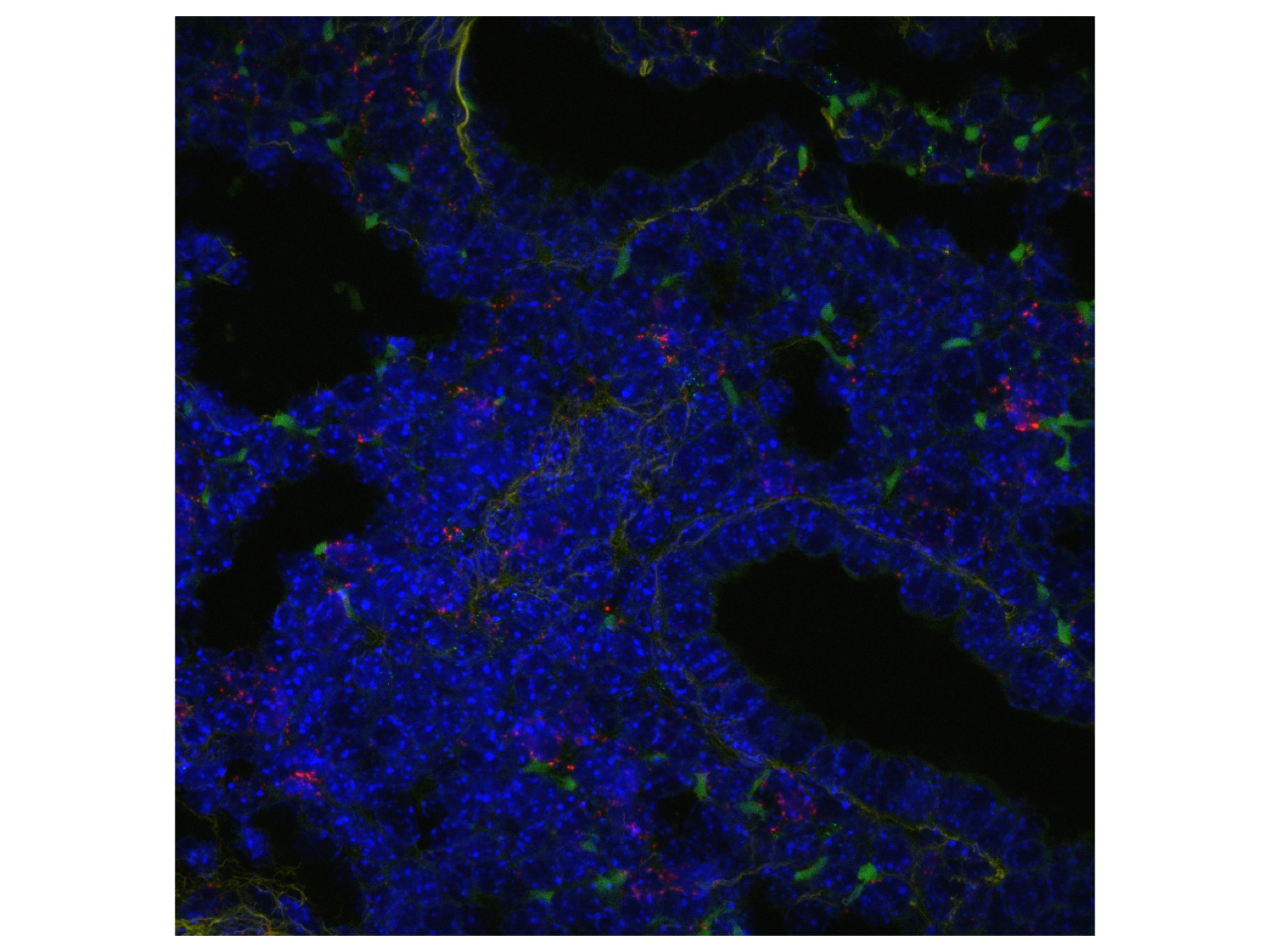

Supplement: Figure 3—source data 2. — This zip archive contains all the fluorescent micrographs used for the quantitative analysis shown in Fig. blank. The individual files are named with the timepoint (for figures containing more than one timepoint), the gene detected by FISH, followed by the color of the label for the gene with ‘G’ for green, ‘R’ for red, ‘W’ for white, and ‘Y’ for yellow. [file elife-56890-fig3-data2.zip › Proliferating macrophages source Files JPEG/Slide7_E18.5_Cd68G_Mki67R.jpeg]

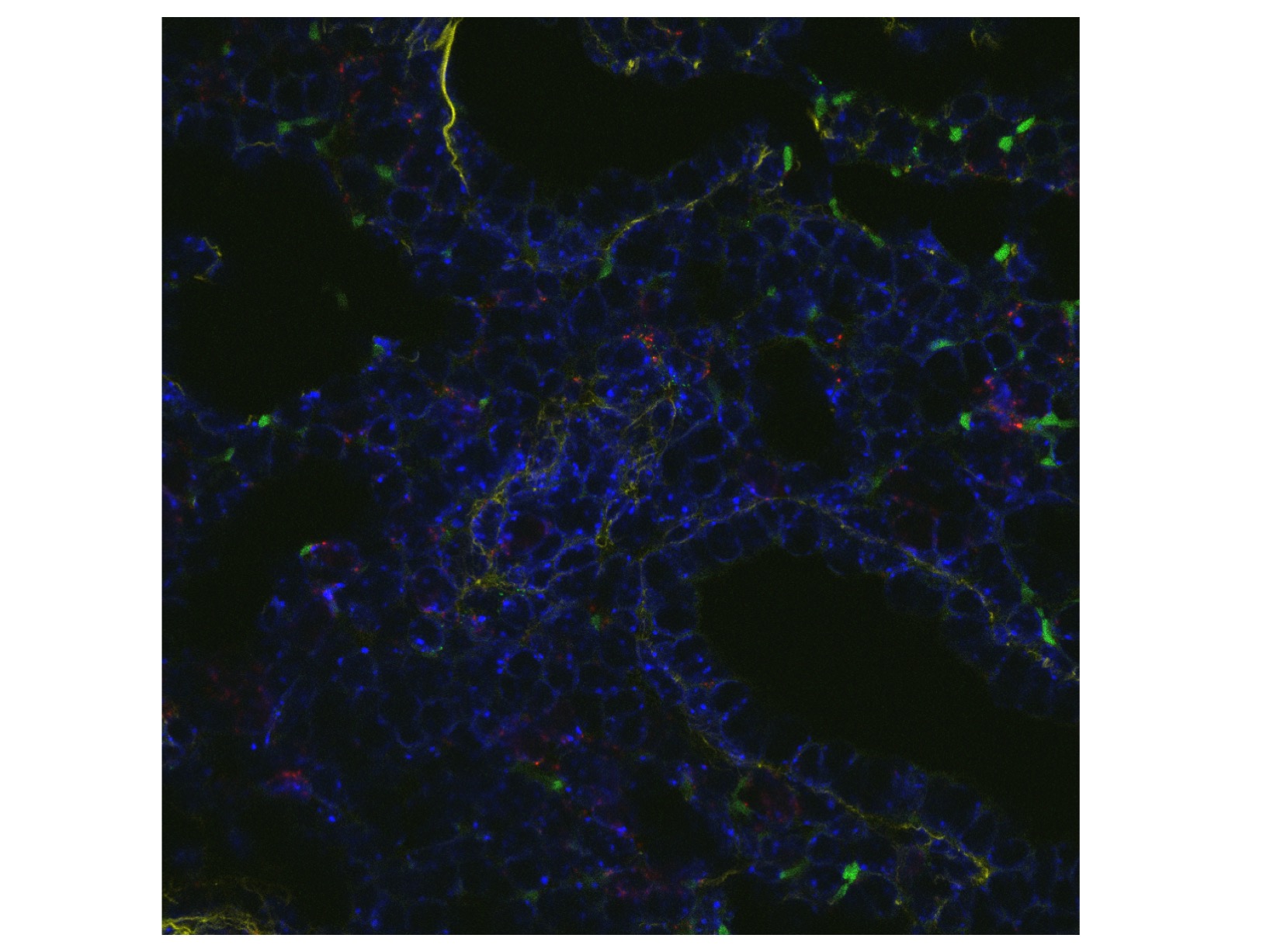

Supplement: Figure 3—source data 2. — This zip archive contains all the fluorescent micrographs used for the quantitative analysis shown in Fig. blank. The individual files are named with the timepoint (for figures containing more than one timepoint), the gene detected by FISH, followed by the color of the label for the gene with ‘G’ for green, ‘R’ for red, ‘W’ for white, and ‘Y’ for yellow. [file elife-56890-fig3-data2.zip › Proliferating macrophages source Files JPEG/Slide8_E18.5_Cd68G_Mki67R.jpeg]

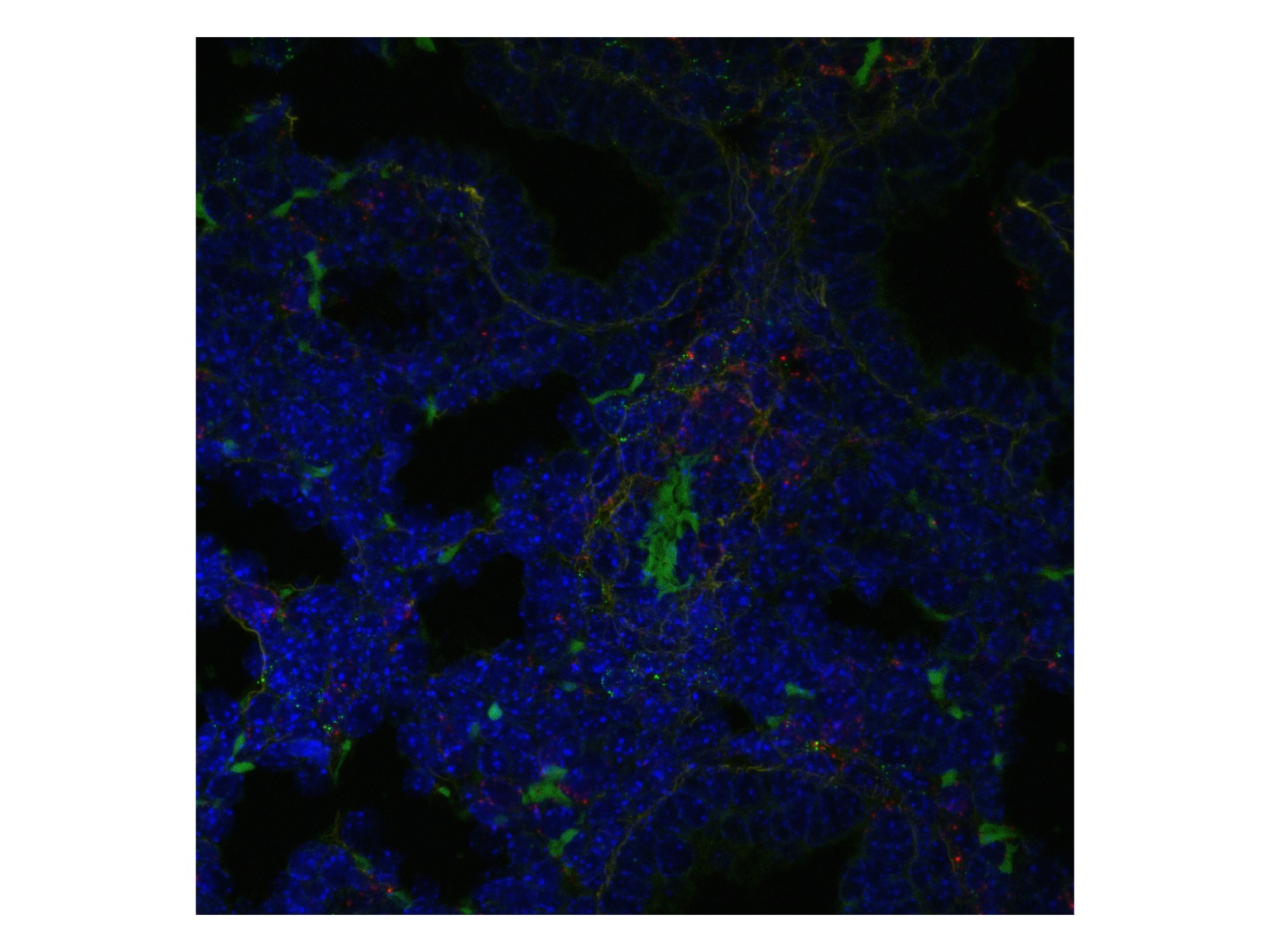

Supplement: Figure 3—source data 2. — This zip archive contains all the fluorescent micrographs used for the quantitative analysis shown in Fig. blank. The individual files are named with the timepoint (for figures containing more than one timepoint), the gene detected by FISH, followed by the color of the label for the gene with ‘G’ for green, ‘R’ for red, ‘W’ for white, and ‘Y’ for yellow. [file elife-56890-fig3-data2.zip › Proliferating macrophages source Files JPEG/Slide9_E18.5_Cd68G_Mki67R.jpeg]

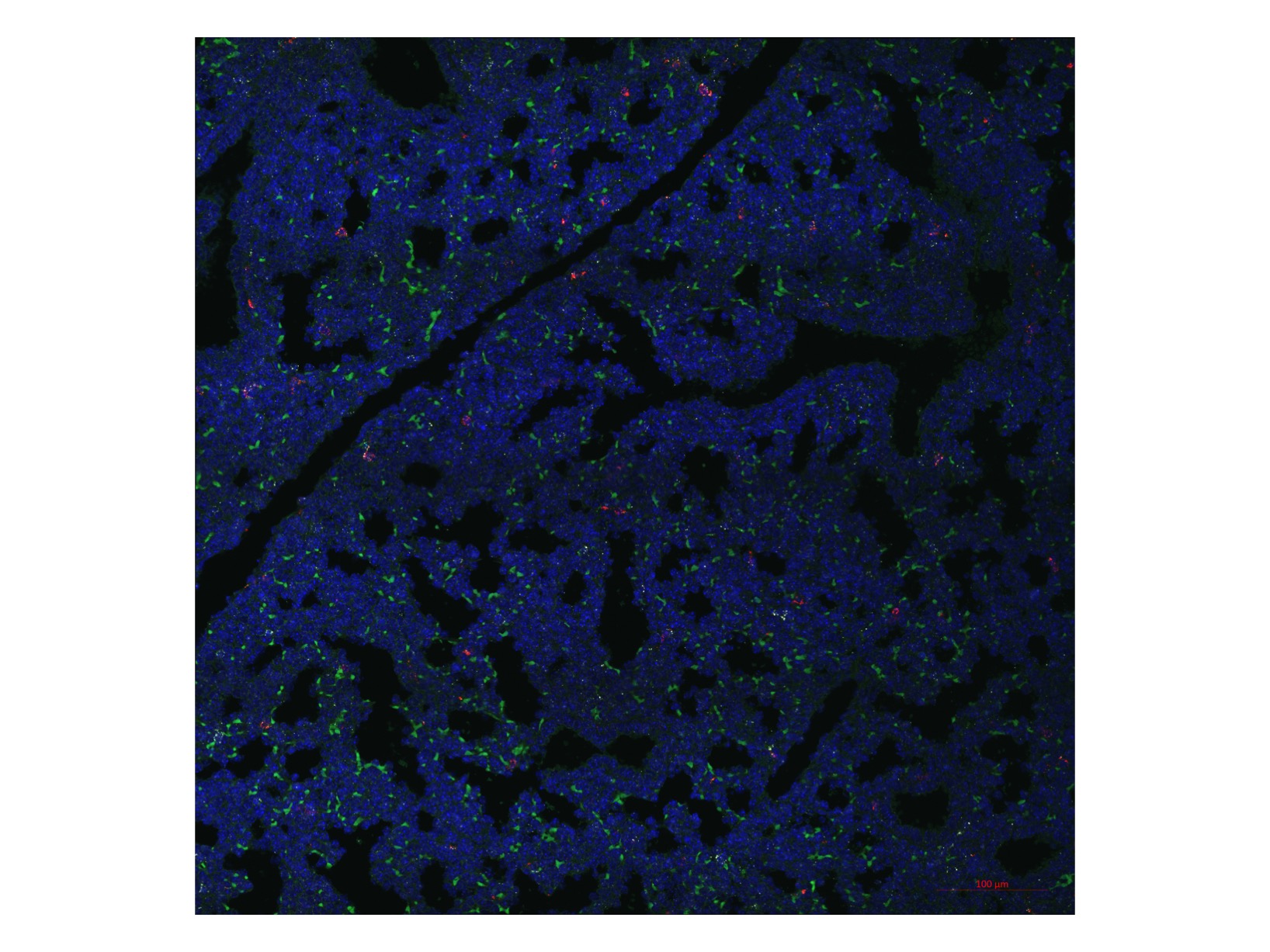

Supplement: Figure 3—source data 3. — This zip archive contains all the fluorescent micrographs used for the quantitative analysis shown in Fig. blank. The individual files are named with the timepoint (for figures containing more than one timepoint), the gene detected by FISH, followed by the color of the label for the gene with ‘G’ for green, ‘R’ for red, ‘W’ for white, and ‘Y’ for yellow. [file elife-56890-fig3-data3.zip › Source Files for Gal and C1qa JPEG/Slide1_E18.5_Cd68W_GalR_C1qaG.jpeg]

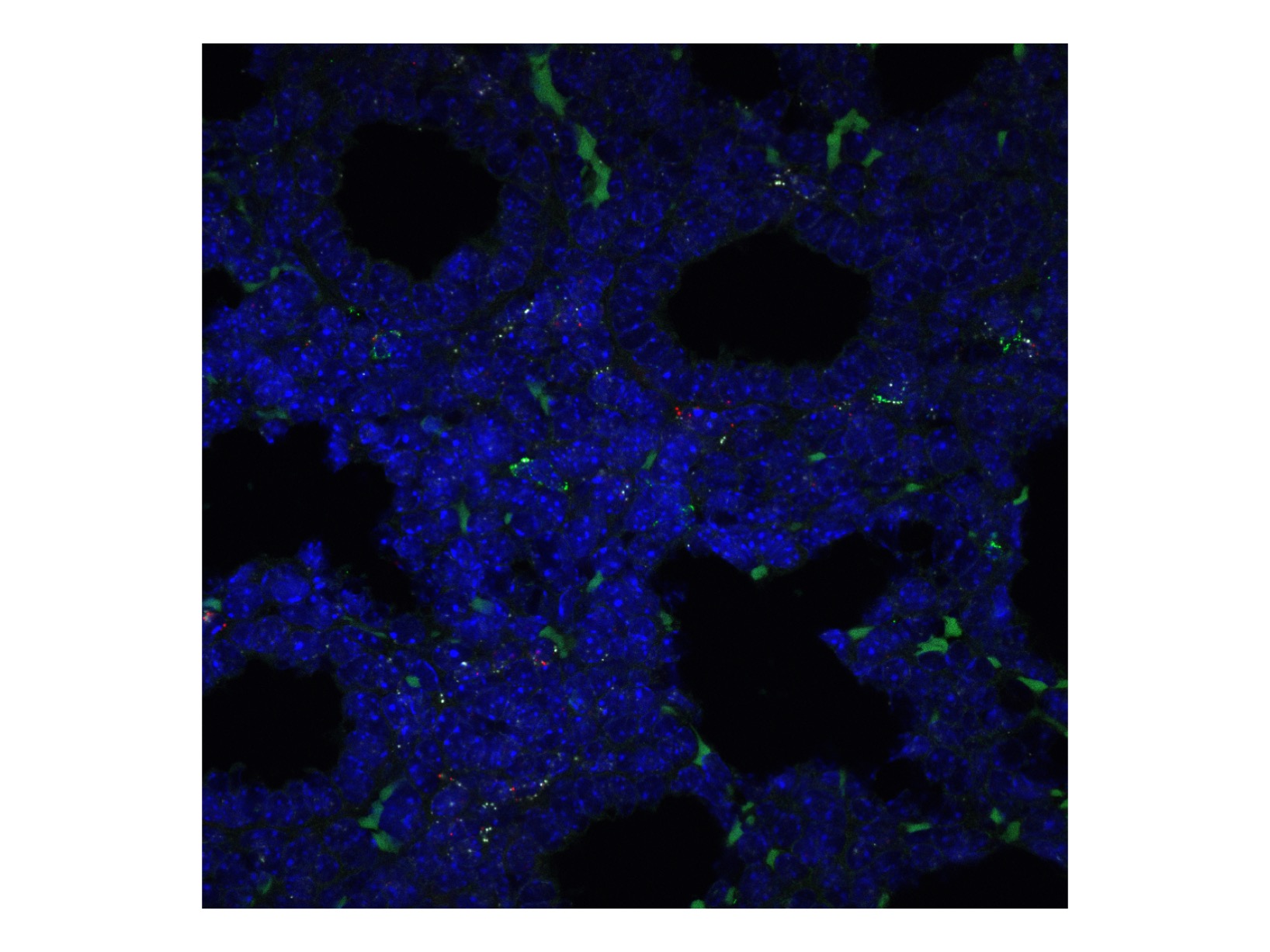

Supplement: Figure 3—source data 3. — This zip archive contains all the fluorescent micrographs used for the quantitative analysis shown in Fig. blank. The individual files are named with the timepoint (for figures containing more than one timepoint), the gene detected by FISH, followed by the color of the label for the gene with ‘G’ for green, ‘R’ for red, ‘W’ for white, and ‘Y’ for yellow. [file elife-56890-fig3-data3.zip › Source Files for Gal and C1qa JPEG/Slide2_E18.5_Cd68W_GalR_C1qaG.jpeg]

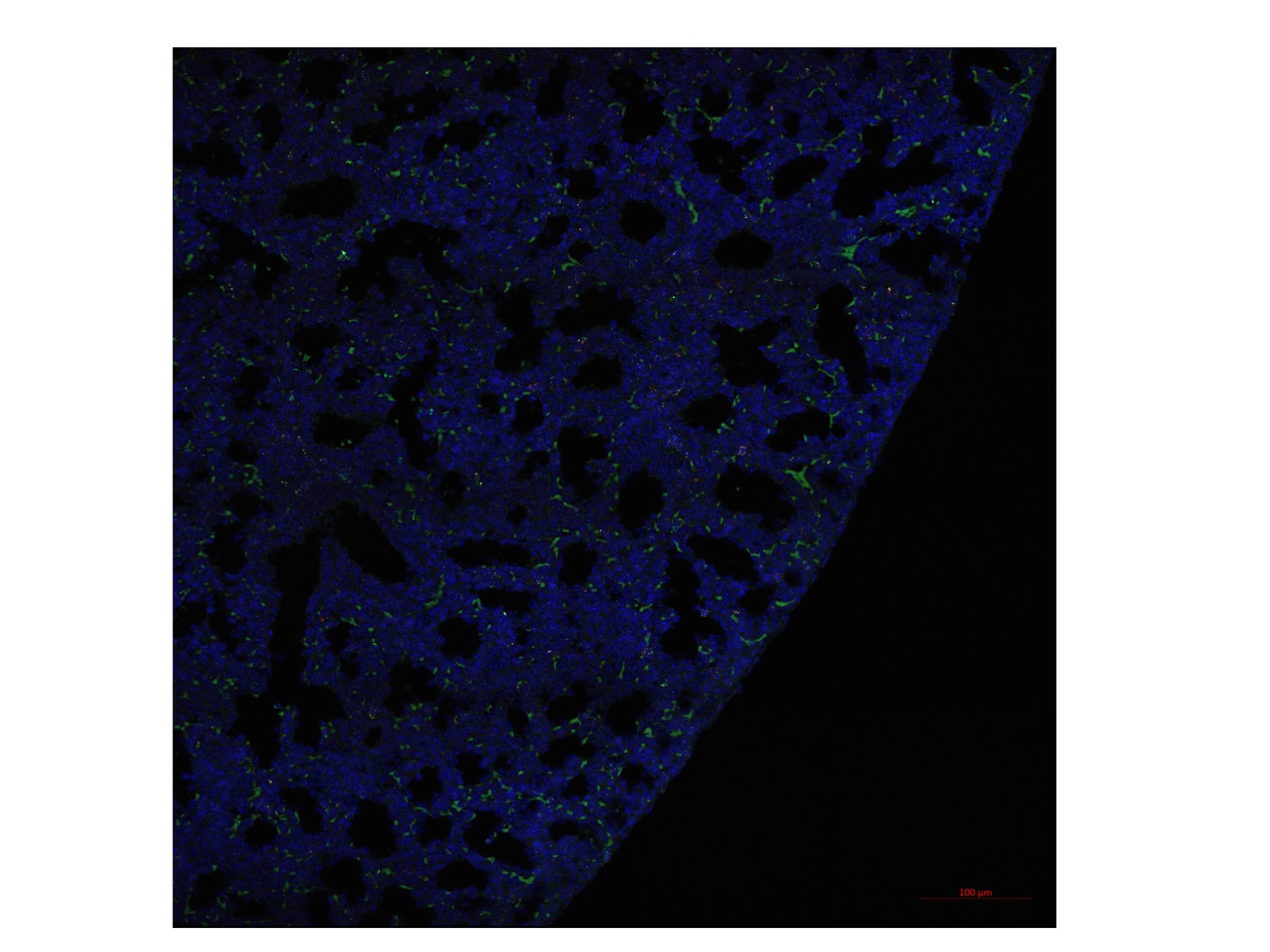

Supplement: Figure 3—source data 3. — This zip archive contains all the fluorescent micrographs used for the quantitative analysis shown in Fig. blank. The individual files are named with the timepoint (for figures containing more than one timepoint), the gene detected by FISH, followed by the color of the label for the gene with ‘G’ for green, ‘R’ for red, ‘W’ for white, and ‘Y’ for yellow. [file elife-56890-fig3-data3.zip › Source Files for Gal and C1qa JPEG/Slide3_E18.5_Cd68W_GalR_C1qaG.jpeg]

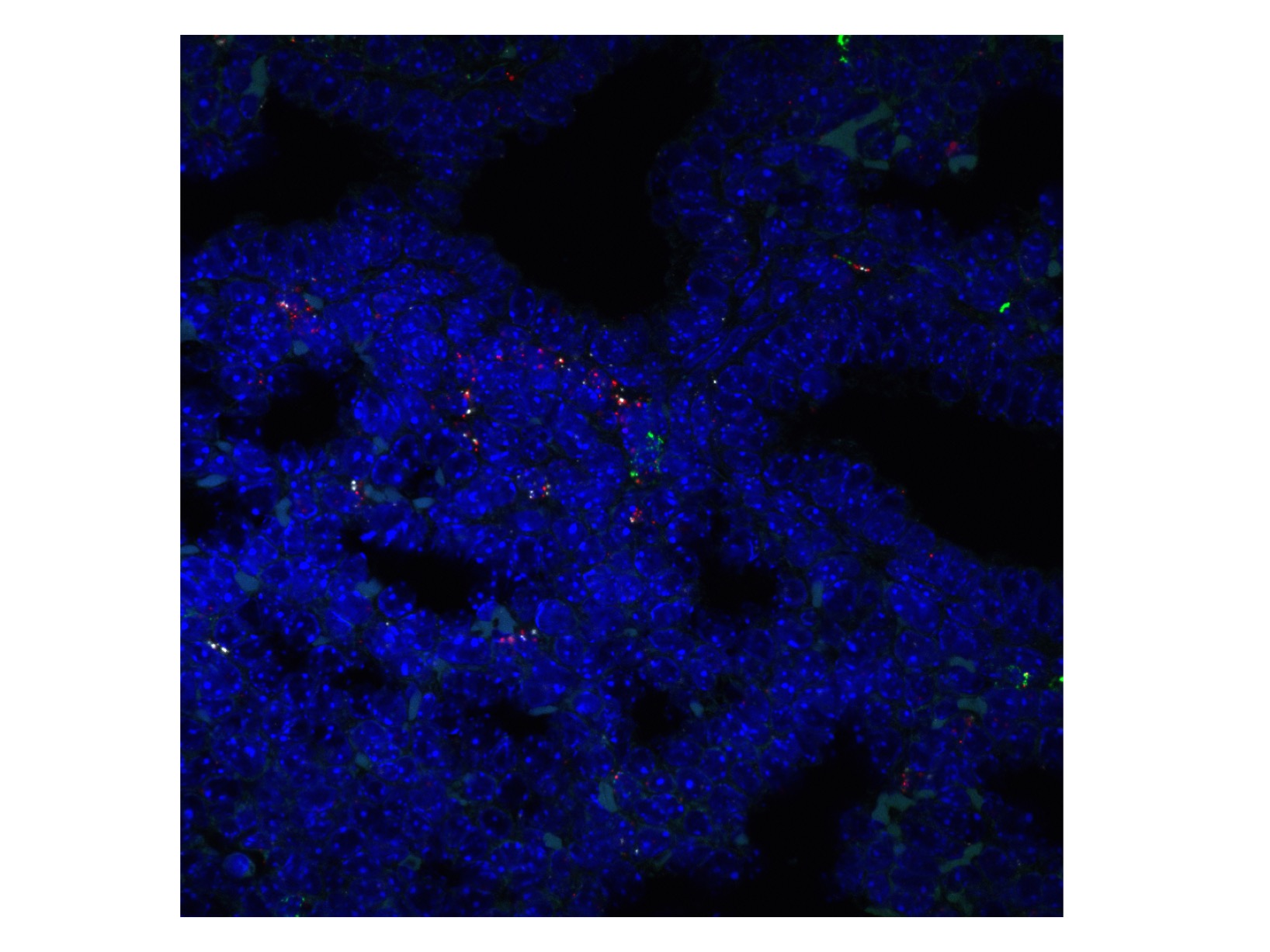

Supplement: Figure 3—source data 3. — This zip archive contains all the fluorescent micrographs used for the quantitative analysis shown in Fig. blank. The individual files are named with the timepoint (for figures containing more than one timepoint), the gene detected by FISH, followed by the color of the label for the gene with ‘G’ for green, ‘R’ for red, ‘W’ for white, and ‘Y’ for yellow. [file elife-56890-fig3-data3.zip › Source Files for Gal and C1qa JPEG/Slide4_E18.5_Cd68R_GalW_C1qaG.jpeg]

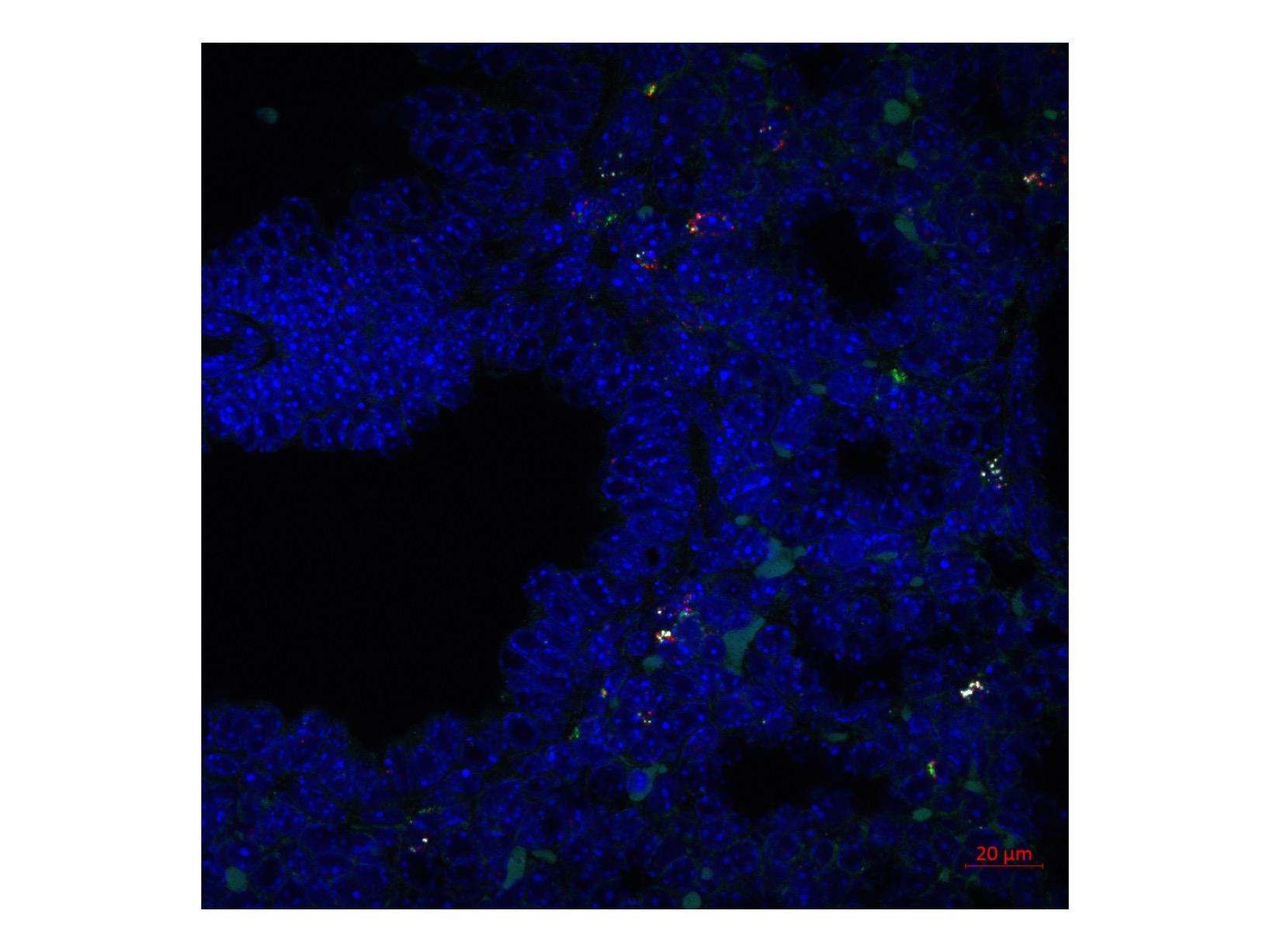

Supplement: Figure 3—source data 3. — This zip archive contains all the fluorescent micrographs used for the quantitative analysis shown in Fig. blank. The individual files are named with the timepoint (for figures containing more than one timepoint), the gene detected by FISH, followed by the color of the label for the gene with ‘G’ for green, ‘R’ for red, ‘W’ for white, and ‘Y’ for yellow. [file elife-56890-fig3-data3.zip › Source Files for Gal and C1qa JPEG/Slide5_E18.5_Cd68R_GalW_C1qaG.jpeg]

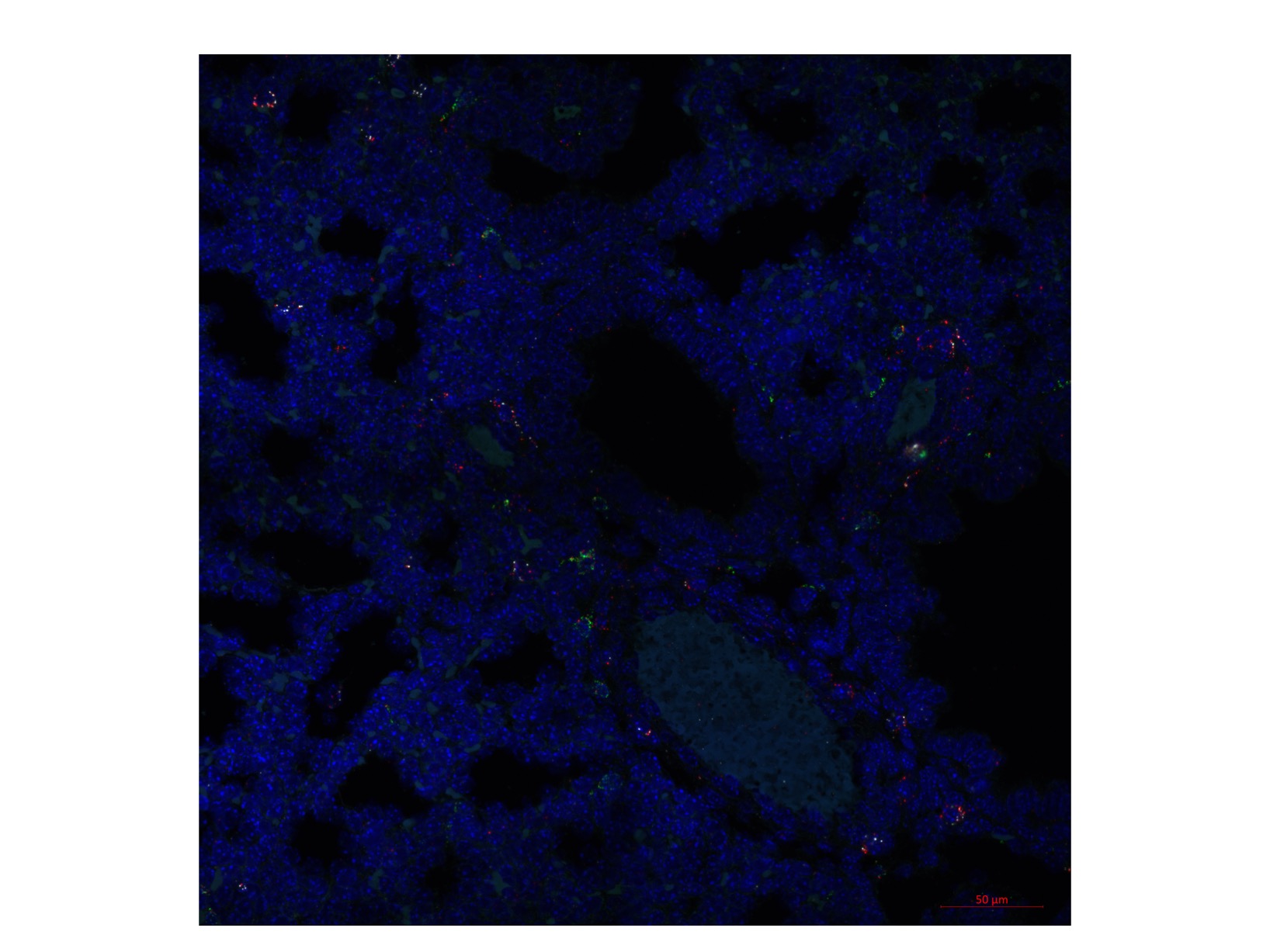

Supplement: Figure 3—source data 3. — This zip archive contains all the fluorescent micrographs used for the quantitative analysis shown in Fig. blank. The individual files are named with the timepoint (for figures containing more than one timepoint), the gene detected by FISH, followed by the color of the label for the gene with ‘G’ for green, ‘R’ for red, ‘W’ for white, and ‘Y’ for yellow. [file elife-56890-fig3-data3.zip › Source Files for Gal and C1qa JPEG/Slide6_E18.5_Cd68R_GalW_C1qaG.jpeg]

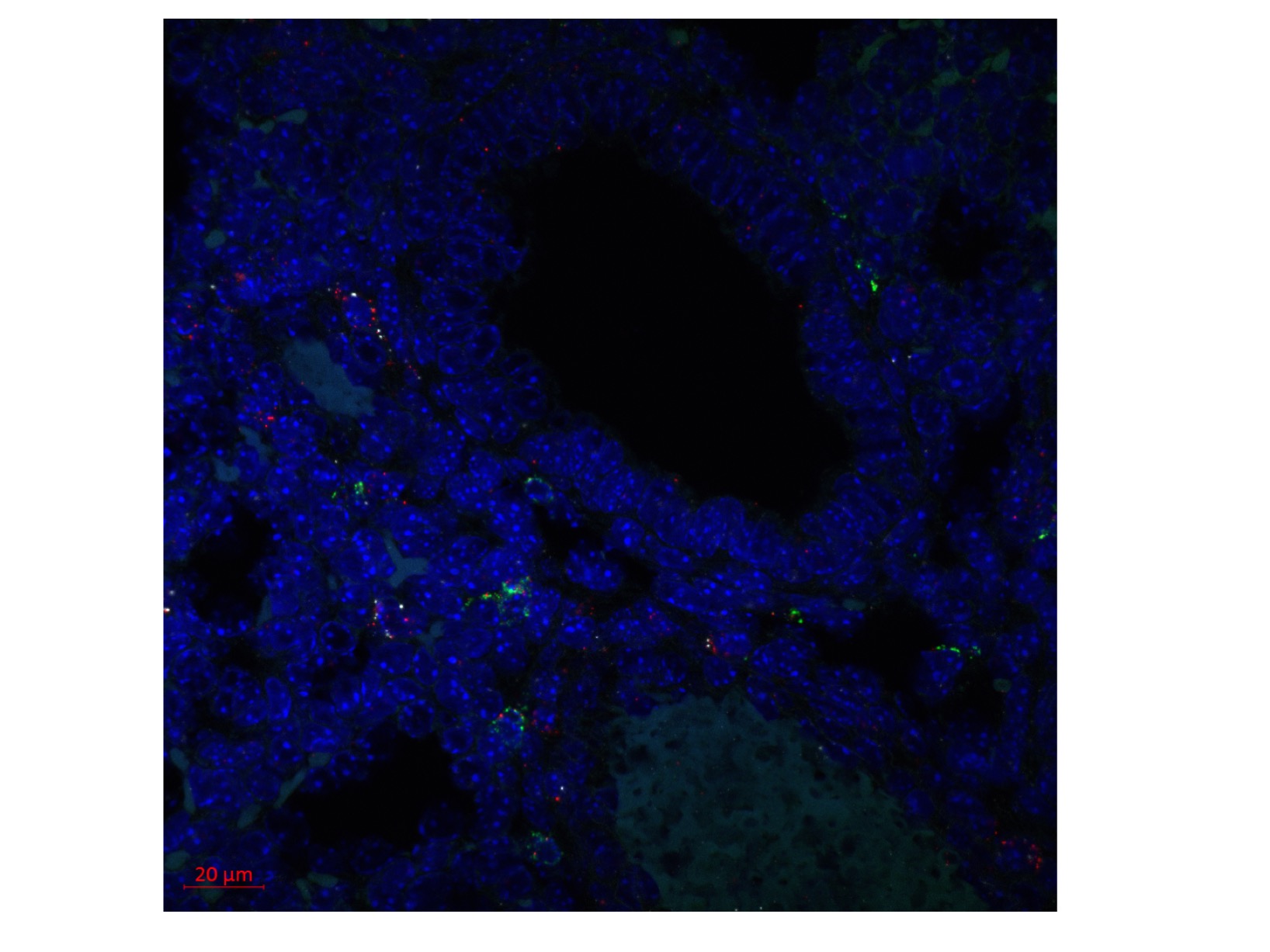

Supplement: Figure 3—source data 3. — This zip archive contains all the fluorescent micrographs used for the quantitative analysis shown in Fig. blank. The individual files are named with the timepoint (for figures containing more than one timepoint), the gene detected by FISH, followed by the color of the label for the gene with ‘G’ for green, ‘R’ for red, ‘W’ for white, and ‘Y’ for yellow. [file elife-56890-fig3-data3.zip › Source Files for Gal and C1qa JPEG/Slide7_E18.5_Cd68R_GalW_C1qaG.jpeg]

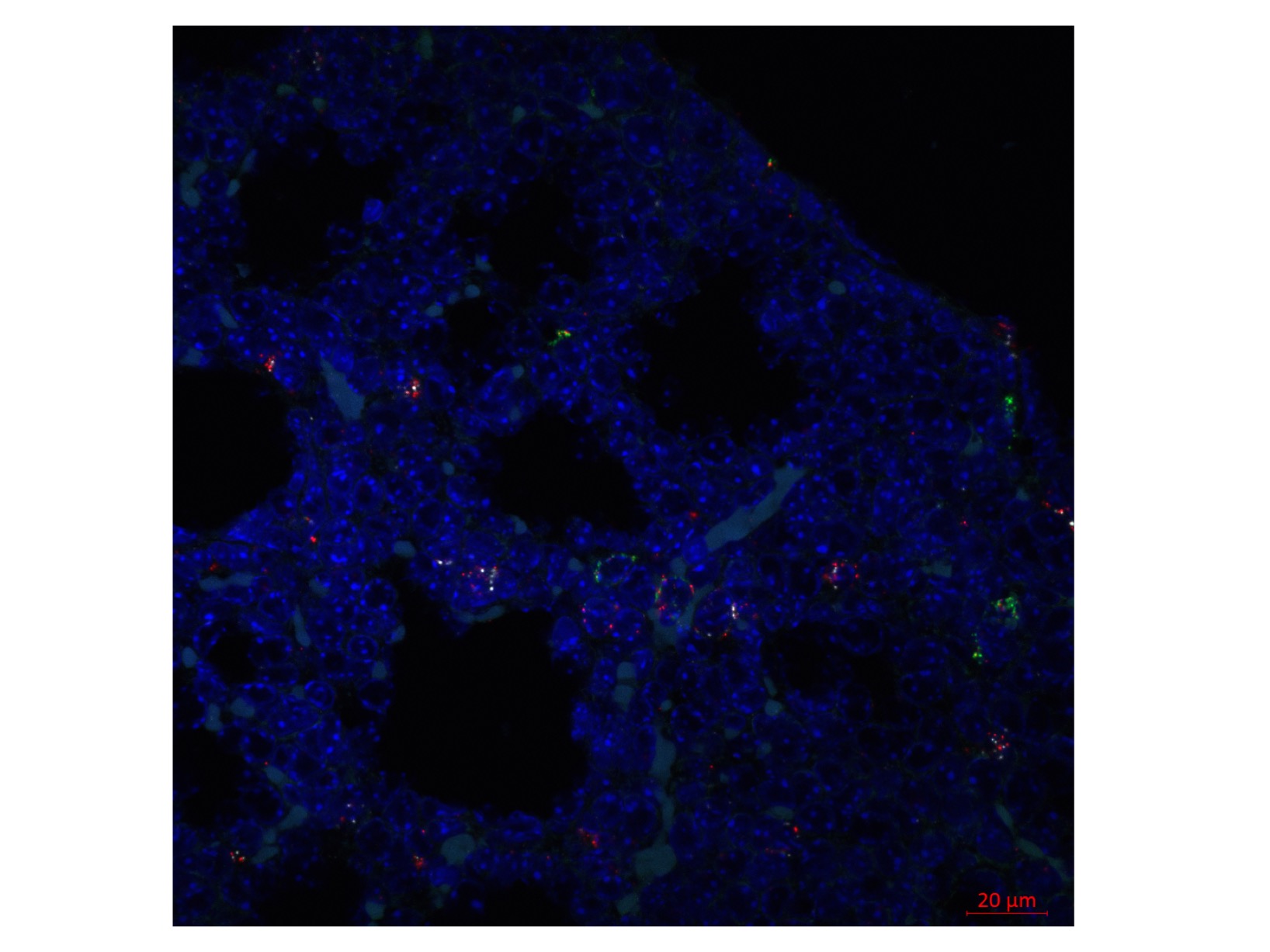

Supplement: Figure 3—source data 3. — This zip archive contains all the fluorescent micrographs used for the quantitative analysis shown in Fig. blank. The individual files are named with the timepoint (for figures containing more than one timepoint), the gene detected by FISH, followed by the color of the label for the gene with ‘G’ for green, ‘R’ for red, ‘W’ for white, and ‘Y’ for yellow. [file elife-56890-fig3-data3.zip › Source Files for Gal and C1qa JPEG/Slide8_E18.5_Cd68R_GalW_C1qaG.jpeg]

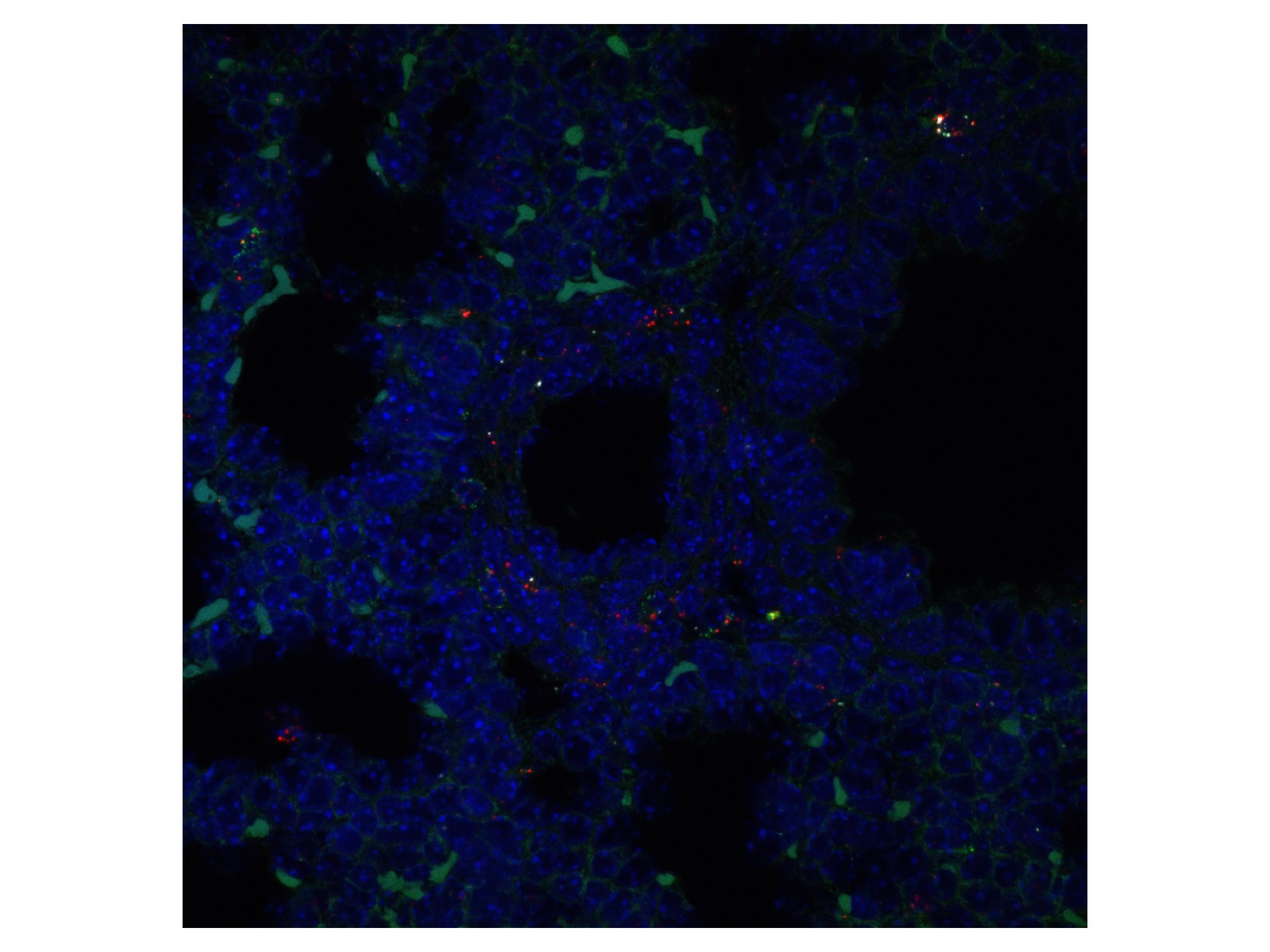

Supplement: Figure 3—source data 3. — This zip archive contains all the fluorescent micrographs used for the quantitative analysis shown in Fig. blank. The individual files are named with the timepoint (for figures containing more than one timepoint), the gene detected by FISH, followed by the color of the label for the gene with ‘G’ for green, ‘R’ for red, ‘W’ for white, and ‘Y’ for yellow. [file elife-56890-fig3-data3.zip › Source Files for Gal and C1qa JPEG/Slide9_E18.5_Cd68R_GalW_C1qaG.jpeg]
