# Supplementary material for: Diverse homeostatic and immunomodulatory roles of immune cells in the developing mouse lung at single cell resolution
Source: eLife. 2020 Jun 2;9:e56890. doi: 10.7554/eLife.56890 (PMC7358008; doi:10.7554/eLife.56890)
Supplement: Transparent reporting form [file elife-56890-transrepform.pdf]

eLIFE
1st Floor
24 Hills Road
Cambridge CB2 1JP, UK
P 01223 855340
W elifesciences.org
T @elife

eLife's transparent reporting form

We encourage authors to provide detailed information *within their submission* to facilitate the interpretation and replication of experiments. Authors can upload supporting documentation to indicate the use of appropriate reporting guidelines for health-related research (see EQUATOR Network), life science research (see the BioSharing Information Resource), or the ARRIVE guidelines for reporting work involving animal research. Where applicable, authors should refer to any relevant reporting standards documents in this form.

If you have any questions, please consult our Journal Policies and/or contact us: editorial@elifesciences.org.

Sample-size estimation

- You should state whether an appropriate sample size was computed when the study was being designed
- You should state the statistical method of sample size computation and any required assumptions
- If no explicit power analysis was used, you should describe how you decided what sample (replicate) size (number) to use

Please outline where this information can be found within the submission (e.g., sections or figure legends), or explain why this information doesn't apply to your submission:

The number of cells and average number of read per cell is located in the Main Text of the manuscript. For the single cell studies, the authors decided on number of cells and number of reads per cell to analyze based on previous publications: Tabula Muris (Nature, 2018), Zanini et al. (PNAS, 2018), Zanini et al. (eLife, 2018). The number of time points and mice per time point was chosen to maximize breath/diversity while having more than 2 time points to ensure robust detection of temporal patterns (e.g. pseudo- vs real time).

Replicates

- You should report how often each experiment was performed
- You should include a definition of biological versus technical replication
- The data obtained should be provided and sufficient information should be provided to indicate the number of independent biological and/or technical replicates
- If you encountered any outliers, you should describe how these were handled
- Criteria for exclusion/inclusion of data should be clearly stated
- High-throughput sequence data should be uploaded before submission, with a private link for reviewers provided (these are available from both GEO and ArrayExpress)

Please outline where this information can be found within the submission (e.g., sections or figure legends), or explain why this information doesn't apply to your submission:

eLife Sciences Publications, Ltd is a limited liability non-profit non-stock corporation incorporated in the State of Delaware, USA, with company number 5030732, and is registered in the UK with company number FC030576 and branch number BR015634 at the address 1st Floor, 24 Hills Road, Cambridge CB2 1JP | August 2014

For the single cell studies, each cell is a replicate in its own right. To minimize batch effects, two mice per time point and two plates per mouse were processed across several sequencing runs. Low quality cells were excluded by filtering out cells containing less than 50,000 reads and 400 genes per cell, as shown in the freely available code and stated in the Methods. Non-immune cells were filtered via unsupervised clustering as shown in the code. For the in situ validation studies, and the flow cytometry studies, each individual mouse was a separate biologic replicate. The number of mice used for the studies are detailed in the Figure Legends. Outliers were not removed, and the data was presented as whisker-dot plots to fully disclose the biologic variation between mice.

Statistical reporting

- Statistical analysis methods should be described and justified
- Raw data should be presented in figures whenever informative to do so (typically when N per group is less than 10)
- For each experiment, you should identify the statistical tests used, exact values of N, definitions of center, methods of multiple test correction, and dispersion and precision measures (e.g., mean, median, SD, SEM, confidence intervals; and, for the major substantive results, a measure of effect size (e.g., Pearson's r, Cohen's d)
- Report exact p-values wherever possible alongside the summary statistics and 95% confidence intervals. These should be reported for all key questions and not only when the p-value is less than 0.05.

Please outline where this information can be found within the submission (e.g., sections or figure legends), or explain why this information doesn't apply to your submission:

All statistical analyses are described in the Main text and in the Methods. N numbers and names of the statistical tests are also contained in the Figure Legends where applicable. The code and data to reproduce the analyses is freely available as stated in the Methods.

(For large datasets, or papers with a very large number of statistical tests, you may upload a single table file with tests, Ns, etc., with reference to sections in the manuscript.)

Group allocation

- Indicate how samples were allocated into experimental groups (in the case of clinical studies, please specify allocation to treatment method); if randomization was used, please also state if restricted randomization was applied
- Indicate if masking was used during group allocation, data collection and/or data analysis

Please outline where this information can be found within the submission (e.g., sections or figure legends), or explain why this information doesn't apply to your submission:

Female and male mice were randomly selected at each timepoint. Tissue for the in situ studies were obtained from both male and female mice from multiple litters to limit litter-specific effects. These details are included in the Methods.

Additional data files ("source data")

- We encourage you to upload relevant additional data files, such as numerical data that are represented as a graph in a figure, or as a summary table

eLife Sciences Publications, Ltd is a limited liability non-profit non-stock corporation incorporated in the State of Delaware, USA, with company number 5030732, and is registered in the UK with company number FC030576 and branch number BR015634 at the address 1st Floor, 24 Hills Road, Cambridge CB2 1JP | August 2014

- Where provided, these should be in the most useful format, and they can be uploaded as “Source data” files linked to a main figure or table
- Include model definition files including the full list of parameters used
- Include code used for data analysis (e.g., R, MatLab)
- Avoid stating that data files are “available upon request”

Please indicate the figures or tables for which source data files have been provided:

The count and metadata tables as well as the scripts to generate the figures for the single cell data are all available online (on FigShare and GEO) as stated in the Methods.
